# Accurate prediction of cucurbituril binding affinities from guest molecular formulae

**DOI:** 10.1039/d6sc00680a

**Published:** 2026-03-02

**Authors:** Josie Franks, Eric Masson

**Affiliations:** a Department of Chemistry and Biochemistry, Ohio University Athens Ohio 45701 USA masson@ohio.edu

## Abstract

The binding affinities of 24 ammonium salts were determined by isothermal titration calorimetry (ITC). The affinities were predicted with three empirical models that do not invoke the macrocycle explicitly. Once head groups common to all guests are removed, and the remaining fragments are transferred from aqueous solution to a virtual gas phase, the free energy of transfer from the gas phase to the CB[7] cavity is correlated with (a) the dispersive component of their interaction with a non-polar, non-polarizable hard sphere fluid, (b) their free energy of solvation in tetramethylglycoluril, that mimics the main building block of CB[7], and (c) a sum of individual guest atom free energy contributions *via* multiple linear regression. The latter method, which simply correlates binding affinities with the guest molecular formulae, is the most precise and accurate one, except for tight-fitting guests, with mean absolute errors as low as 0.16 kcal mol^−1^, thereby rivaling experimental error (0.06 kcal mol^−1^ on average). Accuracy exceeds density functional theory calculations that treat the host explicitly.

## Abstract 2

Three empirical models were developed to predict in mere seconds the binding affinities of 39 loose-fitting guests to Cucurbit[7]uril without ever invoking the macrocycle.

## Graphical abstract

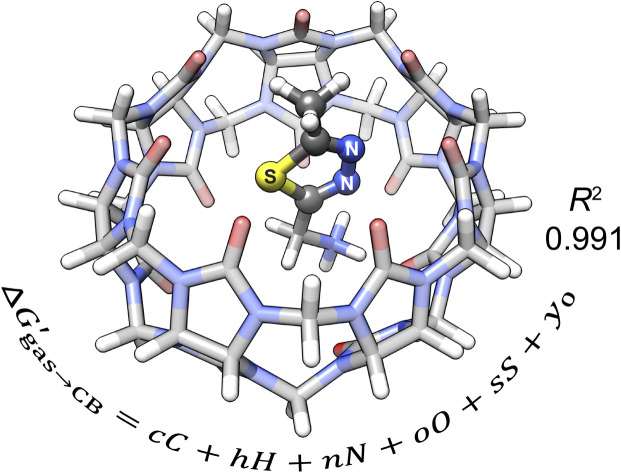

## Introduction

While the ejection of sub-optimally organized water molecules from the cavity of cucurbiturils (CB[*n*])^1–4^ to bulk water is the main driving force of guest encapsulation, desolvation of the guests and coulombic and dispersive interactions between the guests and the CB[*n*] cavity determine selectivity, *i.e.* variations, sometimes subtle, in binding affinities (for an in-depth assessment of the impact of water displacement on host–guest recognition, see the recent work by Biedermann, Nau and Gilson).^5^ A few years ago, we designed a model^6^ that can predict the relative binding affinities of a series of hydrocarbons^7–12^ and noble gases to CB[5], CB[6], CB[7], and to CB[8] already interacting with an auxiliary guest, without explicitly invoking the macrocycles. The free energy of transfer of the hydrocarbon guest from a virtual gas phase to the cavity of CB[*n*] Δ*G*_gas→CB_ is obtained from eqn (1); Δ*G*_aq→CB_ originates from the experimentally determined binding affinity *K*_aq→CB_ (see eqn (2)) and 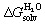 is its free energy of solvation in water calculated by density functional theory (DFT) and the COSMO-RS solvation model.^13^1
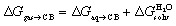

2 Δ*G*_aq→CB_ = −*RT* ln *K*_aq→CB_

CB[*n*]s are then mimicked with a hard sphere fluid of low polarity (such as perfluorohexane). A cavity that will later accommodate the guest is drilled into the hard sphere fluid; the free energy required to that effect is the cavitation energy calculated using the Boublik–Mansoori–Carnahan–Starling–Leland equation of state (see eqn (3)).^14–18^3
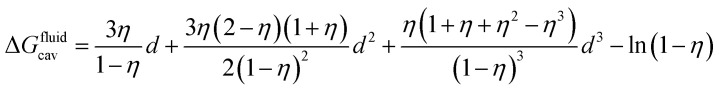


The solute–solvent diameter ratio *d* is obtained from eqn (4), with *σ*_solute_ and *σ*_solvent_ being their respective diameters as a hard sphere expressed in Å obtained using eqn (5); *η* is the solvent packing fraction obtained from eqn (6); *N*_A_ is the Avogadro constant, *ρ* its density in g cm^−3^ and *M* its molar mass. In eqn (5), *V* is the volume of the solute or solvent expressed in Å^3^, calculated from a structure delimited by a 0.002 electron per Bohr^3^ isodensity surface after optimization with the PM6 semi-empirical model; *c* is a calibration constant (0.922; see our earlier study^6^ for details).

4 *d* = *σ*_solute_/*σ*_solvent_

5
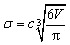
6
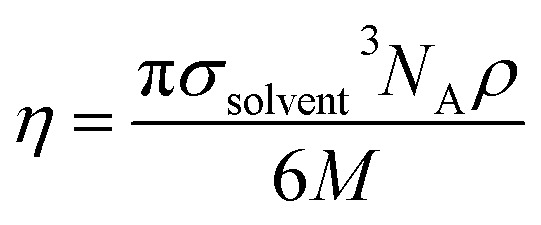


The free energy of transfer of the hydrocarbon guest from the gas phase to the cavity of CB[*n*] is then plotted as a function of the free energy of transfer of the guest to the cavity drilled into the hard sphere fluid Δ*G*_gas→fluid_ (see eqn (7)).

7 Δ*G*_gas→fluid_ = Δ*G*^fluid^_disp_ = Δ*G*^fluid^_solv_ − Δ*G*^fluid^_cav_

**Fig. 1:**
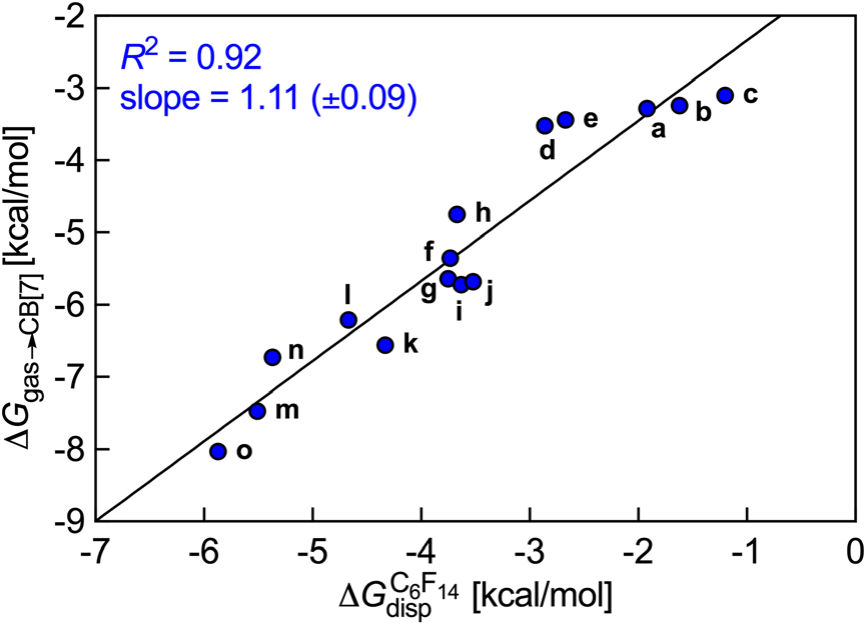
Free energy of transfer of hydrocarbons a–o from the gas phase to the CB[7] cavity as a function of the dispersive component of their interaction with perfluorohexane. Ethane (a), ethylene (b), acetylene (c), propane (d), propene (e), butane (f), *cis*-butene (g), *trans*-butene (h), isobutane (i), isobutene (j), neopentane (k), cyclopentane (l), cyclohexane (m), benzene (n), norbornene (o).^10^

Δ*G*_gas→fluid_ corresponds to the dispersive interaction Δ*G*^fluid^_disp_ between the hydrocarbon guest and the hard sphere fluid, and is calculated by subtracting the cavitation energy Δ*G*^fluid^_cav_ from the free energy of solvation Δ*G*^fluid^_solv_ of the guest in the low polarity hard sphere fluid, also obtained using the COSMO-RS model. Linear regression lines with slopes near unity and consistently excellent *R*^2^ coefficients allow the extrapolation of binding affinities of various hydrocarbons towards these hosts (see Fig. 1; binding affinities of hydrocarbons towards CB[7] recorded by Nau and coworkers).^10^

In this study, we will test the scope, performance and limitations of the hard sphere fluid model with a set of more diverse guests (24 ammonium salts, mono-substituted with alkyl, benzyl and various heterocyclic groups). To address its limitations, two new models will be proposed that return both accurate and precise binding affinities for the new set of guests, as well as for the previously reported hydrocarbons. A feature and self-imposed limitation of all models are that CB[*n*]s are never considered explicitly, thereby dramatically shortening calculation times.

## Results

**Fig. 2:**
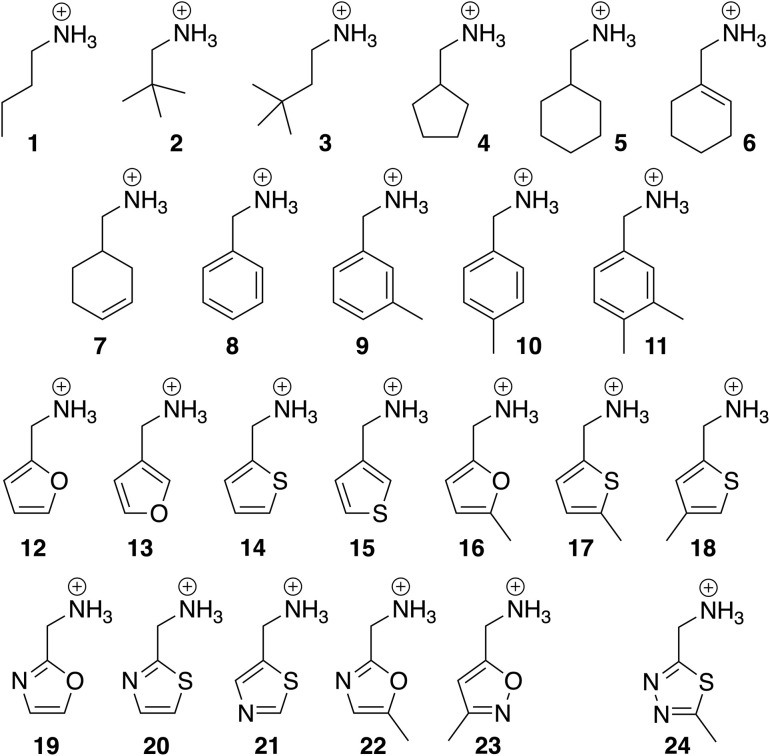
Structures of CB[7] guests 1–24. Chloride as counteranion throughout the series.

The binding affinities of CB[7] towards guests 1–24 (see Fig. 2 and Table 1, all commercially available as either chloride salts or free amines that are subsequently protonated) were determined by isothermal titration calorimetry (ITC) in triplicates. Encapsulation was confirmed by ^1^H nuclear magnetic resonance spectroscopy (NMR), as signals of hydrogen nuclei located inside CB[7] undergo characteristic upfield shifts (see SI section for all enthalpograms and NMR titrations). Binding affinities ranged from 6.4 × 10^4^ M^−1^ for the small polar oxazole 19 to 3.4 × 10^6^ M^−1^ for the larger hydrophobic 3,4-dimethylbenzyl derivative 11 (see Table 1). Binding affinities were then converted into free Gibbs energy terms using eqn (2). Those ranged from −6.56 to −8.90 kcal mol^−1^ (see Table 1). ITC measurements were highly reproducible, with an average error of 0.06 kcal mol^−1^ (10% in terms of binding affinities, see SI section for details) for both the fitting of each enthalpogram with a 1 : 1 host–guest complexation model and the average of the triplicate runs.

**Table 1:** Binding affinities of guests 1–24 to CB[7]; free energy terms associated with the recognition process and the empirical prediction models

	*K*_aq→CB_ a	Δ*G*_aq→CB_^b^	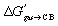 c	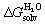 d	Δ*G*^predicted^_HS fluid_^e^		Δ*G*^predicted^_TMG solv_^f^		Δ*G*^predicted^_MLR_^g^		Δ*G*^CB[7]^_disp_^h^
1	8.3 (±0.1) × 10^5^	−8.08 (±0.04)	−6.44	1.63	−5.95	(+0.5)	−6.28	(+0.2)	−6.41	(0.0)	−12.51
2	1.5 (±0.1) × 10^6^	−8.41 (±0.08)	−6.71	1.70	−6.69	(0.0)	−6.63	(+0.1)	−6.66	(0.0)	−18.20
3	1.6 (±0.1) × 10^6^	−8.47 (±0.11)	−6.80	1.66	−7.36	(–0.6)	−6.96	(–0.2)	−6.91	(–0.1)	−20.48
4	1.4 (±0.1) × 10^6^	−8.40 (±0.07)	−7.35	1.05	−7.67	(–0.3)	−7.40	(–0.1)	−7.43	(–0.1)	−19.18
5	2.0 (±0.1) × 10^6^	−8.60 (±0.02)	−7.61	0.99	−8.47	(–0.9)	−7.82	(–0.2)	−7.68	(–0.1)	−22.39
6	1.4 (±0.1) × 10^6^	−8.40 (±0.06)	−8.31	0.09	−8.44	(–0.1)	−8.27	(0.0)	−8.19	(+0.1)	−21.62
7	1.5 (±0.1) × 10^6^	−8.44 (±0.02)	−8.36	0.08	−8.45	(–0.1)	−8.28	(+0.1)	−8.19	(+0.2)	−22.01
8	1.2 (±0.1) × 10^6^	−8.31 (±0.04)	−9.21	−0.91	−8.34	(+0.9)	−9.06	(+0.2)	−9.23	(0.0)	−20.88
9	1.7 (±0.1) × 10^6^	−8.50 (±0.11)	−9.35	−0.85	−9.06	(+0.3)	−9.38	(0.0)	−9.47	(–0.1)	−23.46
10	1.3 (±0.1) × 10^6^	−8.34 (±0.08)	−9.17	−0.83	−9.06	(+0.1)	−9.38	(–0.2)	−9.47	(–0.3)	−20.85
11	3.4 (±0.2) × 10^6^	−8.90 (±0.12)	−10.06	−1.15	−9.88	(+0.2)	−9.91	(+0.2)	−9.73	(+0.3)	−24.03
12	6.1 (±0.1) × 10^5^	−7.89 (±0.01)	−8.91	−1.03	−9.05	(–0.1)	−8.49	(+0.4)	−8.63	(+0.3)	
13	5.1 (±0.1) × 10^5^	−7.79 (±0.01)	−8.81	−1.02	−9.05	(–0.2)	−8.49	(+0.3)	−8.63	(+0.2)	
14	1.3 (±0.1) × 10^6^	−8.33 (±0.01)	−9.92	−1.59	−10.79	(–0.9)	−10.20	(–0.3)	−9.81	(+0.1)	
15	9.8 (±0.4) × 10^5^	−8.17 (±0.02)	−9.77	−1.59	−10.79	(–1.0)	−10.20	(–0.4)	−9.81	(0.0)	
16	7.9 (±0.3) × 10^5^	−8.04 (±0.07)	−8.94	−0.90	−10.15	(–1.2)	−9.02	(–0.1)	−8.88	(+0.1)	
17	1.6 (±0.1) × 10^6^	−8.47 (±0.06)	−9.83	−1.36	−11.79	(–2.0)	−10.59	(–0.8)	−10.06	(–0.2)	
18	1.6 (±0.1) × 10^6^	−8.48 (±0.05)	−9.91	−1.43	−11.79	(–1.9)	−10.69	(–0.8)	−10.06	(–0.2)	
19	6.4 (±0.1) × 10^4^	−6.56 (±0.01)	−10.57	−4.01	−9.27	(+1.3)	−10.01	(+0.6)	−10.96	(–0.4)	
20	4.7 (±0.2) × 10^5^	−7.73 (±0.08)	−12.32	−4.59	−11.07	(+1.3)	−11.71	(+0.6)	−12.15	(+0.2)	
21	3.2 (±0.1) × 10^5^	−7.50 (±0.01)	−12.08	−4.58	−11.07	(+1.0)	−11.71	(+0.4)	−12.15	(–0.1)	
22	8.1 (±0.1) × 10^4^	−6.70 (±0.06)	−10.94	−4.24	−10.56	(+0.4)	−10.84	(+0.1)	−11.21	(–0.3)	
23	1.5 (±0.1) × 10^5^	−7.08 (±0.02)	−11.36	−4.28	−10.43	(+0.9)	−11.55	(–0.2)	−11.21	(+0.1)	
24	1.7 (±0.1) × 10^5^	−7.15 (±0.20)	−14.94	−7.79	−12.51	(+2.4)	−14.80	(+0.1)	−14.73	(+0.2)	

a Binding affinity [M^−1^] obtained by ITC (average of triplicates; errors cover both fitting errors of each enthalpogram and the error on the average; see SI section for details).

b Free energy of guest transfer from aqueous solution to CB[7].

c Relative free energy of guest transfer from gas phase to CB[7].

d Free energy of solvation of guests (CH_2_NH_3_^+^ group truncated) in water. Predicted relative free energy of guest transfer from gas phase to CB[7] using.

e The hard–sphere fluid model.

f The free energies of solvation of guests (CH_2_NH_3_^+^ group truncated) in tetramethylglycoluril (TMG).

g Multiple linear regression analysis (MLR). Deviation with experimental values between parentheses.

h Dispersive component of the guest/CB[7] interaction. All energy terms in kcal mol^−1^.

## Discussion

Our first objective was to test our hard sphere fluid model towards CB[7] and guests 1–24. As all share the same CH_2_NH_3_^+^ anchor that protrudes out of the cavity, interacts with the carbonylated rim of CB[7] and secures the other moiety into the cavity, our first approximation was to consider its contribution to the binding free energy as constant throughout the series. The CH_2_NH_3_^+^ groups were thus truncated and replaced with a hydrogen atom to generate a new set of neutral scaffolds.

To extract the intrinsic affinity of the neutral guests towards CB[7], as mentioned earlier, they must be dehydrated first *in silico*. To that aim, solvation energies in water 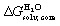 were calculated using the COSMO-RS model with the COSMOthermX23 software, after optimization at the BP86 level with Ahlrichs def2-TZVP basis sets and single point energy calculations at the BP86/def2-TZVPD level using the Turbomole software (see SI section for details). The intrinsic binding affinity of the neutral truncated guests to CB[7], *i.e.* their free energy of transfer from the gas phase to the cavity of CB[7], is determined using eqn (8) (a variant of eqn (1)), where Δ*G*_anchor_ is the unknown but constant contribution of the CH_2_NH_3_^+^ unit:8
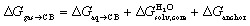


The constant Δ*G*_anchor_ term is then removed to yield eqn (9) and the relative free energy term 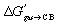.9
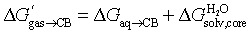


**Fig. 3:**
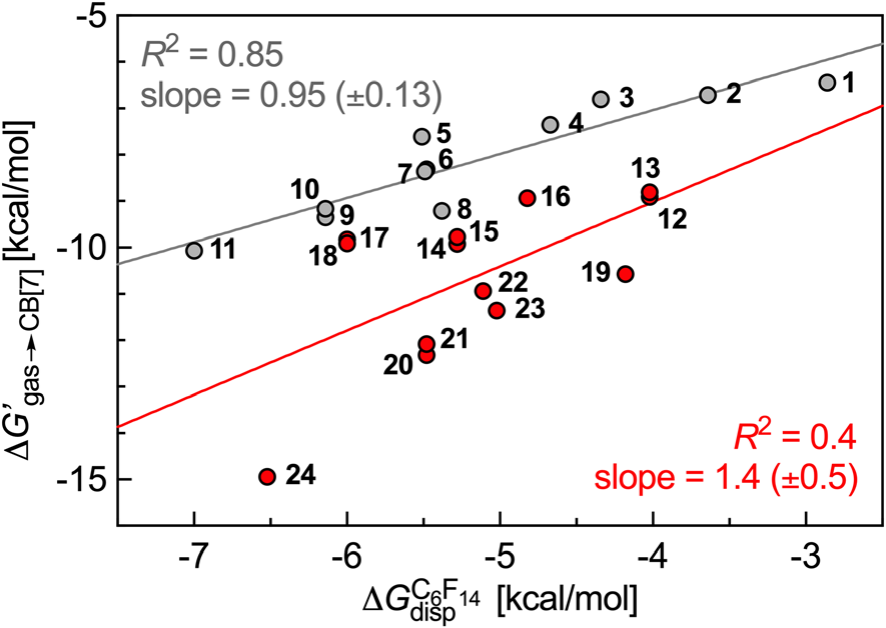
Free energy of transfer of guests 1–24 (CH_2_NH_3_^+^ head group truncated) from the gas phase to the CB[7] cavity as a function of the dispersive component of their interaction with perfluorohexane. Hydrocarbons in grey, heterocycles in red.

The cavity of CB[7] was then mimicked with perfluorohexane as a hard sphere fluid, as reported in our earlier study.^6^ The solvation energies of all neutral guests in perfluorohexane Δ*G*^C6F14^_solv,core_ were obtained as described above using the COSMO-RS method (see SI section for details). The cavitation energies of the neutral guests in perfluorohexane Δ*G*^C6F14^_cav_ were calculated using eqn (3)–(6). Free energy terms 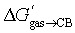 were then plotted as a function of Δ*G*_gas→fluid_ (see eqn (7) and Fig. 3).

Fig. 3 shows that our hard sphere fluid remains valid for mono-substituted ammonium salts 1–11, *i.e.* for those that do not contain any heteroelement; the slope of the regression line is near unity (0.95 ± 0.13), and the *R*^2^ coefficient of 0.85 is satisfactory. The nature of outliers 5 and 8 is problematic, however: the model underestimates the binding affinity of benzylammonium 8 by 0.9 kcal mol^−1^ (a factor of 4.3 in terms of binding affinity), and overestimates the affinity of its unsaturated counterpart 5 by 0.9 kcal mol^−1^. In other terms, while our hard sphere fluid model correctly predicted the higher affinity of cyclohexane compared to benzene towards CB[7] (a 90-fold difference),^10^ it fails to predict the much closer affinity of these two guests when flanked by the CH_2_NH_3_^+^ head group (guest 5 binds CB[7] 1.7 times tighter than guest 8, see Table 1). Fig. 3 also shows that the model is not at all suitable for heterocyclic guests 12–24 (*R*^2^ = 0.4). One likely reason for the collapse of the model is the improper treatment of coulombic interactions between the polar guests and the CB[7] cavity environment: the model considers it as uniformly non-polar, hence the choice of perfluorohexane to mimic the cavity; it thus completely neglects possible direct contact between the polar guests and the polar inner wall of CB[7]. A more realistic view of the CB[7] cavity environment should be a sharply decreasing polarity from the inner wall to the center of the cavity.

**Fig. 4:**
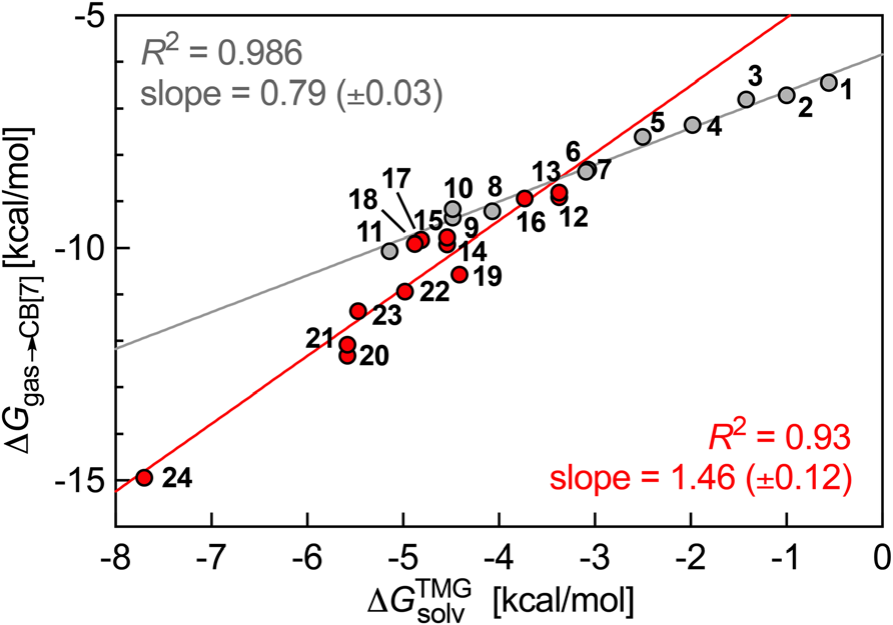
Free energy of transfer of guests 1–24 (CH_2_NH_3_^+^ head group truncated) from the gas phase to the CB[7] cavity as a function of their free energy of solvation in TMG. Hydrocarbons in grey, heterocycles in red.

This non-uniform polarity of the CB[7] cavity prompted us to test a different model that gives much more weight to the impact of the inner wall: could CB[7] be sliced into glycoluril units (tetramethylated, to avoid any undue hydrogen-bonding interactions), that would then loosely interact with the guests, *i.e.* solvate them? In other terms, can the rigidity of the macrocycle be neglected when predicting trends in binding affinity? To answer these questions, we calculated the solvation energy of the neutral truncated analogs of guests 1–24 into molten tetramethylglycoluril (TMG) using the COSMO-RS model (see SI section for details). We then plotted their relative free energy of transfer from the gas phase to CB[7] 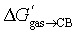 as a function of their free energy of solvation in TMG Δ*G*^TMG^_solv_ (see Fig. 4). This model was found to be extremely accurate for hydrophobic guests 1–11 (*R*^2^ = 0.986 and a mean absolute error (MAE) of only 0.12 kcal mol^−1^!). It also performed very well with heterocyclic guests 12–24 (*R*^2^ = 0.93). The model suffers from one drawback, however: guests 1–24 must be treated as two separate datasets (1–11 and 12–24), as those return different linear regression slopes (0.79 ± 0.03 and 1.5 ± 0.1, respectively). This problem would affect predictive value, as new guests would have to be assigned to a specific set first, and the presence or absence of heteroatoms in the guests might not be the sole parameter to assign them to the most appropriate set.

**Fig. 5:**
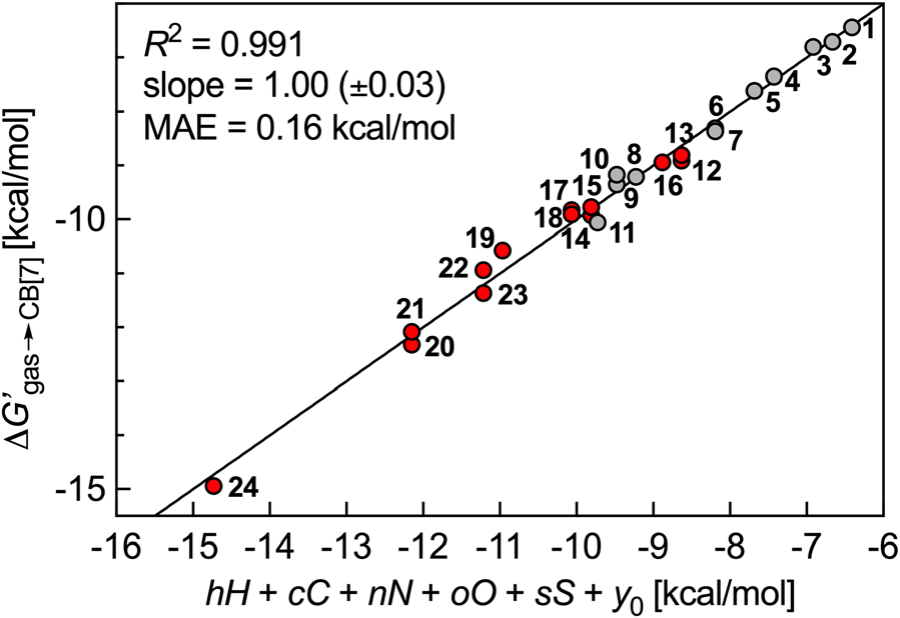
Free energy of transfer of guests 1–24 (CH_2_NH_3_^+^ head group truncated) from the gas phase to the CB[7] cavity as a function of a sum of individual guest atom contributions. Hydrocarbons in grey, heterocycles in red.

We then questioned again how the coulombic interaction between polar guests such as 11–24 and the CB[7] inner wall could be factored in empirically. This led us to attempt an extremely rudimentary correlation: could their relative free energy of transfer from gas phase to CB[7] 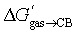 correlate with a weighted linear combination of the number of each atom (*C*, *H*, *N*, *O* and *S*) in the guests (see eqn (10), where *c*, *h*, *n*, *o* and *s* are the weights and *y*_0_ is the *y*-axis intercept)? In crude terms: can one extract a binding affinity from the guest formula?10
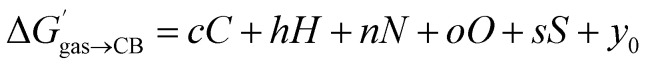
To our surprise, this multiple linear regression (MLR) returned an *R*^2^ coefficient of 0.991, with a MAE of only 0.16 kcal mol^−1^, exceptionally close to experimental error (0.06 kcal mol^−1^)! Weights *c*, *h*, *n*, *o* and *s* (see Table 2) are the free energy contribution of each atom to the free energy of transfer 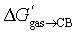: larger guests (with more carbon atoms) benefit from enhanced dispersive interactions with the CB[7] walls (−0.77 kcal per mol per C atom), while guest saturation is mildly penalizing (+0.26 kcal per mol per H atom). Nitrogens afford significant coulombic interactions (−2.84 kcal per mol per N atom), while the larger size and more polarizable nature of sulfur atoms contribute more than oxygens (−1.60 kcal per mol per S atom *vs.* −0.42 kcal per mol per O atom) (Fig. 5).

**Table 2:** Coefficients representing the free energy contribution of each atom, as well as the guest charge, to the CB[7] binding affinities (24 and 39-guest datasets)

Guests	*c*	*h*	*n*	*o*	*s*	*z*
1–24	−0.77 (±0.04)	+0.26 (±0.03)	−2.84 (±0.10)	−0.42 (±0.19)	−1.60 (±0.20)	
1–24	−0.91 (±0.07)	+0.01 (±0.04)	−3.13 (±0.22)	−1.73 (±0.32)	−2.96 (±0.32)	−1.39 (±0.22)
+a–o^a^						

a From ref. 10 See Fig. 1 caption for the list of guests.

**Fig. 6:**
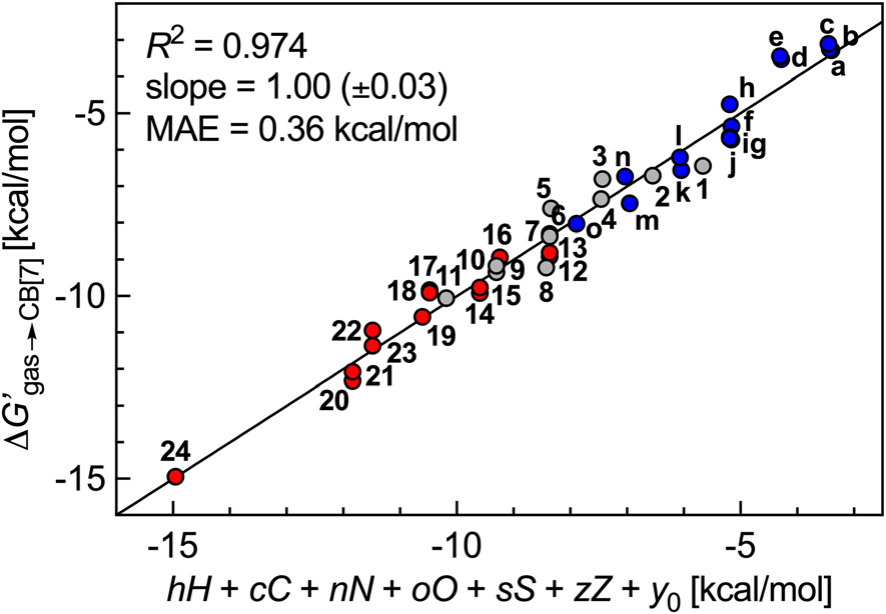
Free energy of transfer of guests 1–24 (CH_2_NH_3_^+^ head group truncated) and hydrocarbons a–o (see Fig. 1 caption for the list of compounds) from the gas phase to the CB[7] cavity as a function of a sum of individual guest atom contributions and charge. Series a–o in blue, hydrocarbons 1–11 in grey, heterocycles 12–24 in red.

The success of this method prompted us to add 15 hydrocarbons evaluated by Nau and coworkers^10^ to the data set. It now contains free energy terms for neutral hydrocarbons a–o (see Fig. 1 caption) and positively charged guests 1–24, therefore an additional term *Z* = 0 or 1 is required in the linear combination to account for the impact of the charge on the affinities (or more precisely, of the CH_2_NH_3_^+^ head group; see eqn (11)). Multiple linear regression was again highly precise (*R*^2^ = 0.974, see Fig. 6) with a MAE of 0.36 kcal mol^−1^ over all 39 guests.11
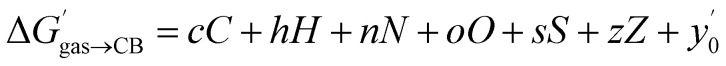


We note that coefficient *z* is unexpectedly small (a −1.39 kcal mol^−1^ contribution per positive head group); for example, the binding affinities of cyclohexylmethylammonium (5) and cyclohexane (m)^10^ are comparable (2.2 × 10^6^*vs.* 1.5 × 10^6^ M^−1^). Large affinity enhancements^19^ measured upon attachment of positive head groups to extremely tight-binding guests such as adamantane, ferrocene, bicyclo[2.2.2]octane and diamantane cores might therefore not necessarily be the norm.

These tight-binding guests deserve additional comments, as all our models underestimate their affinities. The binding affinity of adamantane (25a) to CB[7]^10^ is 2.2 × 10^9^ M^−1^ while our hard-sphere fluid and formula-based models predict affinities of 3.6 × 10^7^ M^−1^ and 6.8 × 10^7^ M^−1^, respectively. Attaching the CH_2_NH_3_^+^ head group (guest 25b) dramatically increases the affinity to 7.7 × 10^14^ M^−1^,^19^ while our hard-sphere fluid, TMG solvation and molecular formulae models predict affinities of 1.2 × 10^8^, 8.9 × 10^6^ and 7.0 × 10^8^ M^−1^, respectively.

One can readily show that none of the models accounts for the dramatic increase in dispersive interactions between tight-fitting guests and the inner CB[7] wall. We optimized the structures of complexes 1 CB[7]–11 CB[7], 25b CB[7] and 26b CB[7] using the semi-empirical GFN2-xTB method in conjunction with the ALPB solvation model for water,^20–23^ then carried out energy decomposition analysis^24^ with the SCM AMS software using the ZORA-pbe-D3(BJ) functional and TZ2P basis sets to determine the dispersive component of the host-guest interaction (see Δ*G*^CB[7]^_disp_ in Table 1, and SI section for details). A 13.3 kcal mol^−1^ enhancement in dispersive interactions is calculated between CB[7]-bound 1-adamantylmethyl-ammonium 25b CB[7] (−33.8 kcal mol^−1^, see Table 3) and the average dispersive interactions of complexes 1 CB[7]–11 CB[7] (−20.5 kcal mol^−1^); adding this extra contribution to the average free energy of transfer from gas phase to the CB[7] cavity 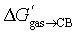 for these guests (−8.1 kcal mol^−1^) returns −21.4 kcal mol^−1^, very close to the experimental 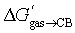 for guest 25b (−20.2 kcal mol^−1^, see Table 1). The dramatic increase in binding affinity of adamantyl guests 25 is thus mostly dispersion driven.

**Table 3:** Binding affinities of guests 25 and 26 to CB[7]; energy terms associated with the recognition process, the empirical prediction models, and the energy decomposition analysis

	*K*_aq→CB_ a	Δ*G*_aq→CB_^b^	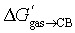 c	Δ*G*^predicted^_MLR_^d^	Δ*E*_Pauli_^e^	Δ*E*_electrostatic_^e^	Δ*E*_orbital_^e^	Δ*E*_disp_^e^	Δ*E*_total_^e^
25a	2.2 × 10^9^	−12.74	−12.59	−10.53	27.7	−16.3	−11.5	−30.1	−30.2
26a	1.6 × 10^9^	−12.56	−13.03	−14.11	56.0	−29.6	−17.2	−37.1	−27.9
25b	7.7 × 10^14^	−20.30	−20.16	−11.92	50.0	−74.9	−34.6	−33.8	−93.3
26b	f	f	f	−15.50	71.8	−85.0	−37.7	−40.7	−91.6

a Binding affinity [M^−1^].^10,19^

b Free energy of guest transfer from aqueous solution to CB[7].

c Relative free energy of guest transfer from gas phase to CB[7].

d Predicted relative free energy of guest transfer from gas phase to CB[7] using multiple linear regression analysis (MLR).

e See eqn (12) and narrative.

f No experimental data; *in silico* assessment only. All energy terms in kcal mol^−1^.

The correlation between the number of carbon and hydrogen atoms and 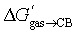 terms breaks down also with tight-fitting guests. As shown by Nau,^10^ the binding affinities of adamantane (25a; C_10_H_16_) and diamantane (26a; C_14_H_20_) to CB[7] are very similar (2.2 × 10^9^ and 1.6 × 10^9^ M^−1^, respectively); 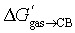 are −12.59 and −13.03 kcal mol^−1^ (see Table 3). The molecular formulae model predicts a much tighter binding for diamantane (26a) than adamantane (25a), as it contains more carbon atoms (1.0 × 10^10^ M^−1^*vs.* 6.8 × 10^7^ M^−1^, respectively, see Table 3).

To understand why binding affinities are very similar, complex optimization and energy decomposition analysis were again carried out as discussed above to isolate the different contributors to the CB[7]-guest interaction using eqn (12); Δ*E*_Pauli_ is the penalty for the antisymmetrization of the fragments wavefunctions, *i.e.* the exchange repulsion between occupied orbitals on the host and the guest (the main contributor to steric repulsion), Δ*E*_electrostatic_ is the attractive coulombic term, Δ*E*_orbital_ is the stabilization caused by relaxation and mixing of the host and guest orbitals, Δ*E*_disp_ is the dispersive component of the interaction, and Δ*E*_total_ is the sum of all energy contributions in the gas phase at 0 K.

12 Δ*E*_total_ = Δ*E*_Pauli_ + Δ*E*_electrostatic_ + Δ*E*_orbital_ + Δ*E*_disp_

In line with experiment, Δ*E*_total_ is similar in the adamantane (25a and 25b) and diamantane (26a and 26b) series (−30.2 *vs.* −27.9 kcal mol^−1^ for neutral guests, −93.3 *vs.* −91.6 kcal mol^−1^ for the positive ones). While electrostatic, orbital overlap and dispersion components are more favorable in the diamantane series (by 11.7, 4.5 and 7.0 kcal mol^−1^, respectively and on average), a severe Pauli repulsion penalty (25.1 kcal mol^−1^ on average, see Table 3) is imposed due to compression of the diamantane guests inside the hosts. Our CB-free empirical models cannot take this compression into account. Furthermore, any slight change of geometry in these compressed guests can have a dramatic impact on affinities or on binding at all, as they might no longer fully fit in the cavity. In subsequent studies, we will attempt to identify, synthesize and assess guests that are larger than guests 1–24, but smaller than adamantane. Empirical relationships 10 and 11 might then be fine-tuned with non-linear terms to correct for abnormally favorable electrostatic, orbital and dispersive contributions, and abnormally penalizing steric ones, as packing coefficients exceed Rebek's optimal 55%.^25^

## Conclusions

We proposed here three empirical models that can predict in mere seconds the binding affinities of 39 loose-fitting guests to CB[7] without ever invoking the macrocycle. The most successful one is disarmingly simple: the guest is treated as a core fragment encapsulated inside the macrocycle with a protruding head group. The molecular formula of the core fragment is entered into a weighted linear combination of energy contributions per atom to obtain the energy of transfer of the guest from gas phase to the CB[7] cavity 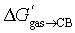 (see eqn (10) and (11)). After subtraction of the free energy of solvation of the guest in water using the COSMO-RS solvation model, the resulting energy term is converted to the binding affinity *K*_aq→CB_ (see eqn (1) and (2)). MAE is as low as 0.16 kcal mol^−1^, remarkably close to experimental error (0.06 kcal mol^−1^ in our series), and significantly lower than state-of-the-art DFT calculations treating the macrocycle explicitly (1.4 kcal mol^−1^ for the **a–o** series at the PW6B95-D3^ATM^/def2-QZVP′//PBEh-3c//COSMO-RS(13) level).^10^

**Fig. 7:**
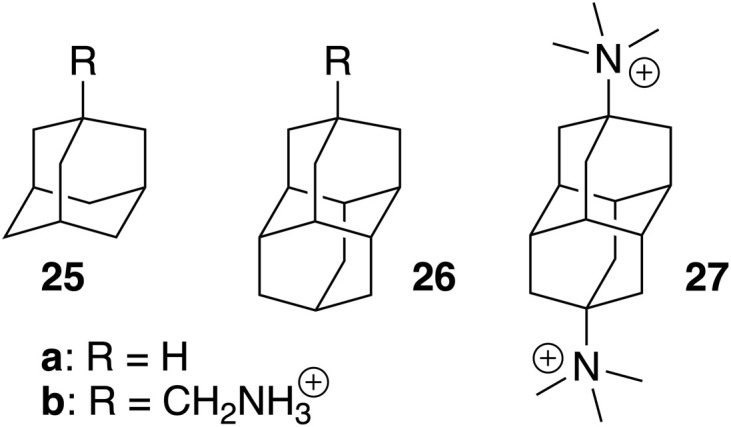
Tight-fitting CB[7] guests with extreme binding affinities.

We should acknowledge the limitations of the proposed models: (1) the range of tested binding affinities in solution *K*_aq→CB_, albeit statistically significant, is limited (the affinity ratio between the strongest and weakest binding guests is 52, a 2.4 kcal mol^−1^ free energy difference). However, the range of free energies of transfer from the gas phase to the cavity of CB[7] 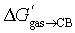 increases dramatically to 8.5 kcal mol; (2) the models currently cannot predict the extreme affinities of tight-fitting guests, however. Adamantane and diamantane core fragments have similar binding affinities and likely represent the best fitting guests for CB[7] due to an optimal balance between favorable dispersive, orbital overlap and coulombic interactions, and unfavorable steric repulsion. In our opinion, it would not be surprising if Isaacs and co-workers had reached an upper limit in binding affinities with their diamantane derivative 27 (see Fig. 7; 7.2 × 10^17^ M^−1^),^26^ which remains the tightest non-covalent interaction ever measured in any host-guest pair in water. In subsequent studies, our models will be tested towards (1) guests bearing the same CB[7]-binding core, but different head groups (we only explored CH_2_NH_3_^+^ in this study) to determine whether those can really be treated as group increments, (2) other tight-fitting guests with volumes slightly smaller than adamantane, yet with unusually strong interactions (favorable or unfavorable) with the inner wall of CB[7], (3) guests bearing other atoms (such as halogens) and specific functional groups that might interact strongly with the macrocycle inner wall and portals (such as hydrogen bond donors, carbonyls, *etc.*), and (4) guests that protrude out of the cavity.

## Author contributions

E. M. conceived the project, designed the models, performed all calculations, and mentored J. F. J. F. carried out all experimental work. E. M. and J. F. wrote the manuscript.

## Conflicts of interest

There are no conflicts to declare.

## Supplementary Material

SC-017-D6SC00680A-s001

## Data Availability

All analytical and computational data are provided in the narrative and in the supplementary information (SI). Supplementary information: NMR titrations of guests 1–24 with CB[7]. Isothermal titration calorimetry enthalpograms. Theoretical and computational details. See DOI: https://doi.org/10.1039/d6sc00680a.
