# Supplementary material for: Accurate prediction of cucurbituril binding affinities from guest molecular formulae
Source: Chem Sci. 2026 Mar 2;17(17):8712–8. doi: 10.1039/d6sc00680a (PMC12980397; doi:10.1039/d6sc00680a)
Supplement: SC-017-D6SC00680A-s001 [file SC-017-D6SC00680A-s001.pdf]

Supplementary Information (SI) for Chemical Science.
This journal is © The Royal Society of Chemistry 2026

Accurate Prediction of Cucurbituril Binding Affinities from Guest Molecular Formulae

Josie Franks[a] and Eric Masson*[a]

[a] *Department of Chemistry and Biochemistry, Ohio University, Athens, Ohio 45701, United States*

E–mail: masson@ohio.edu

Supporting Information

1. Generalities 1
2. Titration of guests **1** – **24** with CB[7] 1
3. Isothermal Titration Calorimetry 26
4. Computational details 36
5. References 37

1. Generalities

Starting materials were purchased from TCI America (Portland, OR), Oakwood Chemicals (Columbia, SC), Aaron Chemical (San Diego, CA), Combi–Blocks (San Diego, CA), Ambeed Inc. (Arlington Heights, IL), AA Blocks LLC (San Diego, CA), A2B Chem (San Diego, CA), and Cambridge Isotope Laboratories Inc. (Tewksbury, MA). Cucurbit[7]uril (CB[7]) was prepared using known procedures.[1] Characterization by nuclear magnetic resonance spectroscopy (NMR) was carried out using a Bruker Ascend 500 spectrometer (Billerica, MA). Solvent used was D_2O. ^{1}H NMR chemical shifts are reported in parts per million (ppm) and are calibrated with the residual HDO signal of the solvent (4.790 ppm). Coupling constants J are reported in Hertz. Isothermal titration calorimetry (ITC) experiments were performed using a Malvern MicroCal iTC200 calorimeter (Chicago, IL). Ammonium chloride salts **5**, **7** and **9** – **17** were prepared by protonation of their corresponding amine in a large excess of methanolic HCl (3 M, used as solvent), evaporation, and drying under high vacuum.

2. Titration of guests 1 – 24 with CB[7]

Stock solutions of guests (40 mM) were prepared in D_2O (0.50 mL). Aliquots (30 µL) were added to D_2O (0.57 mL) to obtain 2.0 mM solutions in NMR tubes. CB[7] (1.7 mg, 1.2 µmol 1.0 equiv) was added in approximately 0.25 equiv aliquots unless noted otherwise. The NMR tubes were sonicated for 1 min, and the spectra recorded after each addition.

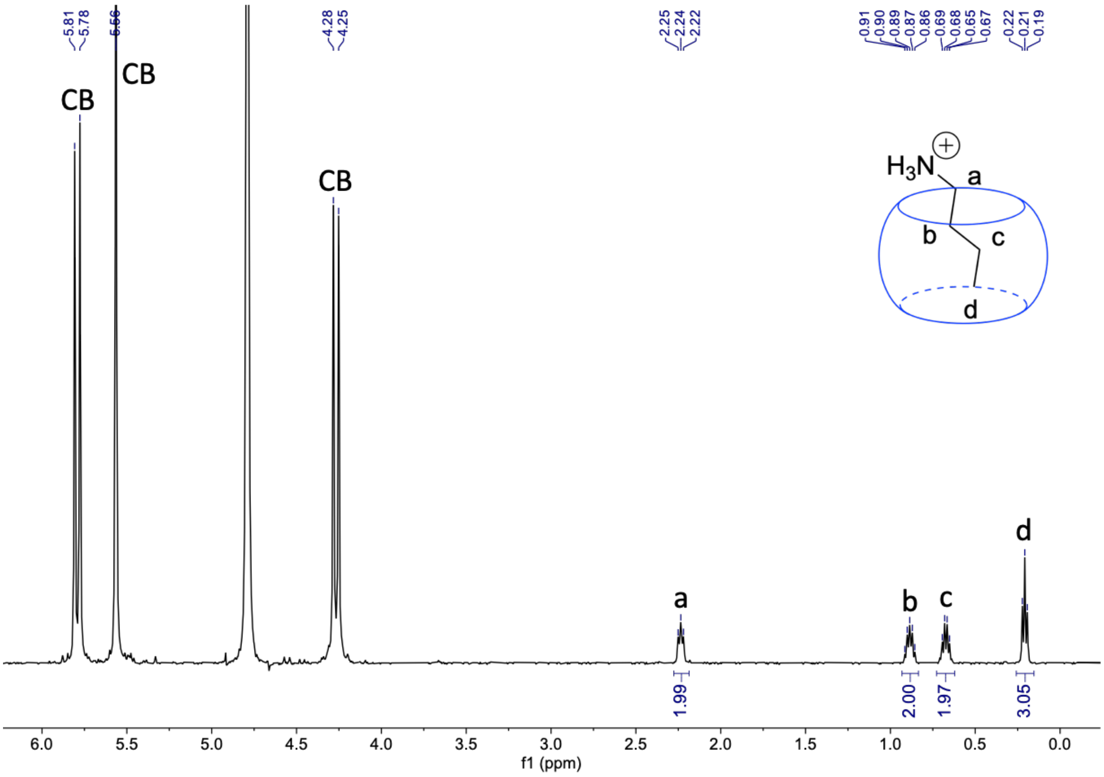

Figure 1. ^{1}H NMR spectrum of complex **1**·CB[7] in D_2O. ^{1}H NMR: δ 2.24 (t, J = 8.0 Hz, 2H, H^a), 0.89 (tt, J = 7.2, 7.1 Hz, 2H, H^b), 0.68 (q, J = 7.2 Hz, 2H, H^c), 0.21 (t, J = 7.4 Hz, 3H, H^d).

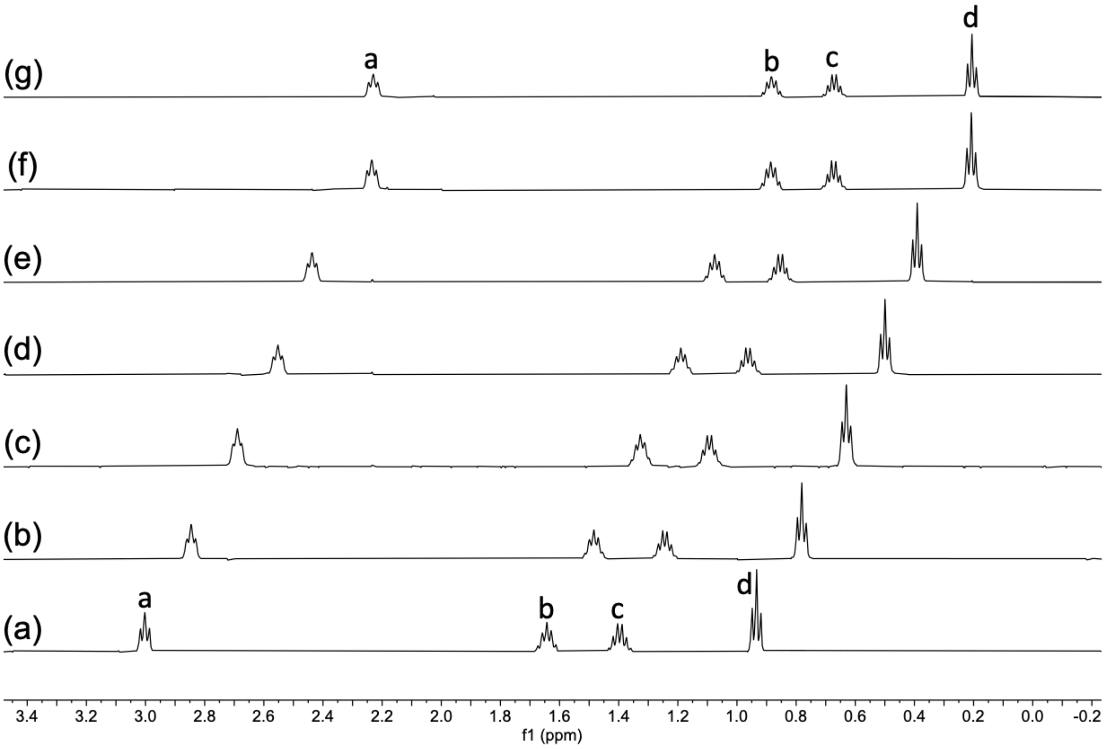

Figure 2. ^{1}H NMR spectra of guest **1** titrated with (a) 0.00, (b) 0.20, (c) 0.40, (d) 0.60, (e) 0.75, (f) 1.00, and (g) 1.50 equiv CB[7] in D_2O.

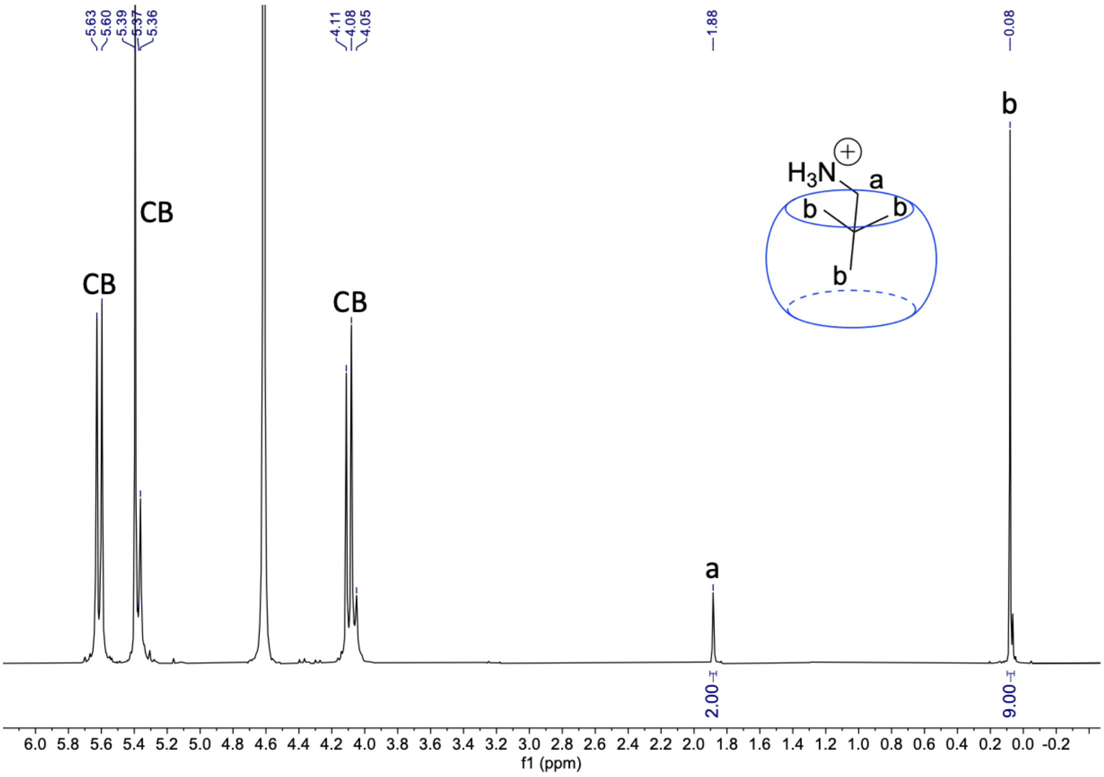

Figure 3. ^{1}H NMR spectrum of complex **2**·CB[7] in D_2O. ^{1}H NMR: δ 1.88 (s, 2H, H^a), 0.08 (s, 9H, H^b).

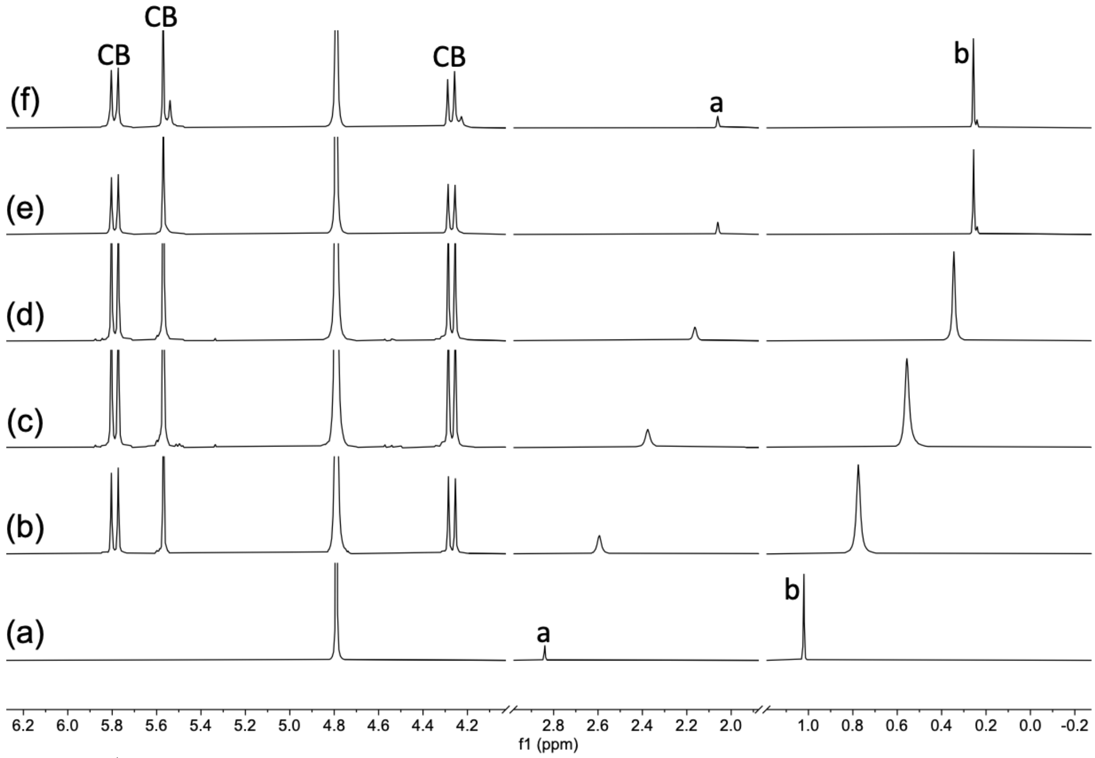

Figure 4. ^{1}H NMR spectra of guest **2** titrated with (a) 0.00, (b) 0.25, (c) 0.50, (d) 0.75, (e) 1.00, and (f) 1.50 equiv CB[7] in D_2O.

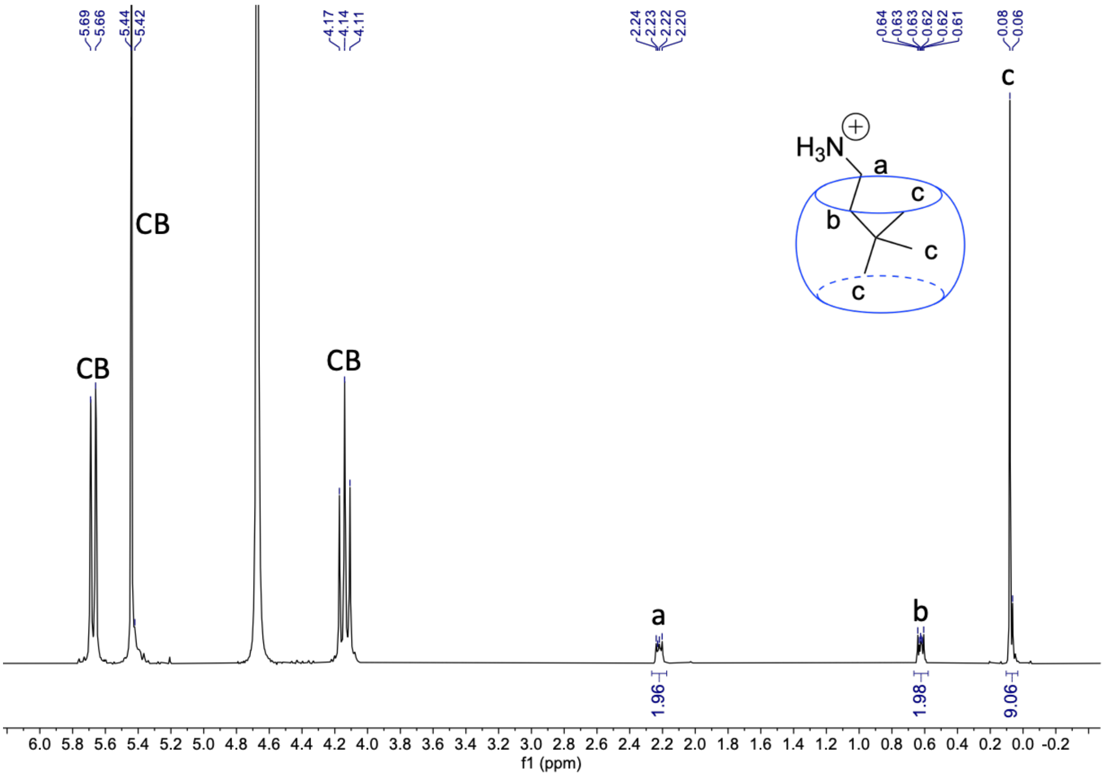

Figure 5. ^{1}H NMR spectrum of complex **3**·CB[7] in D_2O. ^{1}H NMR: δ 2.27 – 2.17 (m, 2H, H^a), 0.67 – 0.58 (m, 2H, H^b), 0.08 (s, 9H, H^c).

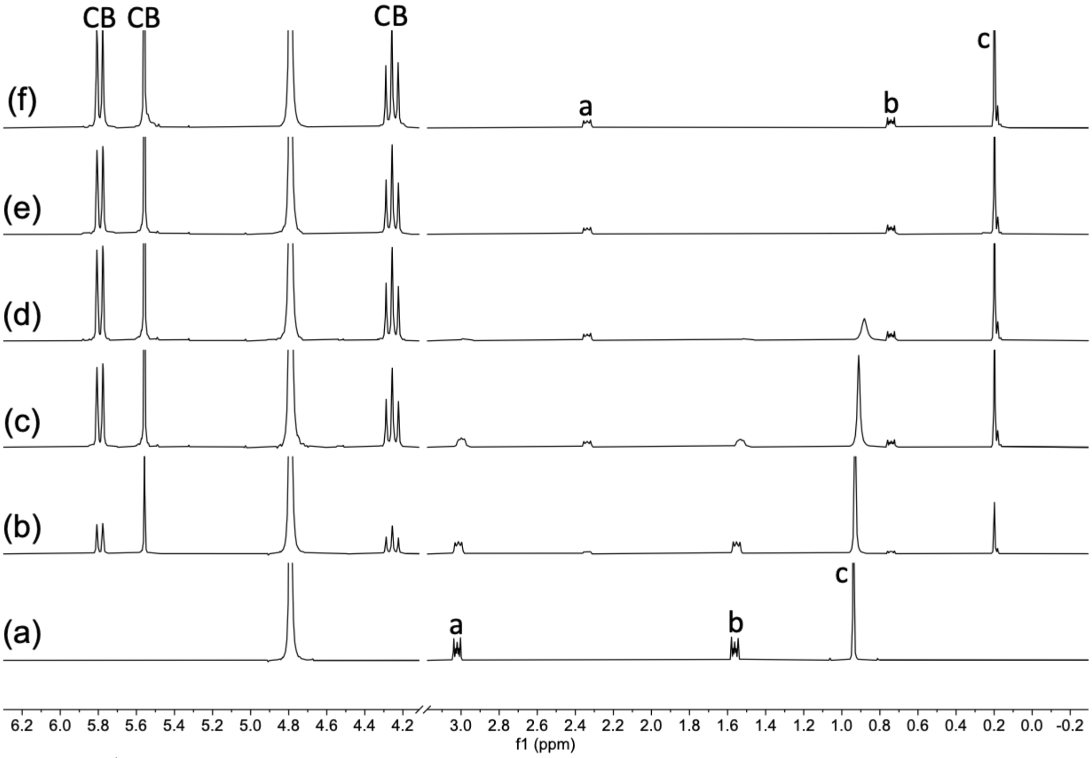

Figure 6. ^{1}H NMR spectra of guest **3** titrated with (a) 0.00, (b) 0.25, (c) 0.50, (d) 0.75, (e) 1.00, and (f) 1.50 equiv CB[7] in D_2O.

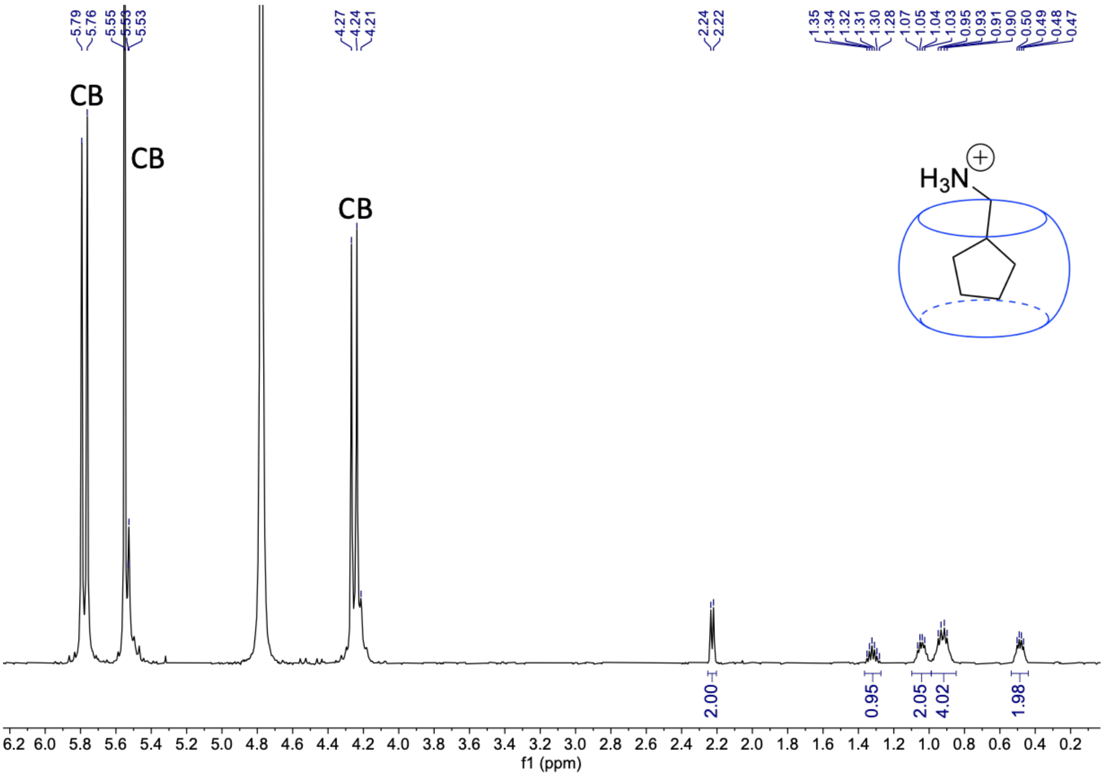

Figure 7. ^{1}H NMR spectrum of complex **4**·CB[7] in D_2O. ^{1}H NMR: δ 2.23 (d, *J* = 7.7 Hz, 2H), 1.37 – 1.27 (m, 1H), 1.10 – 0.99 (m, 2H), 0.92 (td, *J* = 7.8, 7.9 Hz, 4H), 0.48 (dd, *J* = 6.2, 6.0 Hz, 2H).

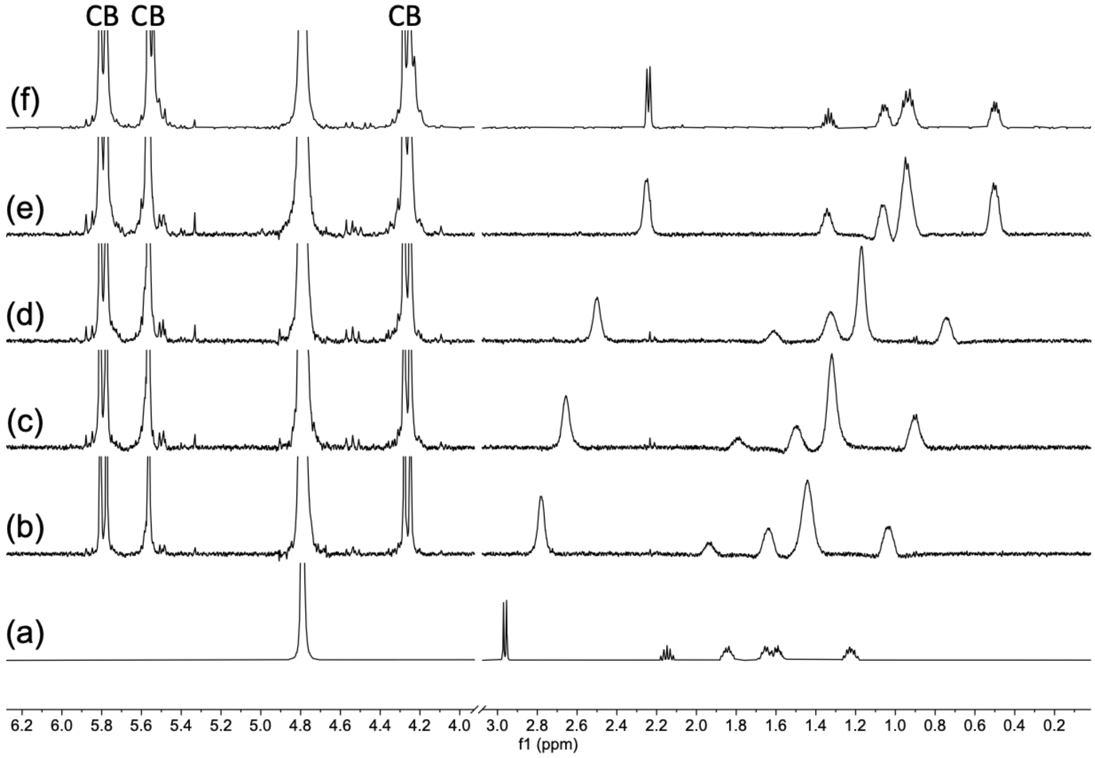

Figure 8. ^{1}H NMR spectra of guest **4** titrated with (a) 0.00, (b) 0.25, (c) 0.50, (d) 0.75, (e) 1.00, and (f) 1.50 equiv CB[7] in D_2O.

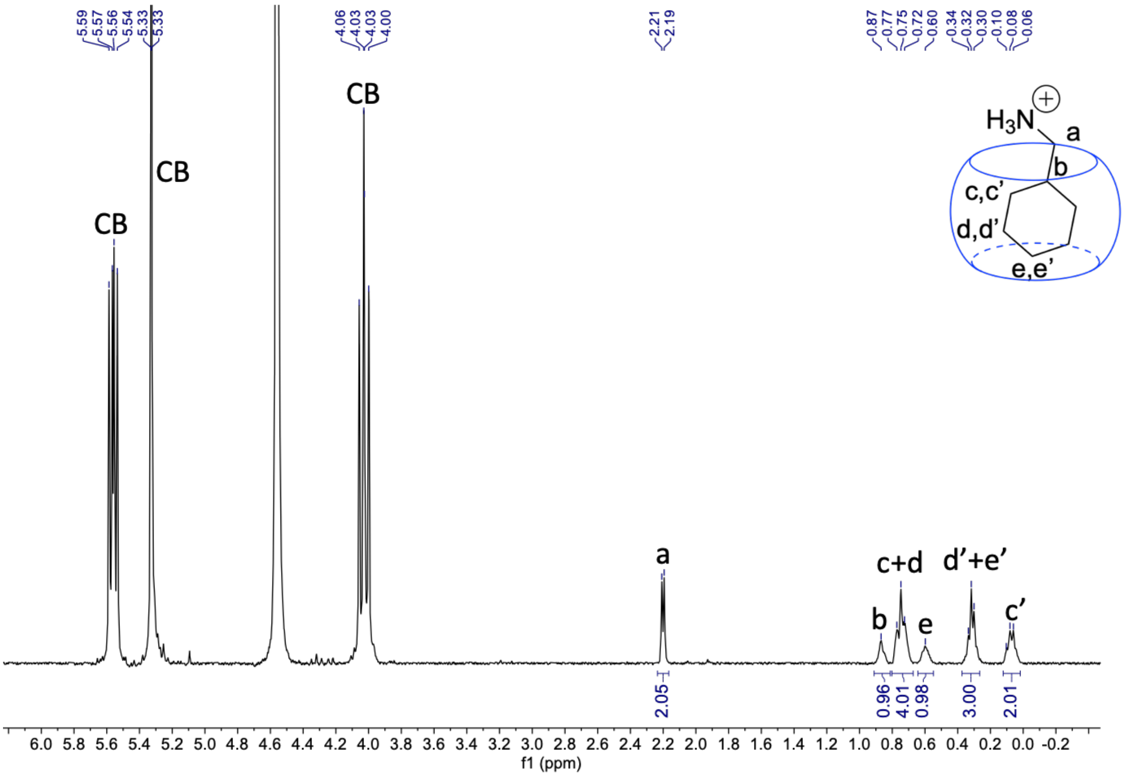

Figure 9. ^{1}H NMR spectrum of complex **5**·CB[7] in D_2O. ^{1}H NMR: δ 2.20 (d, J = 7.2 Hz, 2H, H^a), 0.87 (s, 1H, H^b), 0.75 (m, 4H, H^c+H^d), 0.60 (s, 1H, H^e), 0.32 (m, 3H, $H^{d'}$+$H^{e'}$), 0.12 – 0.08 (m, 2H, $H^{c'}$).

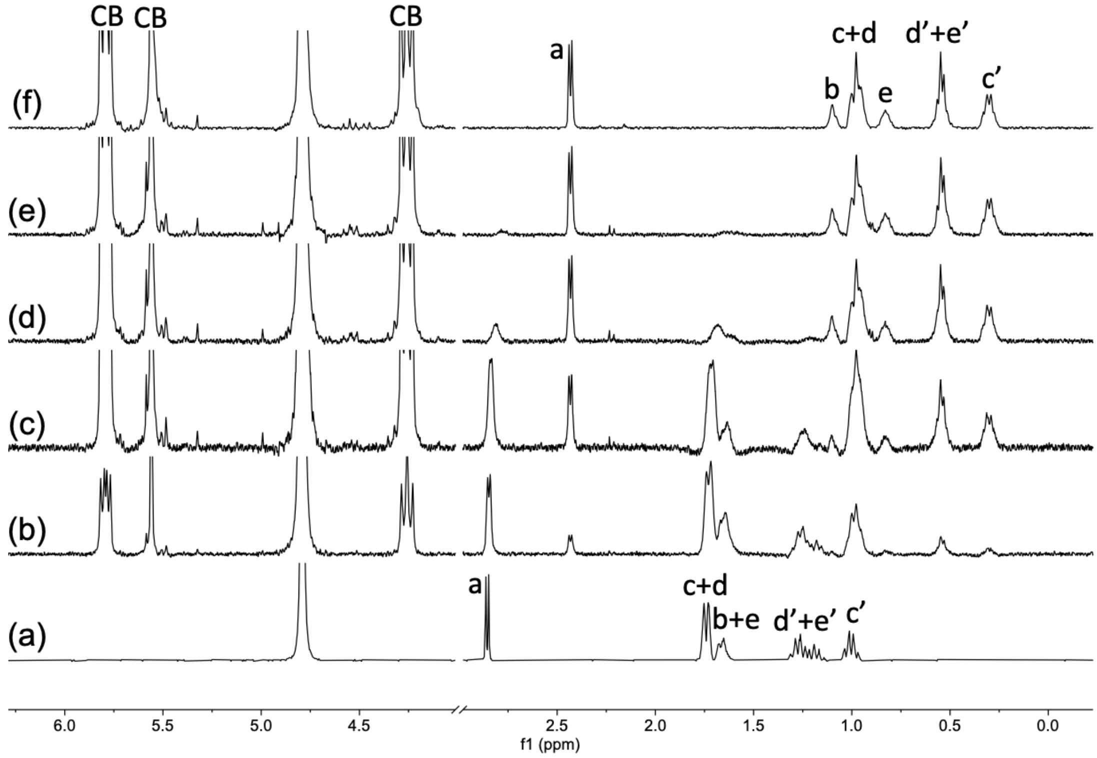

Figure 10. ^{1}H NMR spectra of guest **5** titrated with (a) 0.00, (b) 0.25, (c) 0.50, (d) 0.75, (e) 1.00, and (f) 1.50 equiv CB[7] in D_2O.

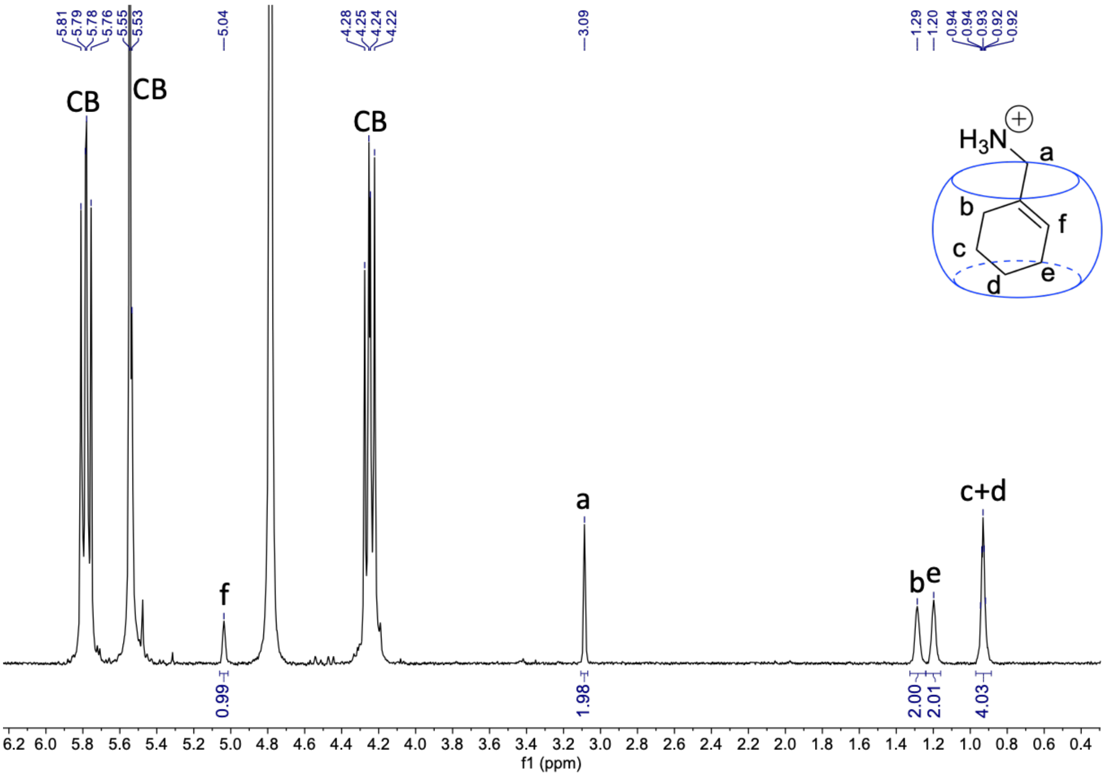

Figure 11. ^{1}H NMR spectrum of complex **6**·CB[7] in D_2O. ^{1}H NMR: δ 5.04 (s, 1H, H^f), 3.09 (s, 2H, H^a), 1.29 (s, 2H, H^b), 1.20 (s, 2H, H^e), 0.97 – 0.89 (m, 4H, H^c+H^d).

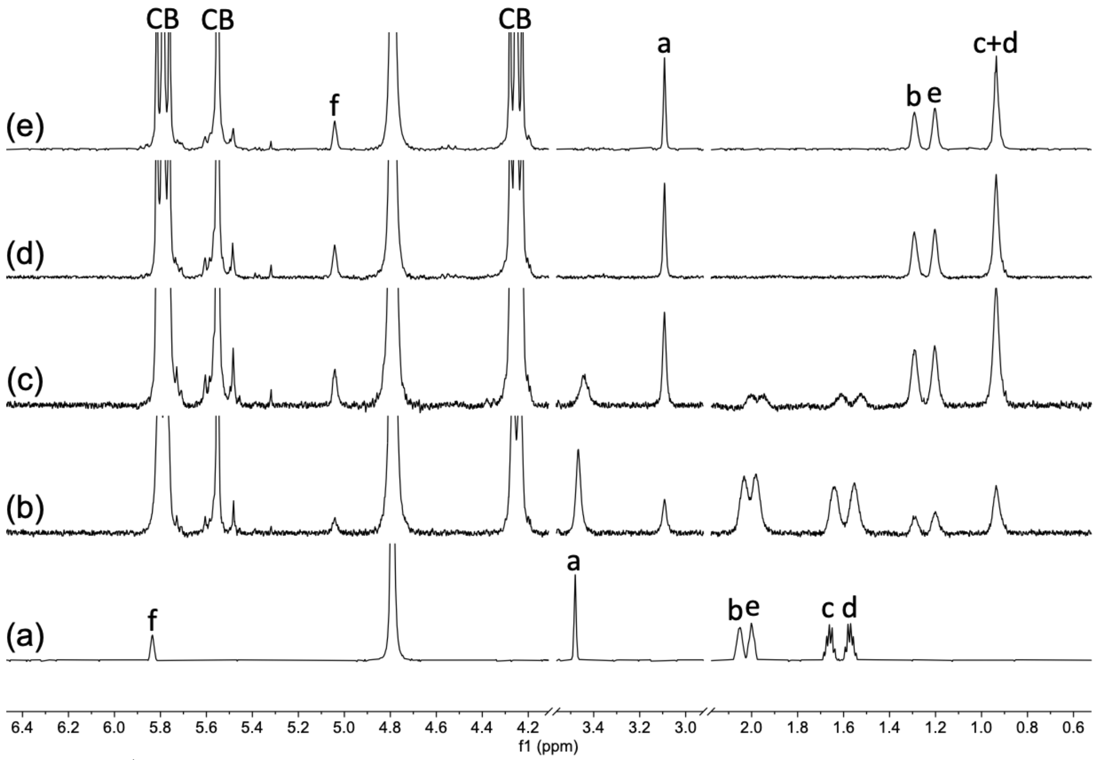

Figure 12. ^{1}H NMR spectra of guest **6** titrated with (a) 0.00, (b) 0.25, (c) 0.60, (d) 1.00, and (e) 1.50 equiv CB[7] in D_2O.

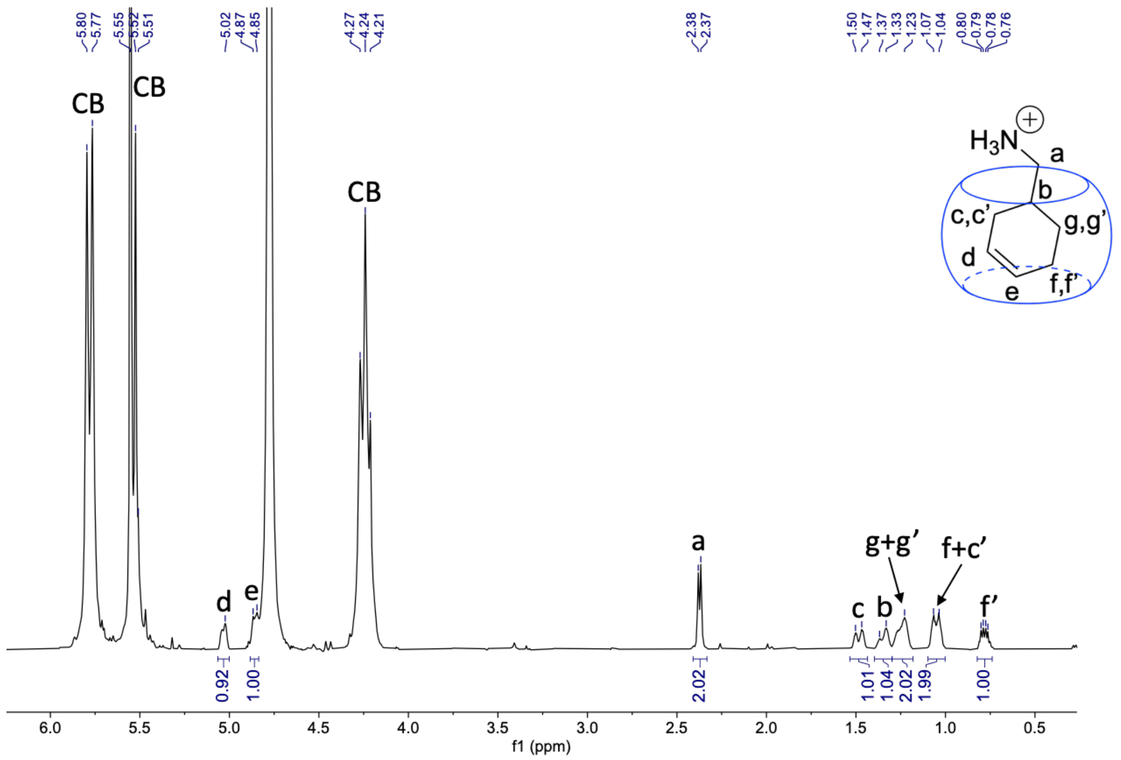

Figure 13. ^{1}H NMR spectrum of complex **7**·CB[7] in D_2O. ^{1}H NMR: δ 5.06 – 5.00 (m, 1H, H^{d}), 4.88 – 4.84 (m, 1H, H^{e}), 2.37 (d, J = 7.2 Hz, 2H, H^{a}), 1.53 – 1.44 (m, 1H, H^{c}), 1.30 – 140 (m, 1H, H^{b}), 1.30 – 1.18 (m, 2H, H^{g}+$H^{g'}$), 1.10 – 1.00 (m, 2H, H^{f}+$H^{c'}$), 0.78 (dd, J = 12.9, 6.0 Hz, 1H, $H^{f'}$).

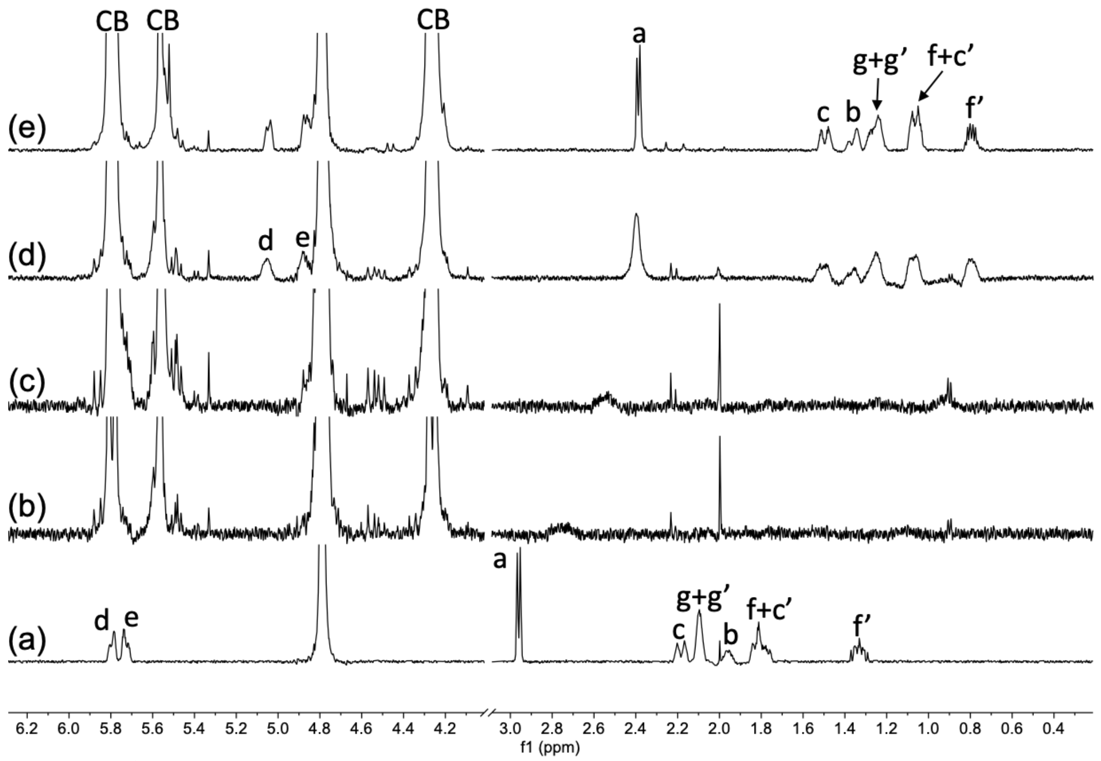

Figure 14. ^{1}H NMR spectra of guest **7** titrated with (a) 0.00, (b) 0.35, (c) 0.75, (d) 1.00, and (e) 1.50 equiv CB[7] in D_2O.

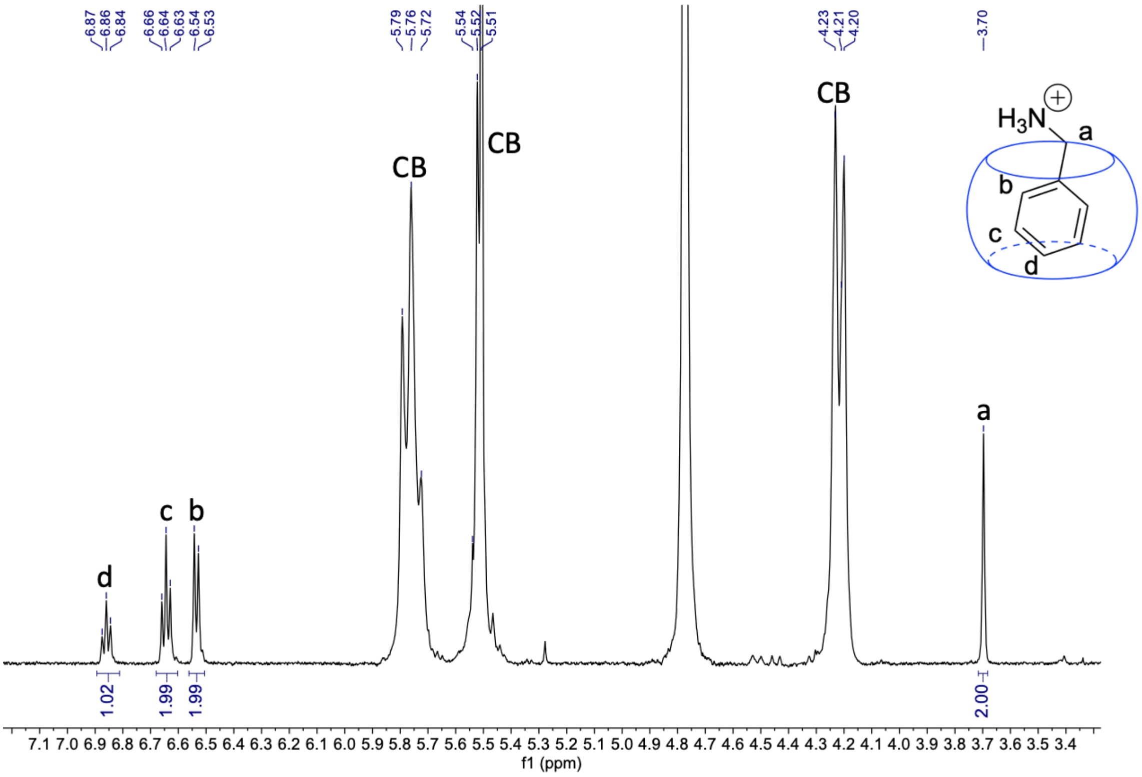

Figure 15. ^{1}H NMR spectrum of complex **8**·CB[7] in D_2O. ^{1}H NMR: δ 6.86 (t, *J* = 7.5 Hz, 1H, H^d), 6.64 (t, *J* = 7.7 Hz, 2H, H^c), 6.54 (d, *J* = 7.5 Hz, 2H, H^b), 3.70 (s, 2H, H^a).

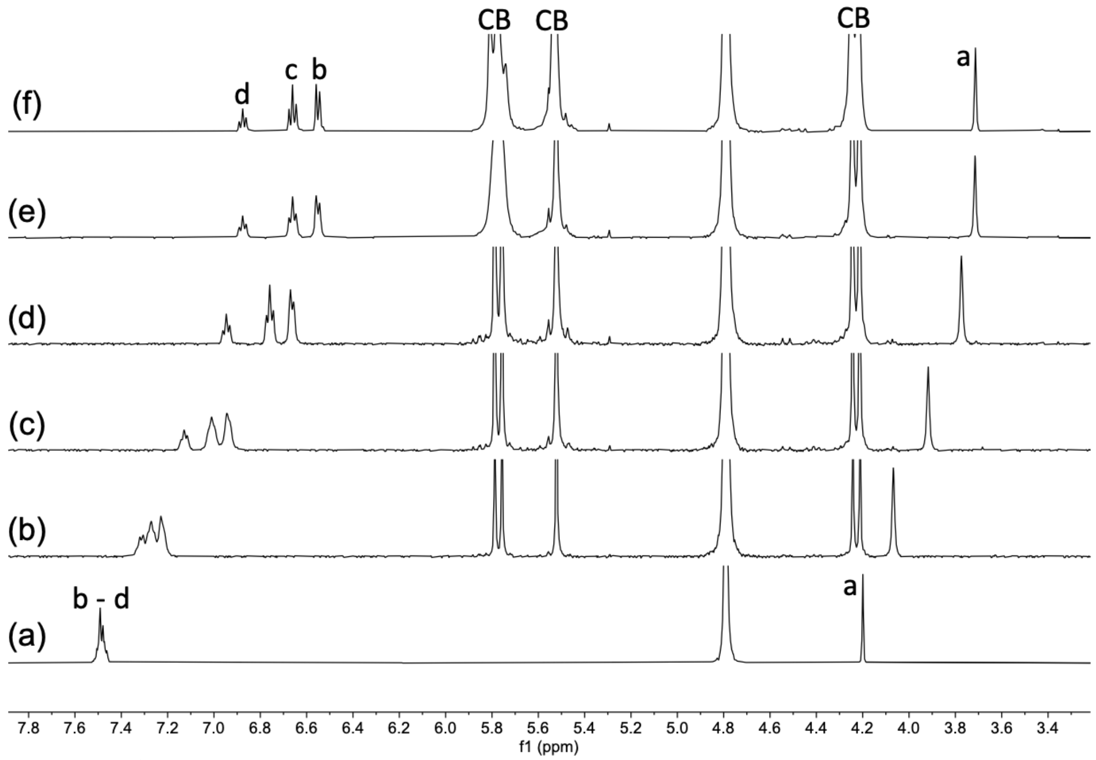

Figure 16. ^{1}H NMR spectra of guest **8** titrated with (a) 0.00, (b) 0.25, (c) 0.50, (d) 0.75, (e) 1.00, and (f) 1.50 equiv CB[7] in D_2O.

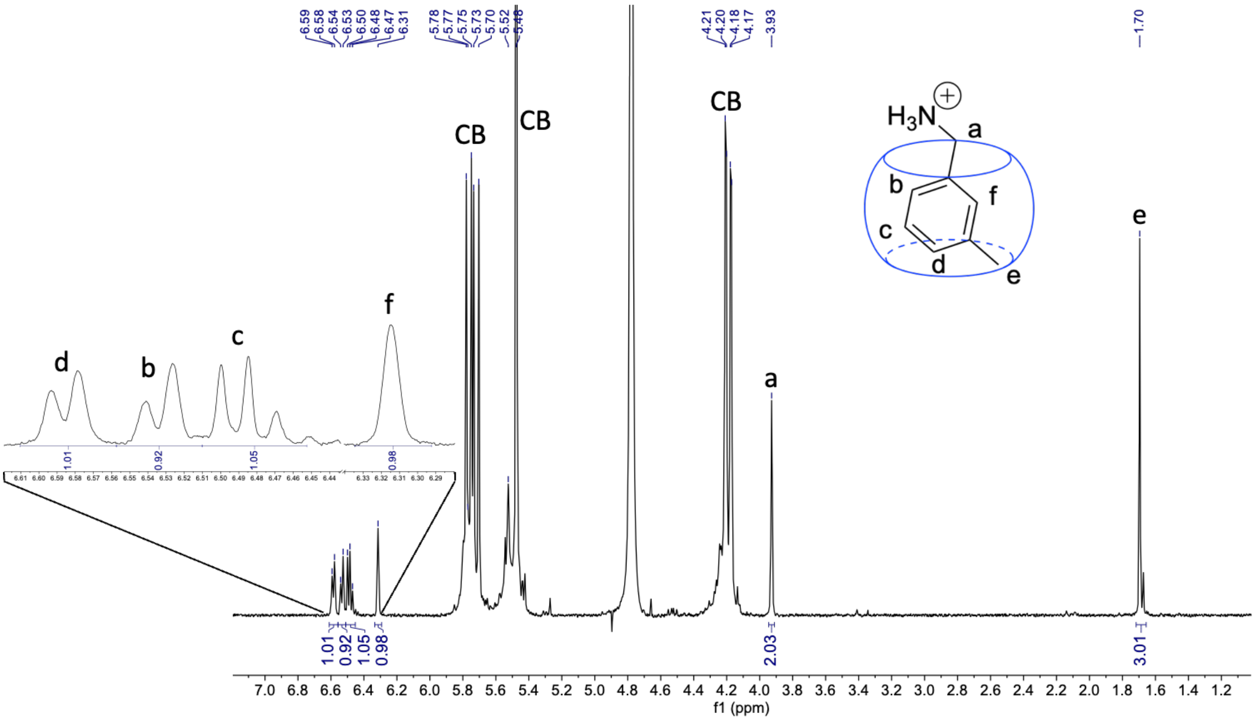

Figure 17. ^{1}H NMR spectrum of complex **9**·CB[7] in D_2O. ^{1}H NMR: δ 6.59 (d, J = 7.5 Hz, 1H, H^d), 6.53 (d, J = 7.6 Hz, 1H, H^b), 6.51 – 6.45 (m, 1H, H^c), 6.31 (s, 1H, H^f), 3.93 (s, 2H, H^a), 1.70 (s, 3H, H^e).

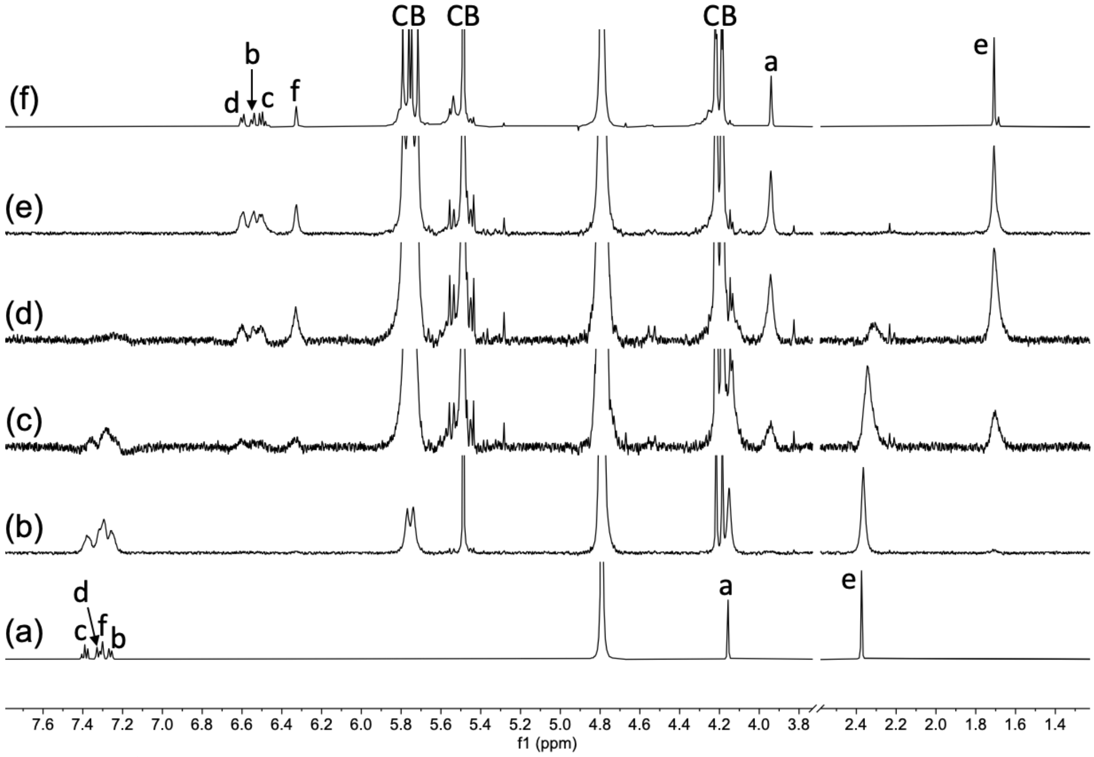

Figure 18. ^{1}H NMR spectra of guest **9** titrated with (a) 0.00, (b) 0.25, (c) 0.50, (d) 0.75, (e) 1.00, and (f) 1.50 equiv CB[7] in D_2O.

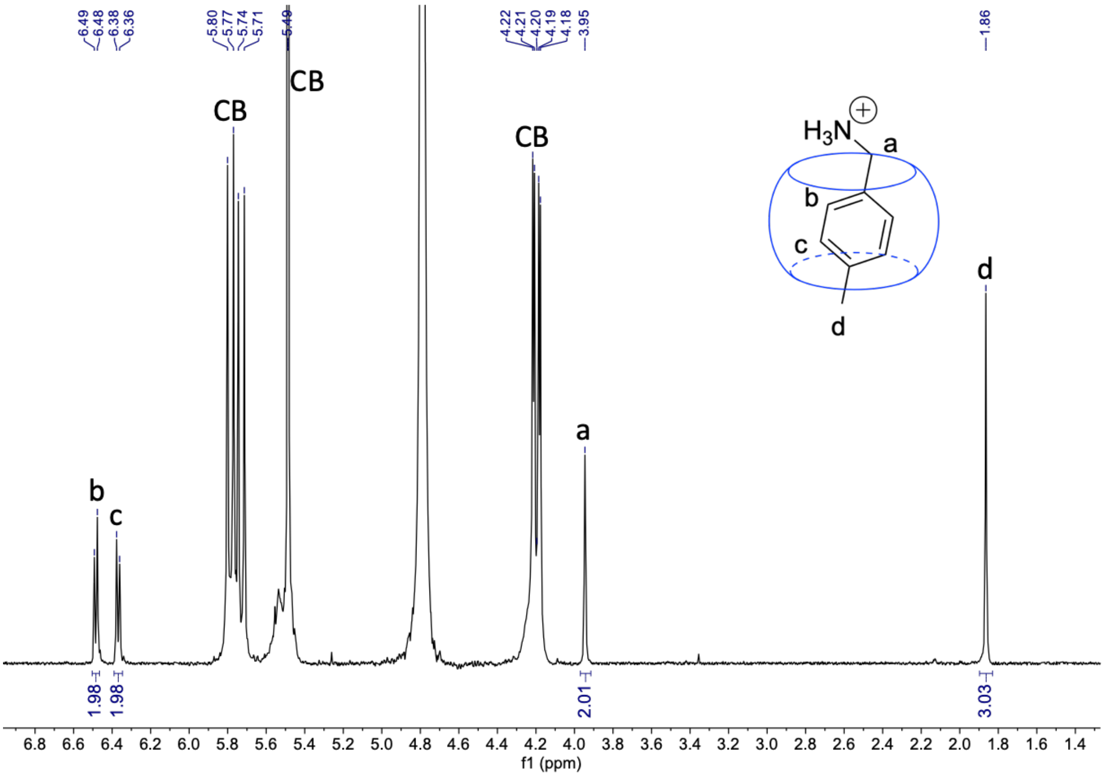

Figure 19. ^{1}H NMR spectrum of complex **10**·CB[7] in D_2O. ^{1}H NMR: δ 6.48 (d, J = 7.9 Hz, 2H, H^b), 6.37 (d, J = 7.9 Hz, 2H, H^c), 3.95 (s, 2H, H^a), 1.86 (s, 3H, H^d).

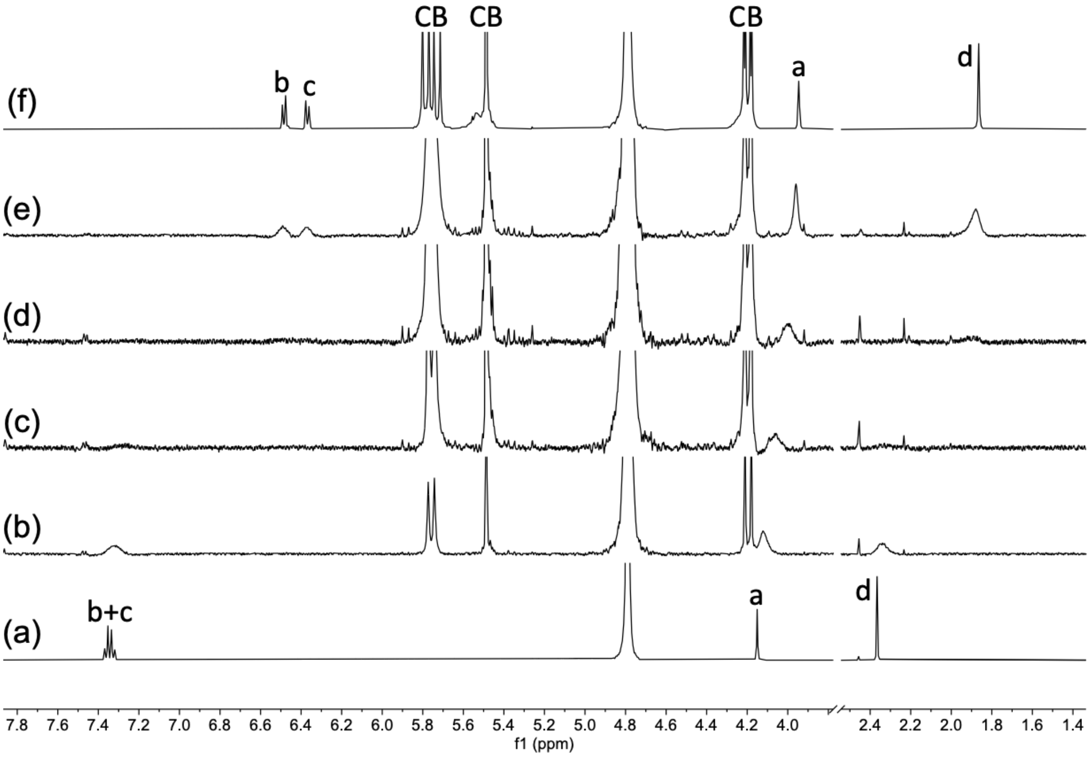

Figure 20. ^{1}H NMR spectra of guest **10** titrated with (a) 0.00, (b) 0.25, (c) 0.50, (d) 0.75, (e) 1.00, and (f) 1.50 equiv CB[7] in D_2O.

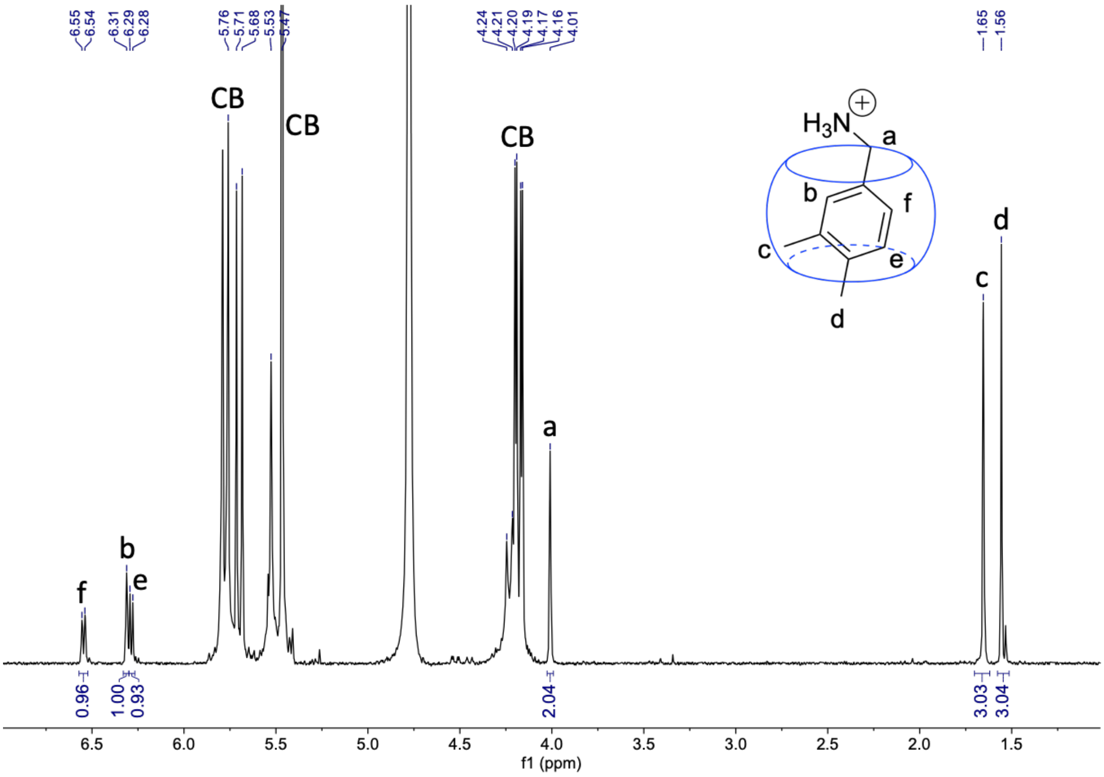

Figure 21. ^{1}H NMR spectrum of complex **11**·CB[7] in D_2O. ^{1}H NMR: δ 6.55 (d, J = 8.0 Hz, 1H, H^f), 6.31 (s, 1H, H^b), 6.29 (d, J = 7.8 Hz, 1H, H^e), 4.01 (s, 2H, H^a), 1.65 (s, 3H, H^c), 1.56 (s, 3H, H^d).

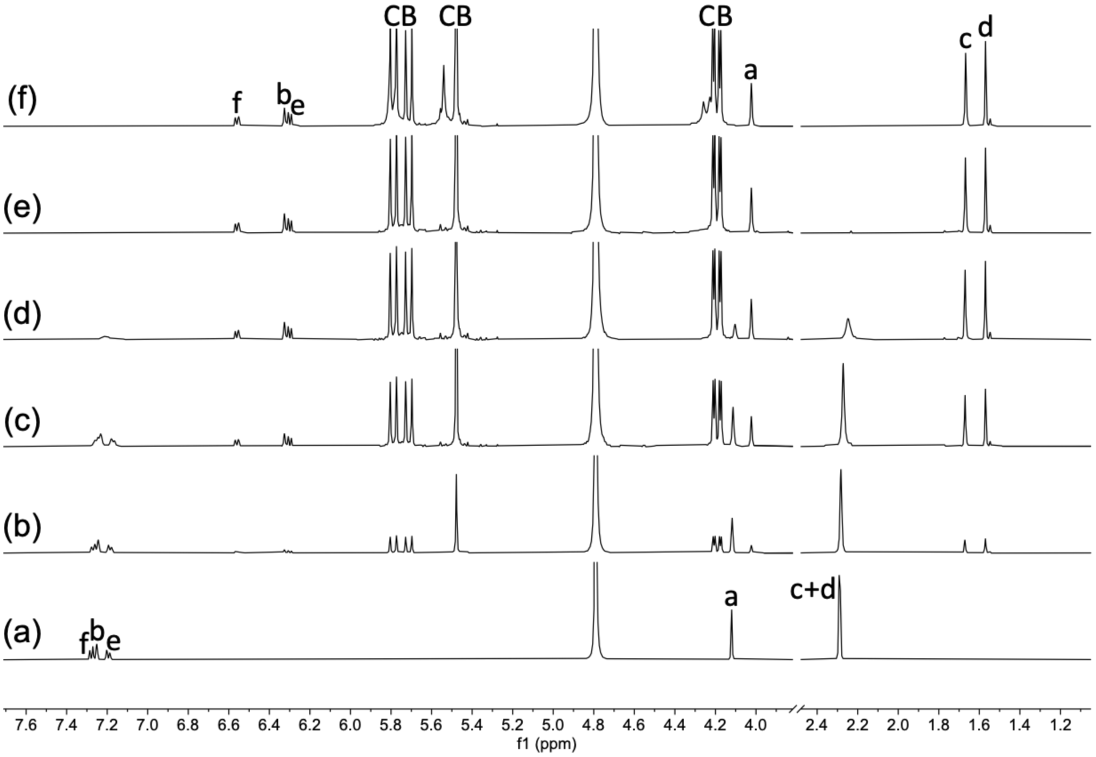

Figure 22. ^{1}H NMR spectra of guest **11** titrated with (a) 0.00, (b) 0.25, (c) 0.50, (d) 0.75, (e) 1.00, and (f) 1.50 equiv CB[7] in D_2O.

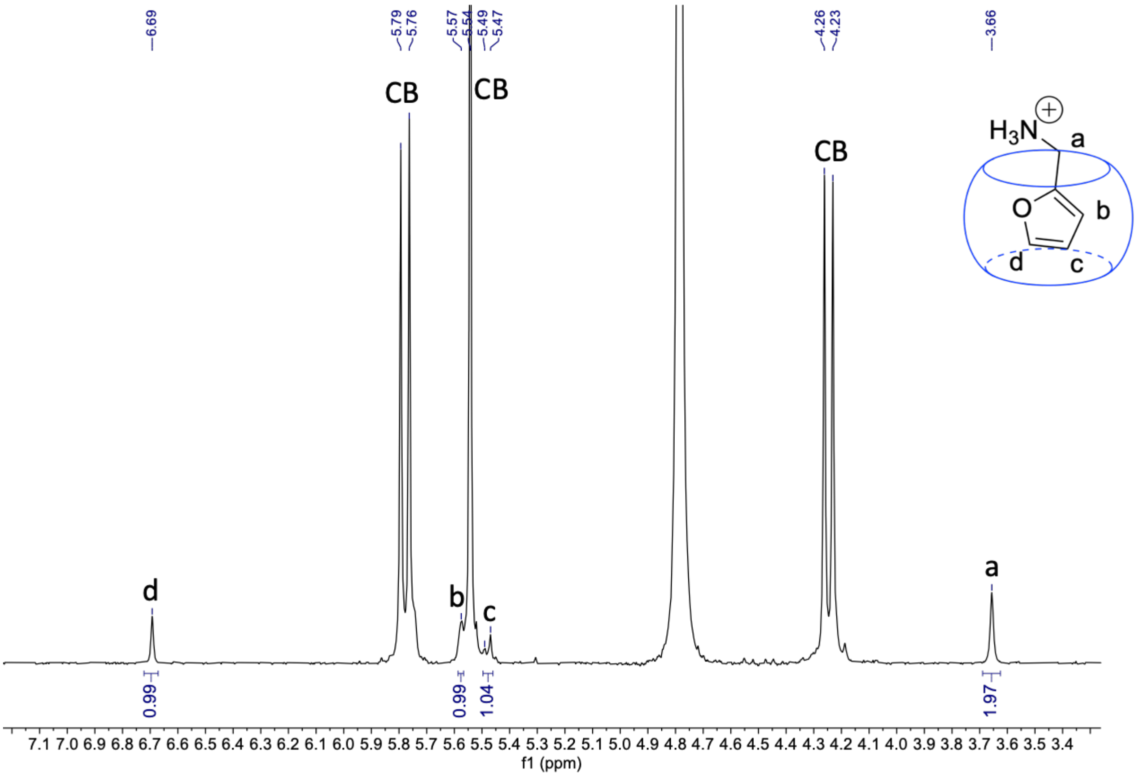

Figure 23. ^{1}H NMR spectrum of complex **12**·CB[7] in D_2O. ^{1}H NMR: δ 6.69 (s, 1H, H^d), 5.59 – 5.57 (m, 1H, H^b), 5.50 – 5.46 (m, 1H, H^c), 3.66 (s, 2H, H^a).

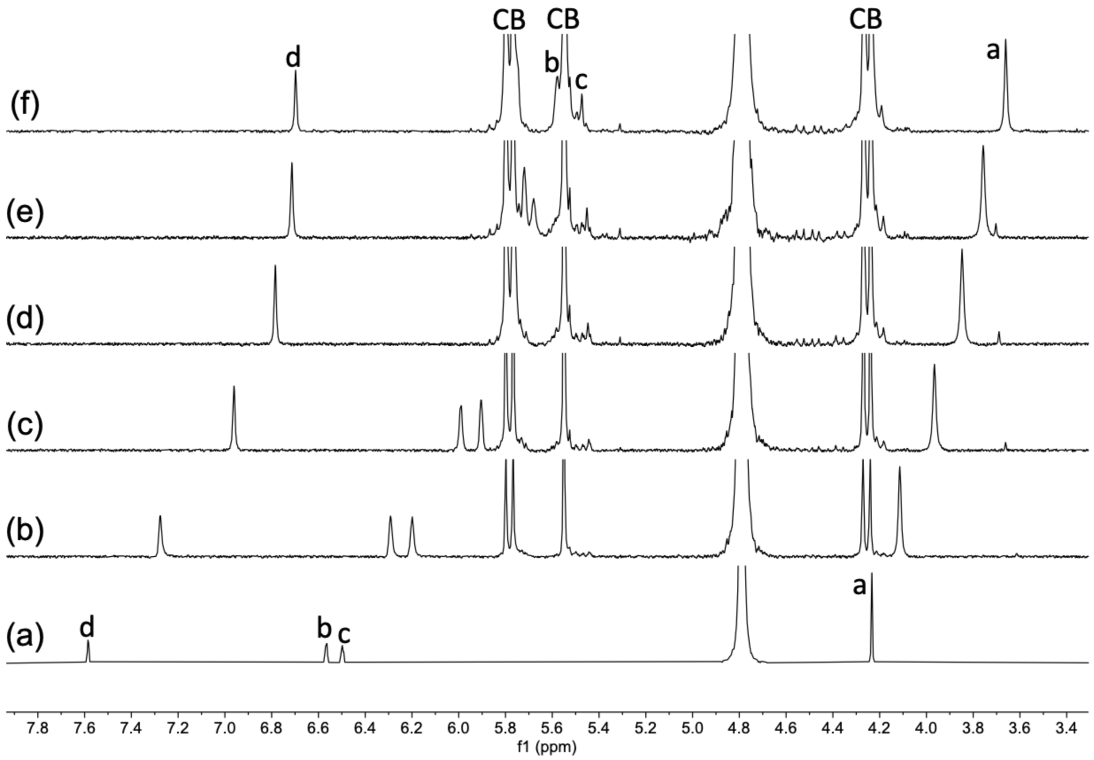

Figure 24. ^{1}H NMR spectra of guest **12** titrated with (a) 0.00, (b) 0.25, (c) 0.50, (d) 0.75, (e) 1.00, and (f) 1.50 equiv CB[7] in D_2O.

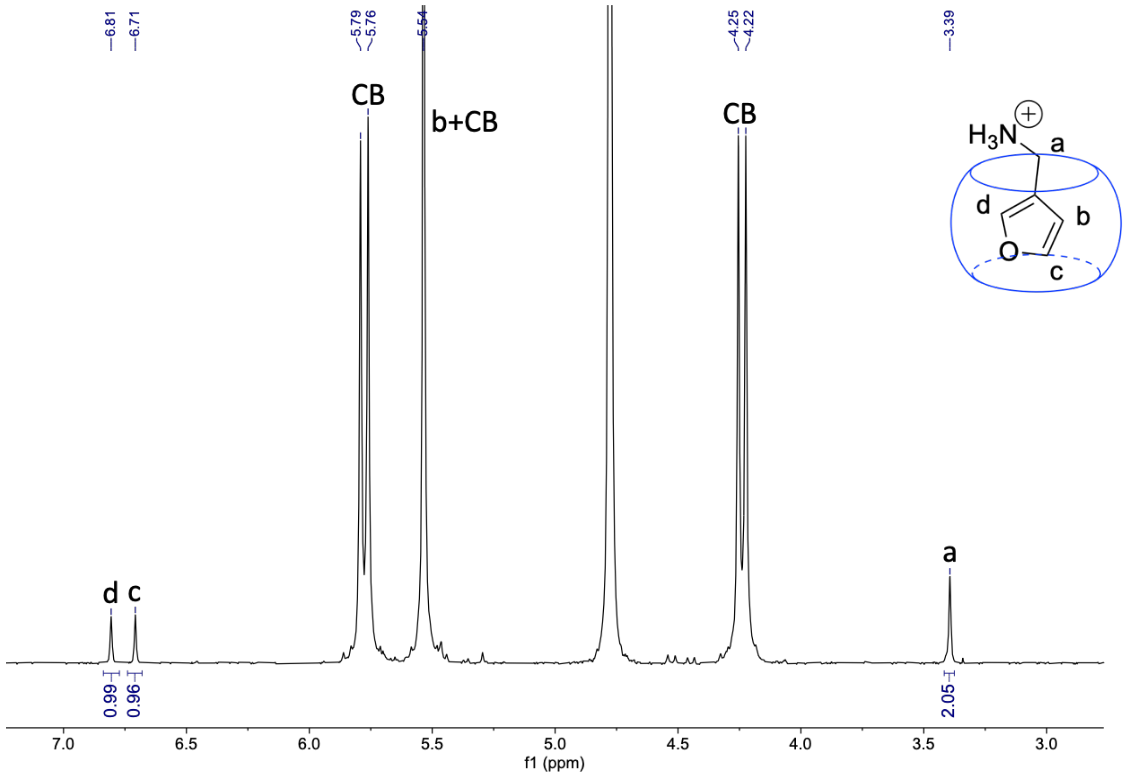

Figure 25. ^{1}H NMR spectrum of complex **13**·CB[7] in D_2O. ^{1}H NMR: δ 6.81 (s, 1H, H^d), 6.71 (s, 1H, H^c), 3.39 (s, 2H, H^a).

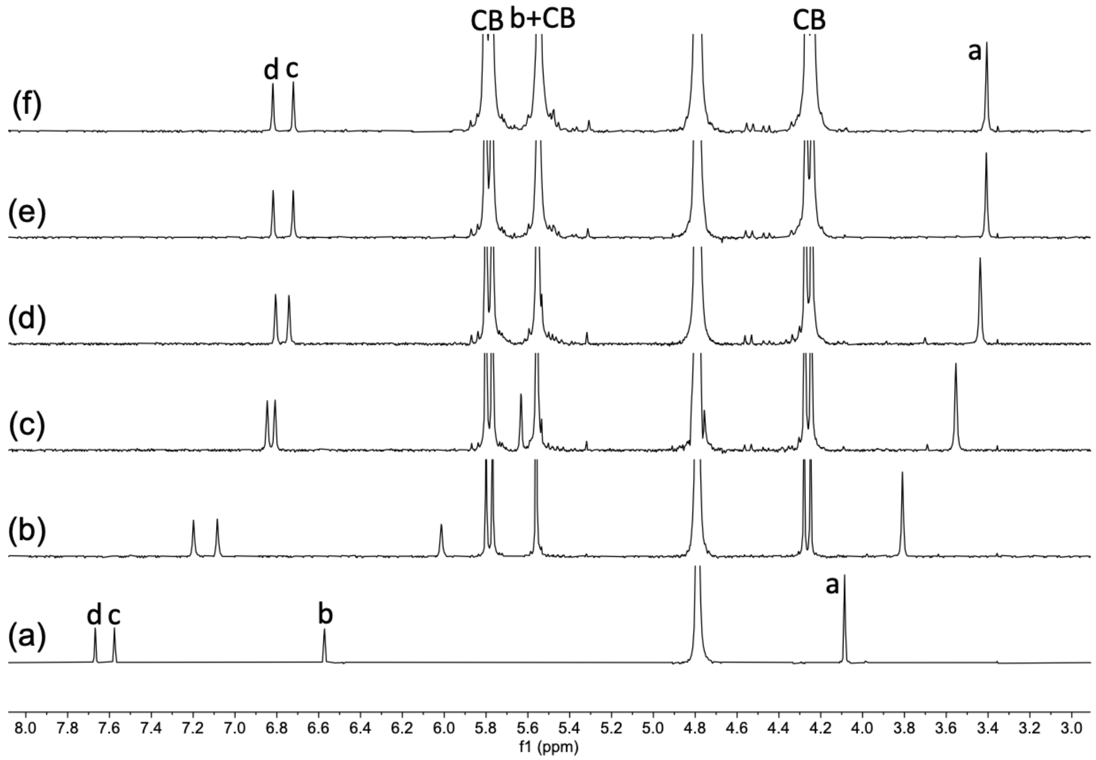

Figure 26. ^{1}H NMR spectra of guest **13** titrated with (a) 0.00, (b) 0.25, (c) 0.50, (d) 0.75, (e) 1.00, and (f) 1.50 equiv CB[7] in D_2O.

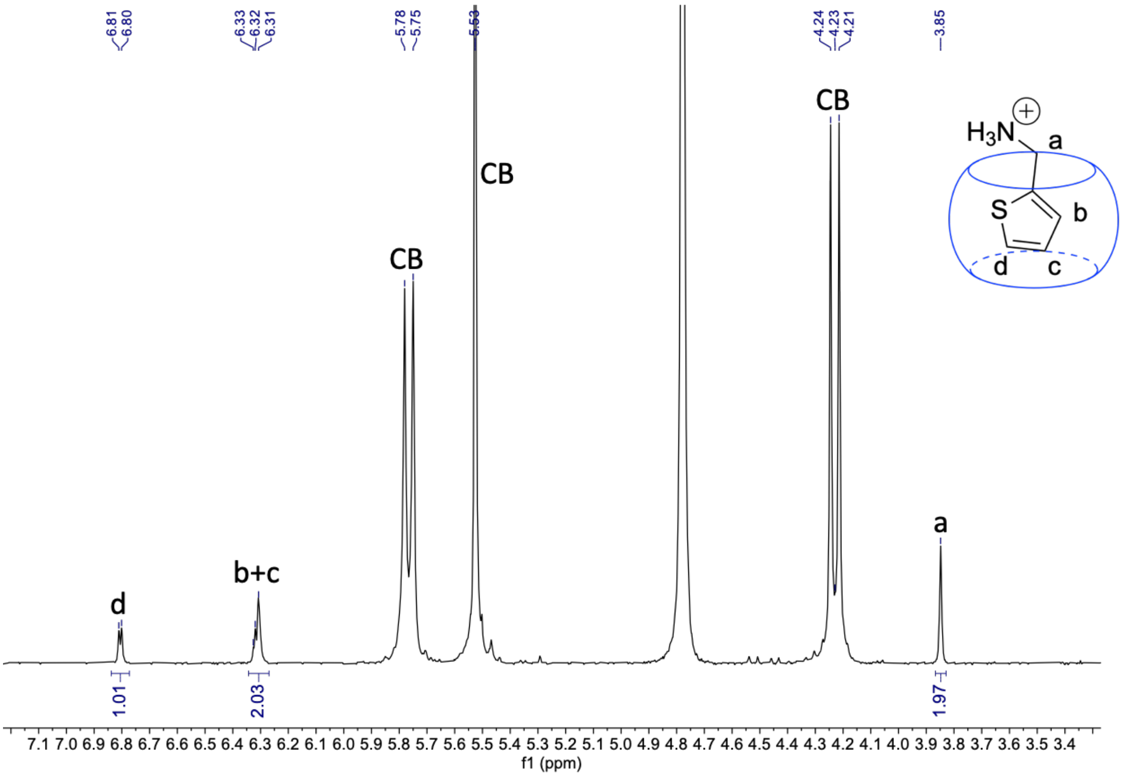

Figure 27. ^{1}H NMR spectrum of complex **14**·CB[7] in D_2O. ^{1}H NMR: δ 6.81 (d, J = 5.0 Hz, 1H, H^d), 6.34 – 6.27 (m, 2H, H^b+H^c), 3.85 (s, 2H, H^a).

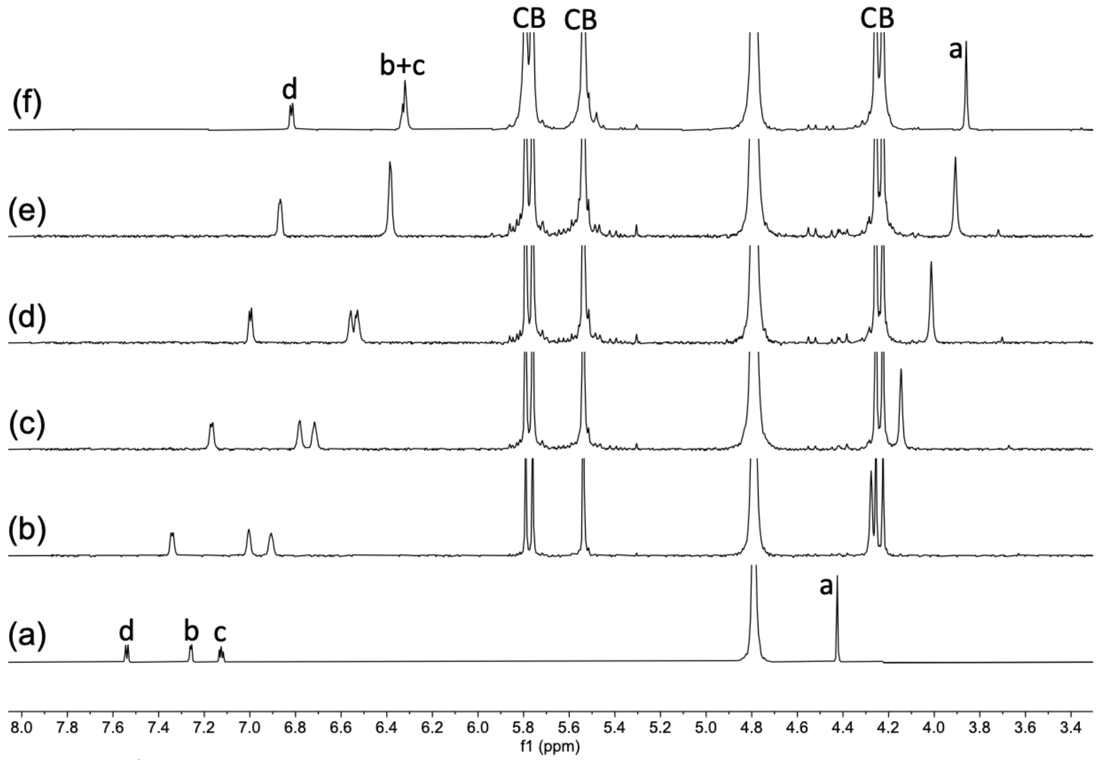

Figure 28. ^{1}H NMR spectra of guest **14** titrated with (a) 0.00, (b) 0.25, (c) 0.50, (d) 0.75, (e) 1.00, and (f) 1.50 equiv CB[7] in D_2O.

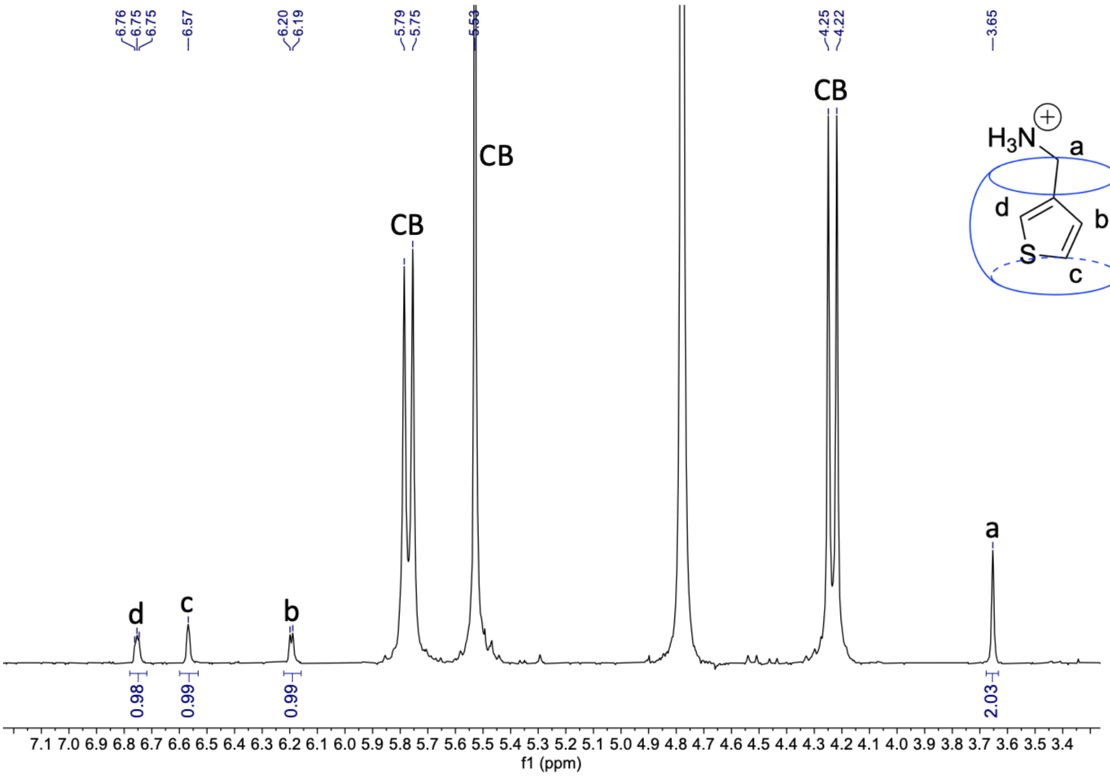

Figure 29. ^1H NMR spectrum of complex **15**·CB[7] in D_2O. ^1H NMR: δ 6.78 – 6.72 (sym. m, 1H, H^d), 6.57 (s, 1H, H^c), 6.19 (d, J = 5.0 Hz, 1H, H^b), 3.65 (s, 2H, H^a).

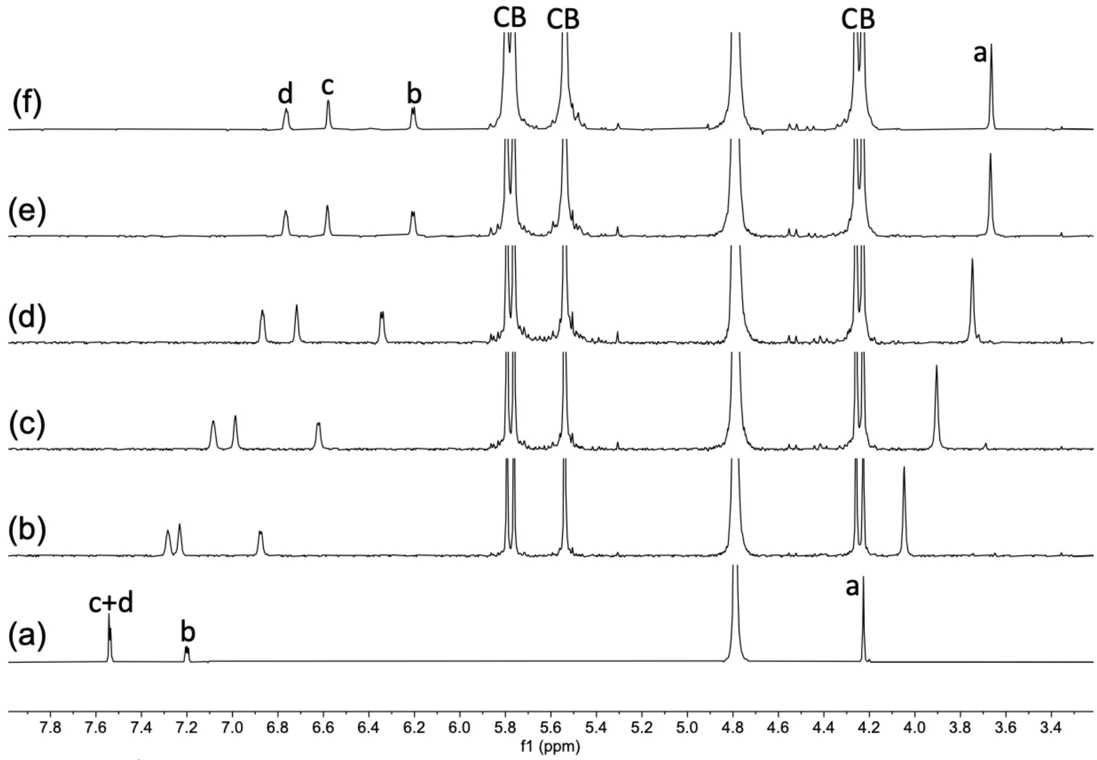

Figure 30. ^1H NMR spectra of guest **15** titrated with (a) 0.00, (b) 0.25, (c) 0.50, (d) 0.75, (e) 1.00, and (f) 1.50 equiv CB[7] in D_2O.

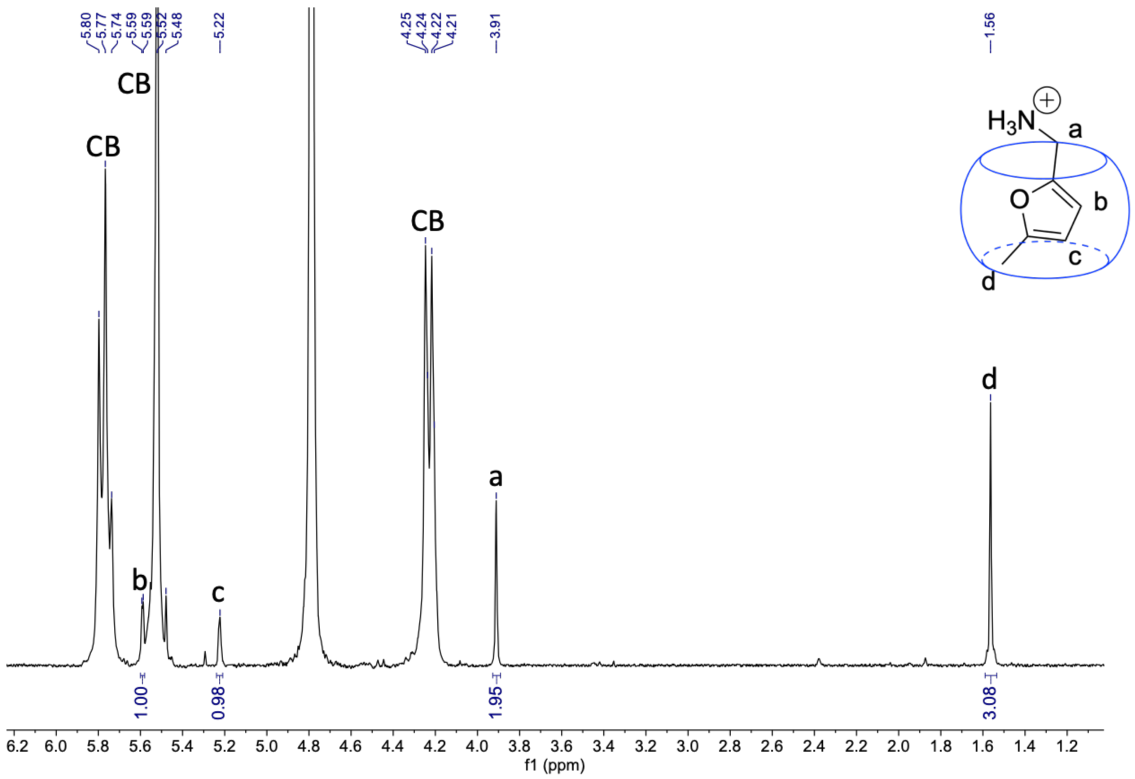

Figure 31. ^{1}H NMR spectrum of complex **16**·CB[7] in D_2O. ^{1}H NMR: δ 5.58 – 5.60 (m, 1H, H^b), 5.24 – 5.21 (m, 1H, H^c), 3.91 (s, 2H, H^a), 1.57 (s, 3H, H^d).

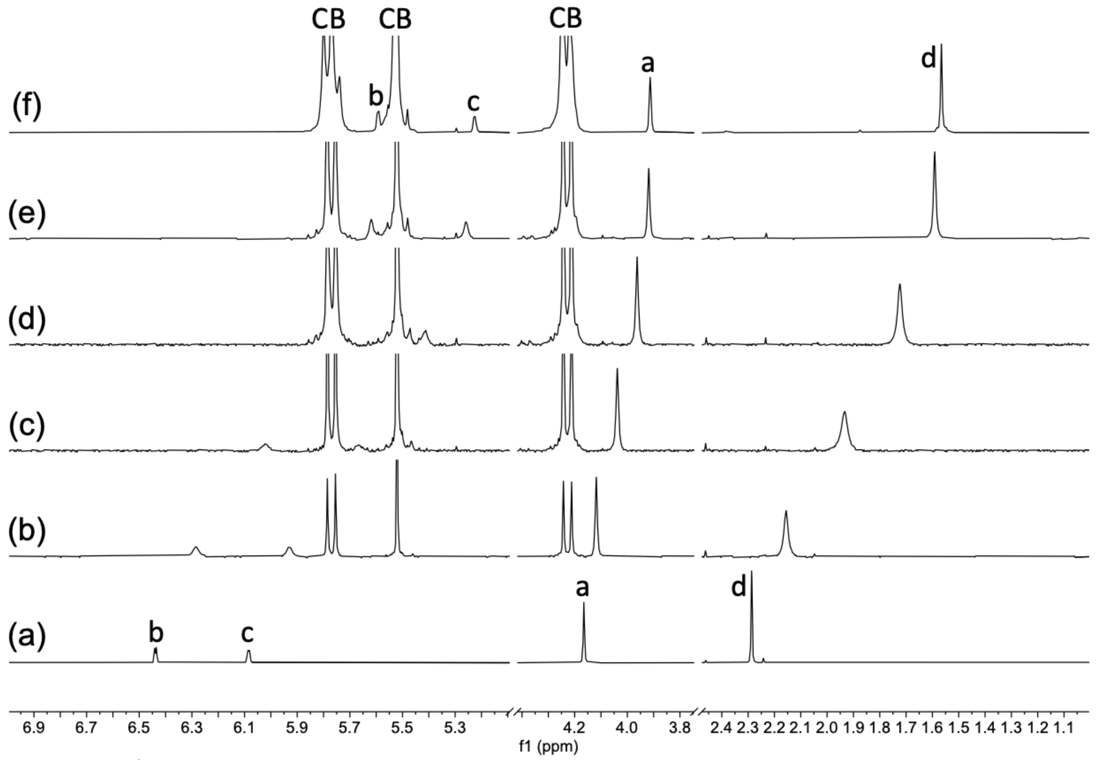

Figure 32. ^{1}H NMR spectra of guest **16** titrated with (a) 0.00, (b) 0.25, (c) 0.50, (d) 0.75, (e) 1.00, and (f) 1.50 equiv CB[7] in D_2O.

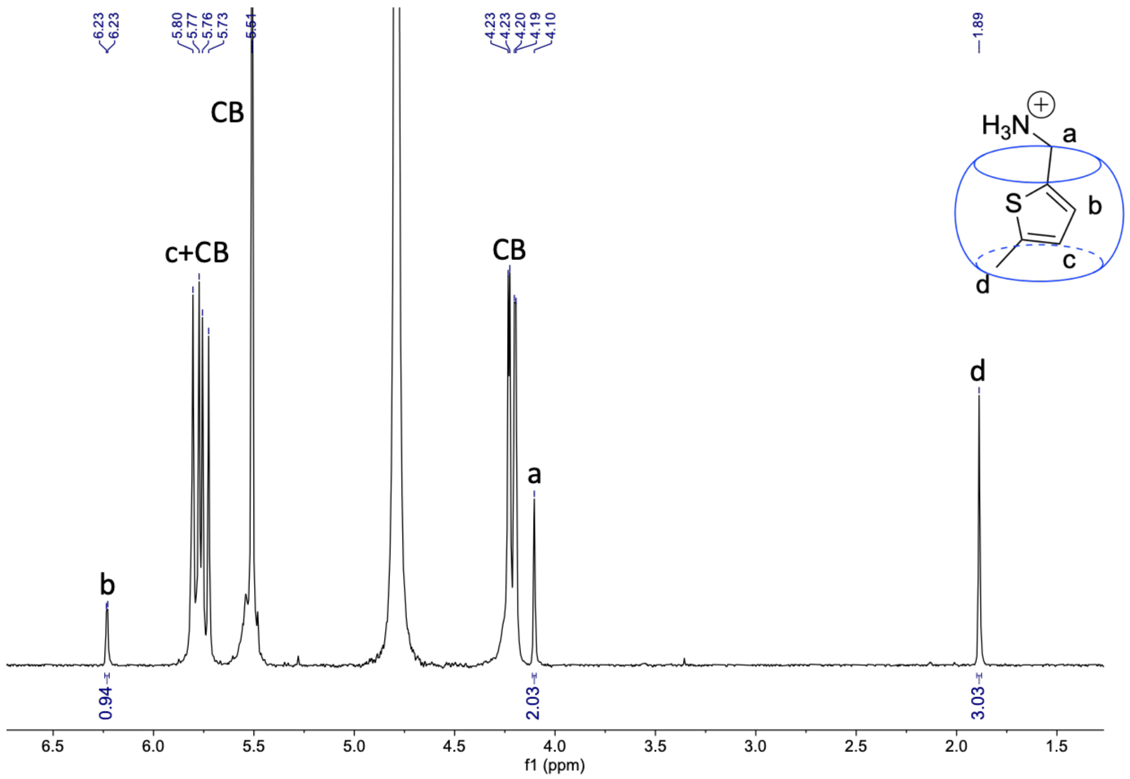

Figure 33. ^{1}H NMR spectrum of complex **17**·CB[7] in D_2O. ^{1}H NMR: δ 6.23 (d, *J* = 3.5 Hz, 1H, H^b), 4.10 (s, 2H, H^a), 1.89 (s, 3H, H^d).

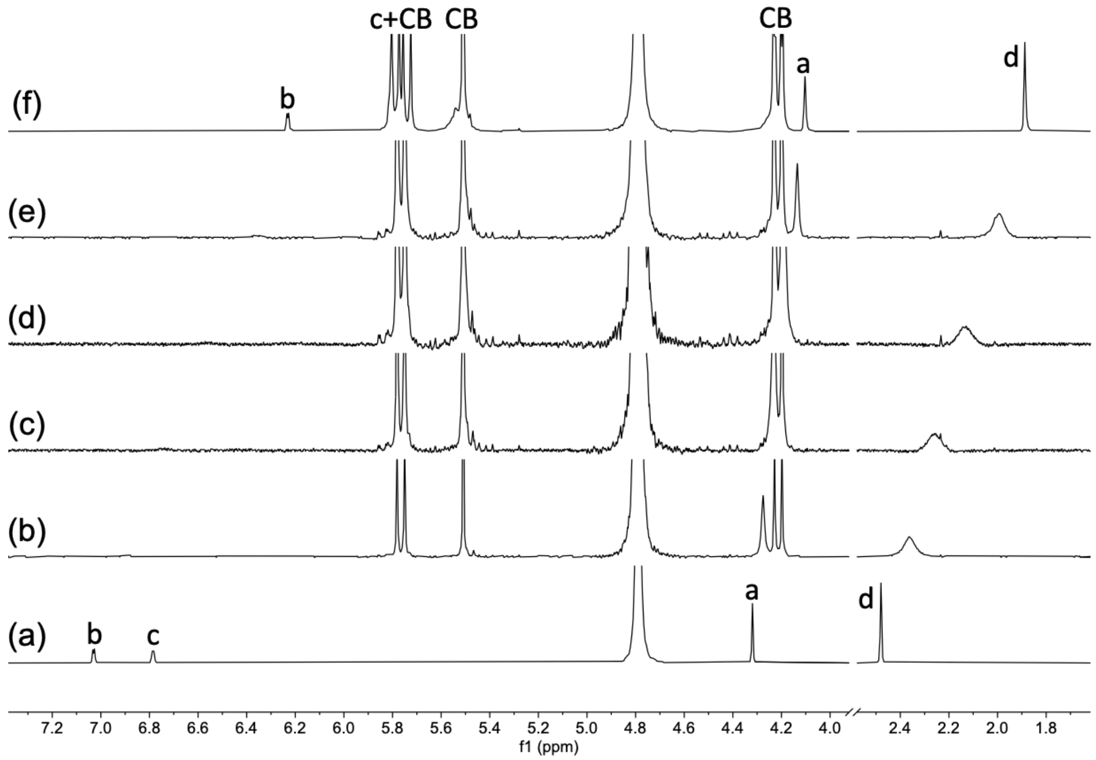

Figure 34. ^{1}H NMR spectra of guest **17** titrated with (a) 0.00, (b) 0.20, (c) 0.40, (d) 0.60, (e) 0.80, and (f) 1.00 equiv CB[7] in D_2O..

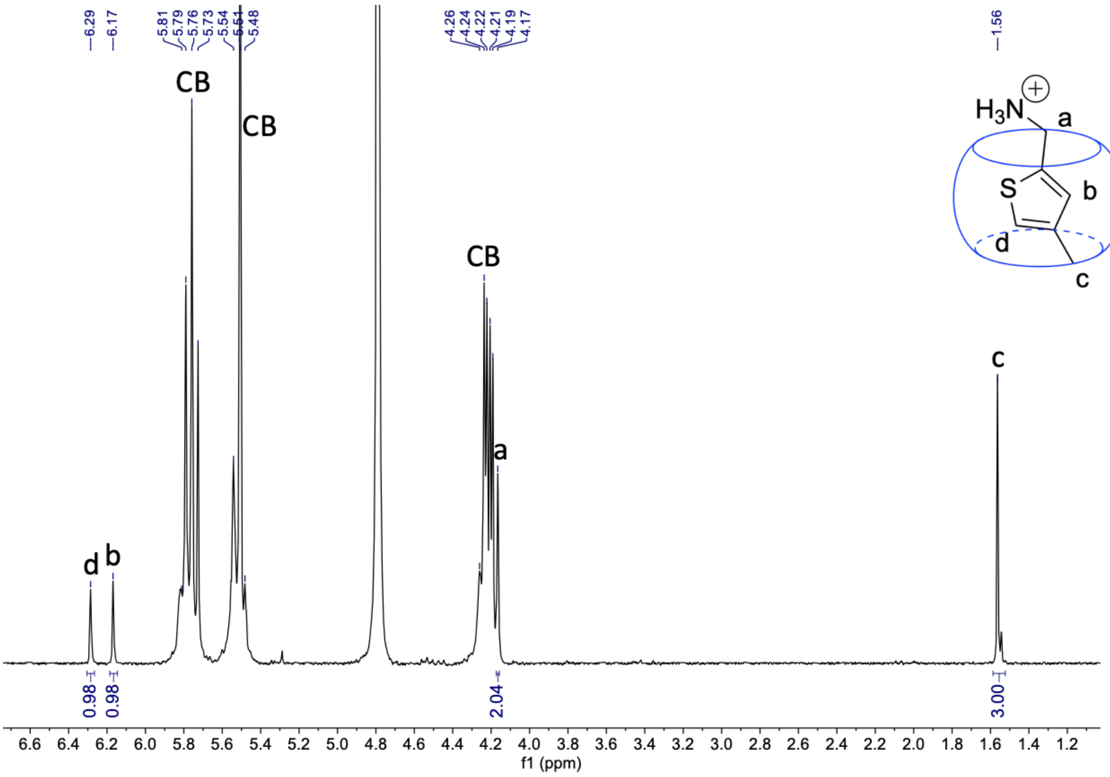

Figure 35. ^{1}H NMR spectrum of complex **18**·CB[7] in D_2O. ^{1}H NMR: δ 6.29 (s, 1H, H^d), 6.17 (s, 1H, H^b), 4.17 (s, 2H, H^a), 1.56 (s, 3H, H^c).

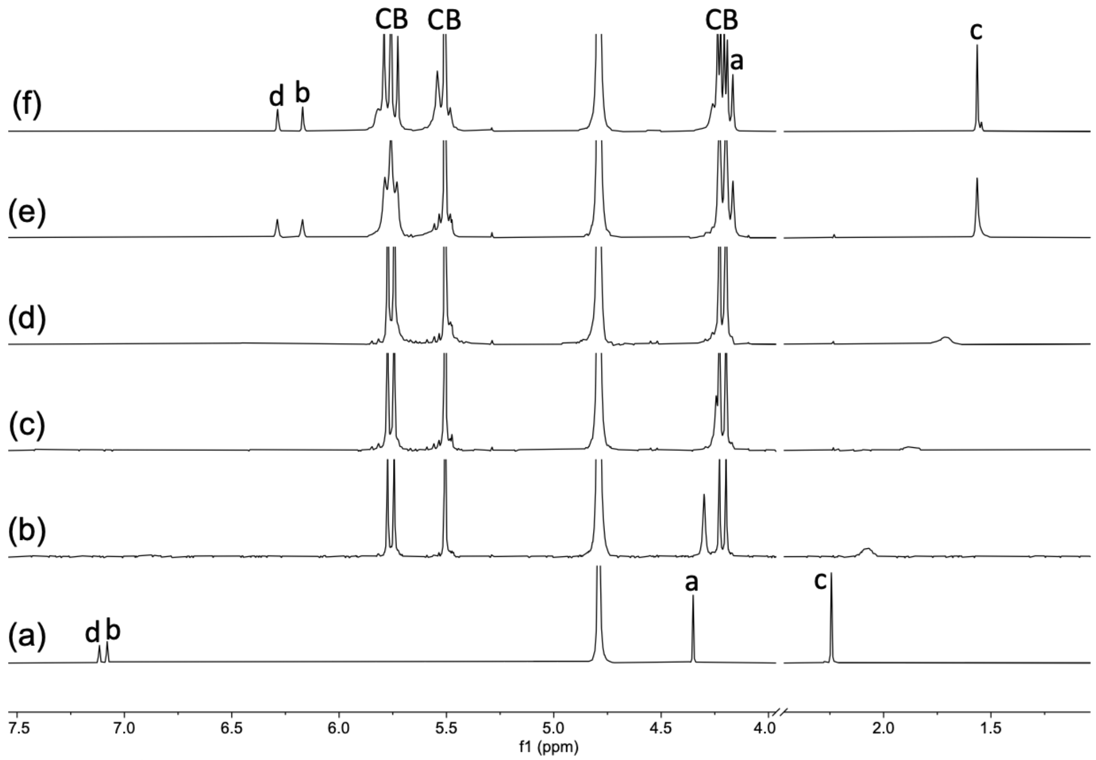

Figure 36. ^{1}H NMR spectra of guest **18** titrated with (a) 0.00, (b) 0.25, (c) 0.50, (d) 0.75, (e) 1.00, and (f) 1.50 equiv CB[7] in D_2O.

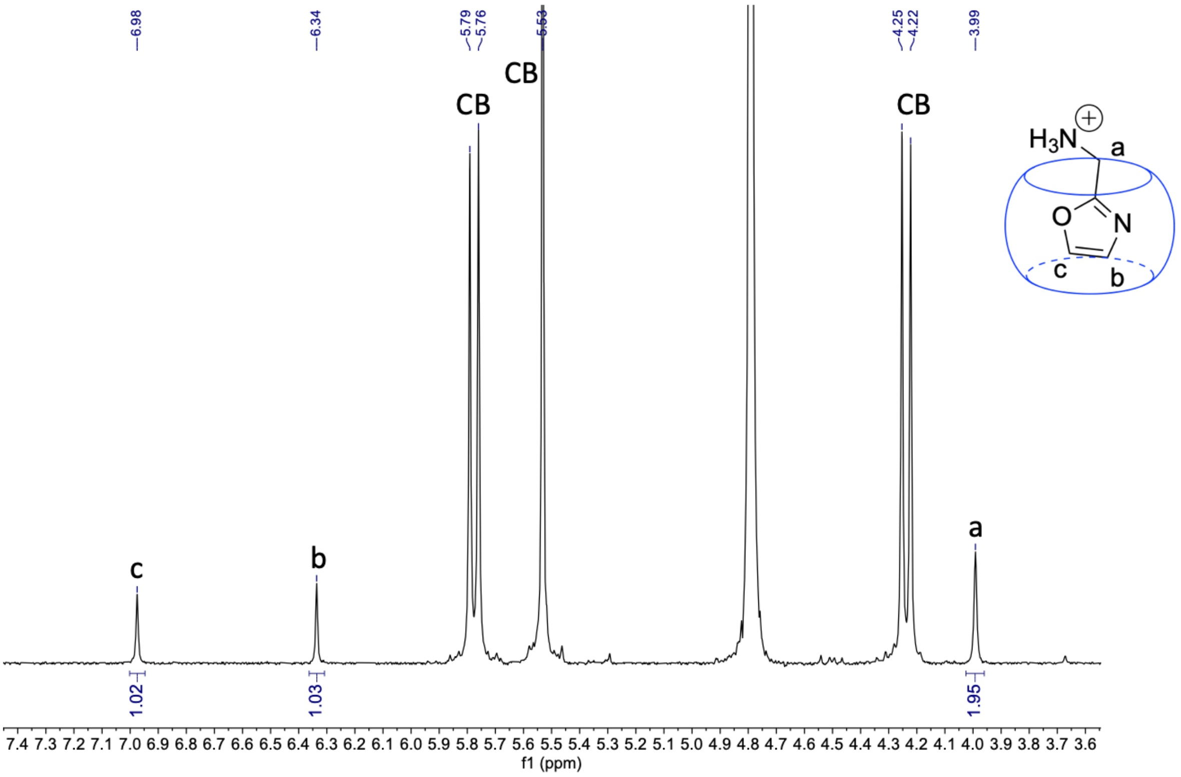

Figure 37. ^{1}H NMR spectrum of complex **19**·CB[7] in D_2O. ^{1}H NMR: δ 7.03 (s, 1H, H^c), 6.39 (s, 1H, H^b), 3.93 (s, 2H, H^a).

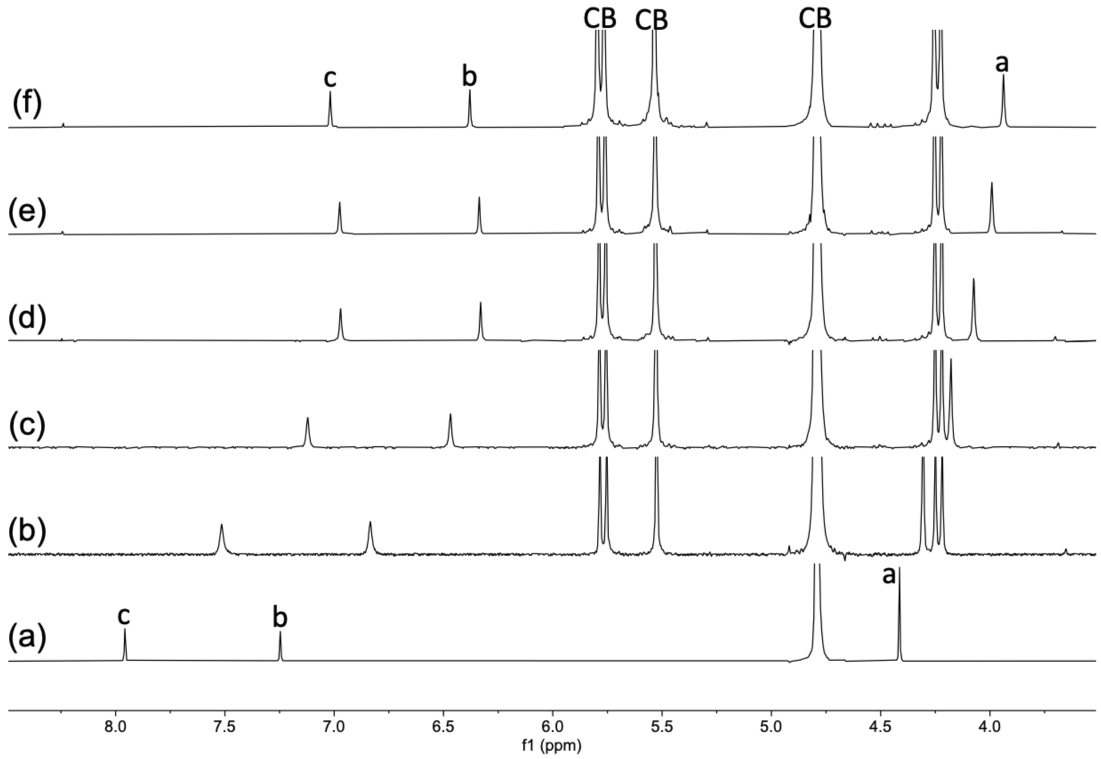

Figure 38. ^{1}H NMR spectra of guest **19** titrated with (a) 0.00, (b) 0.25, (c) 0.50, (d) 0.75, (e) 1.00, and (f) 1.50 equiv CB[7] in D_2O.

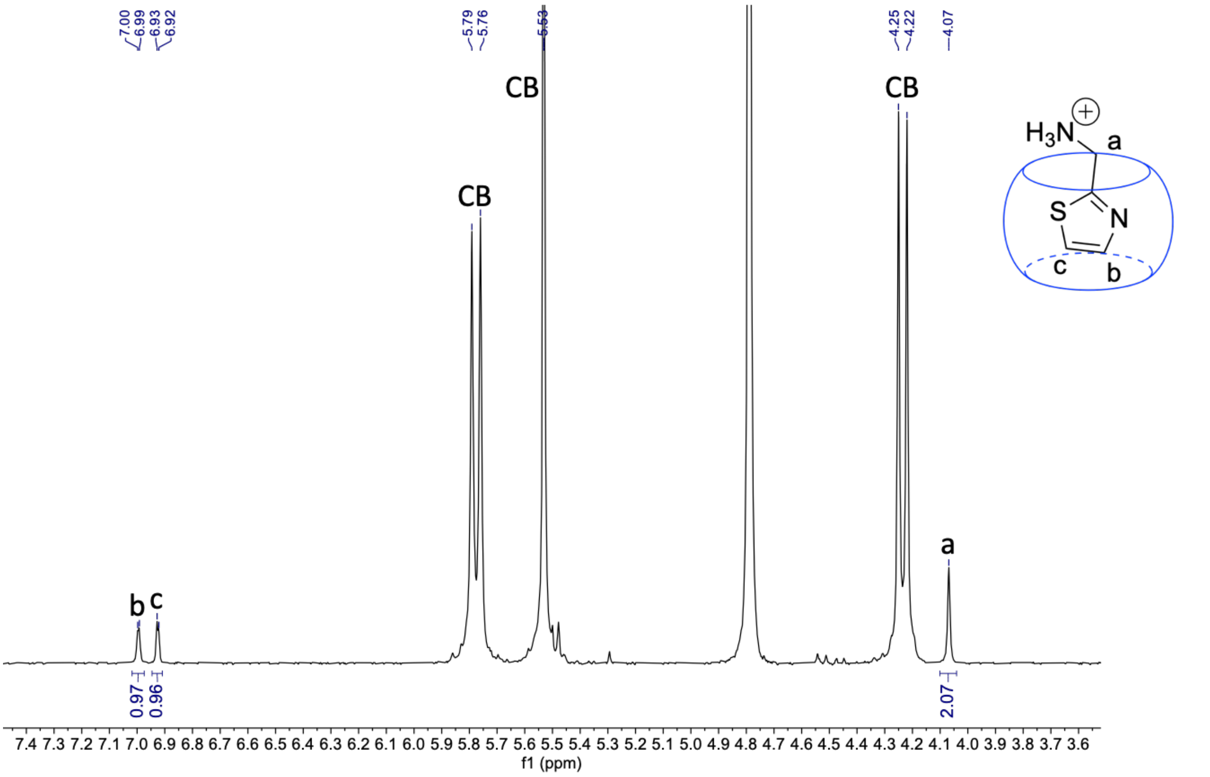

Figure 39. ^{1}H NMR spectrum of complex **20**·CB[7] in D_2O. ^{1}H NMR: δ 7.02 – 6.97 (m, 1H, H^b), 6.92 (m, 1H, H^c), 4.07 (s, 2H, H^a).

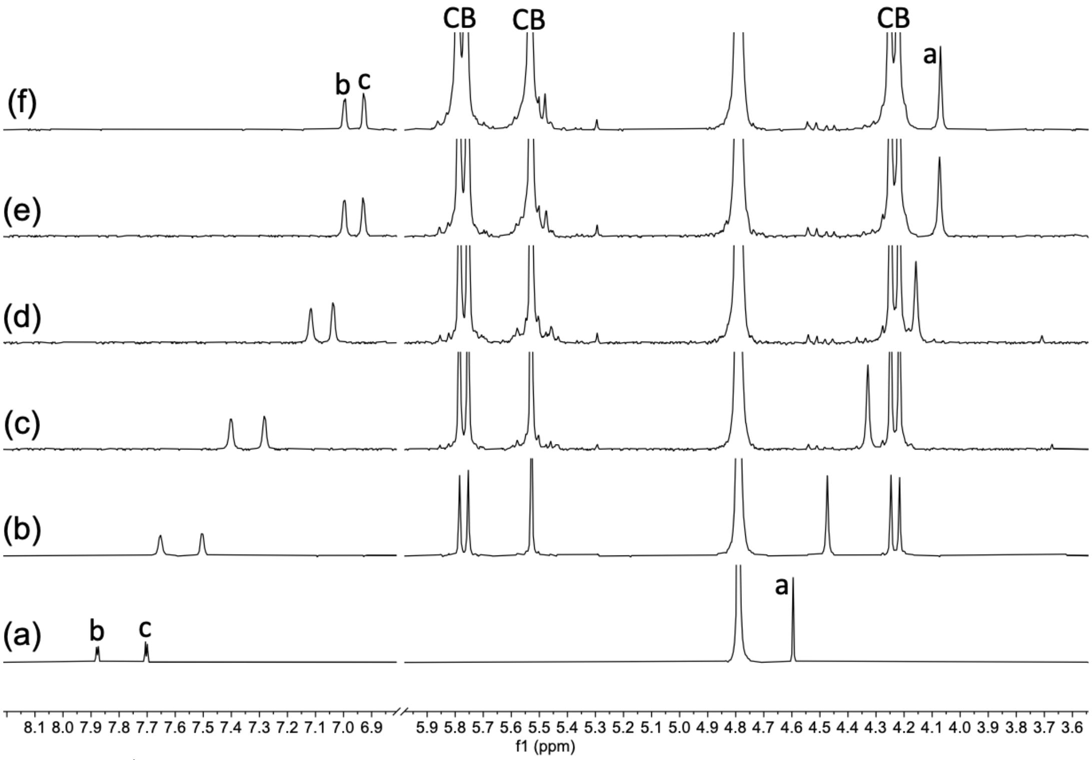

Figure 40. ^{1}H NMR spectra of guest **20** titrated with (a) 0.00, (b) 0.25, (c) 0.50, (d) 0.75, (e) 1.00, and (f) 1.50 equiv CB[7] in D_2O.

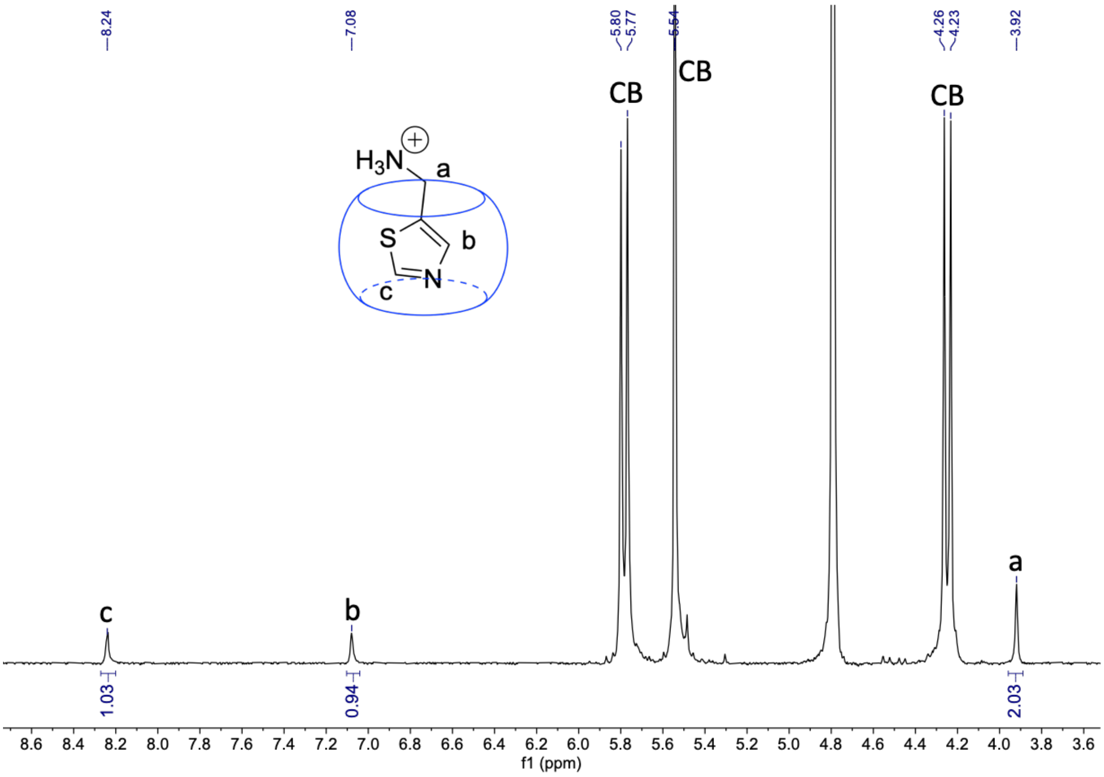

Figure 41. ^{1}H NMR spectrum of complex **21**·CB[7] in D_2O. ^{1}H NMR: δ 8.24 (s, 1H, H^c), 7.08 (s, 1H, H^b), 3.92 (s, 2H, H^a).

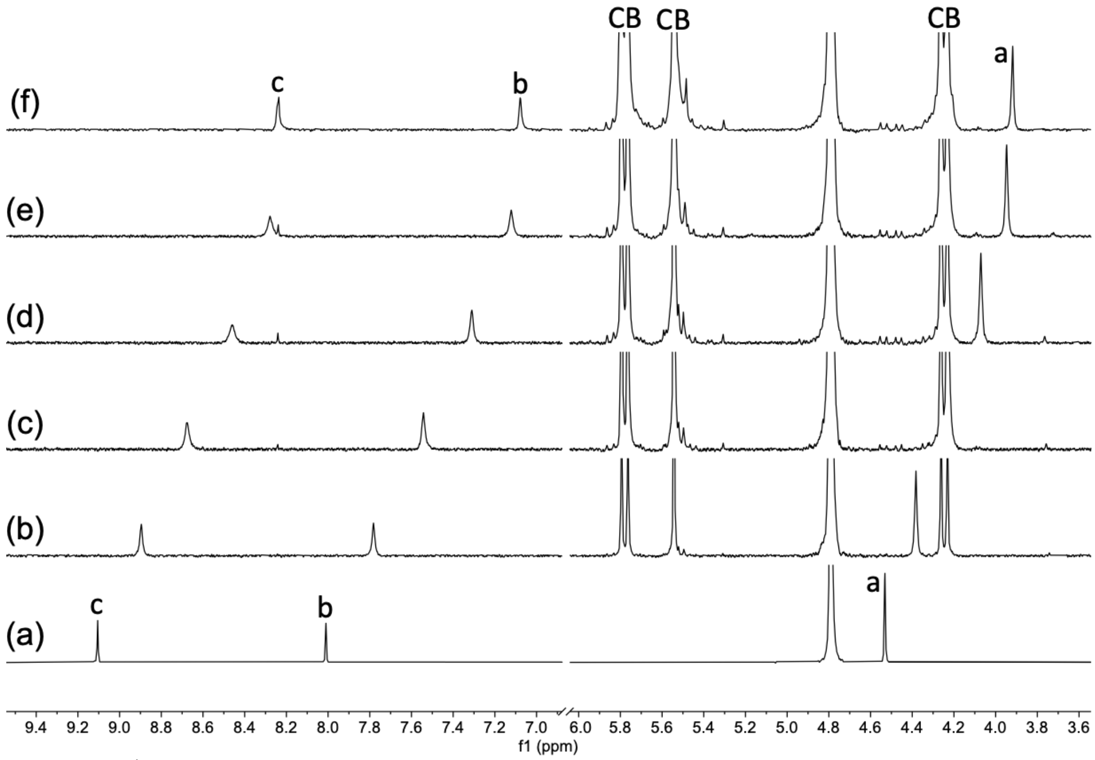

Figure 42. ^{1}H NMR spectra of guest **21** titrated with (a) 0.00, (b) 0.25, (c) 0.50, (d) 0.75, (e) 1.00, and (f) 1.50 equiv CB[7] in D_2O.

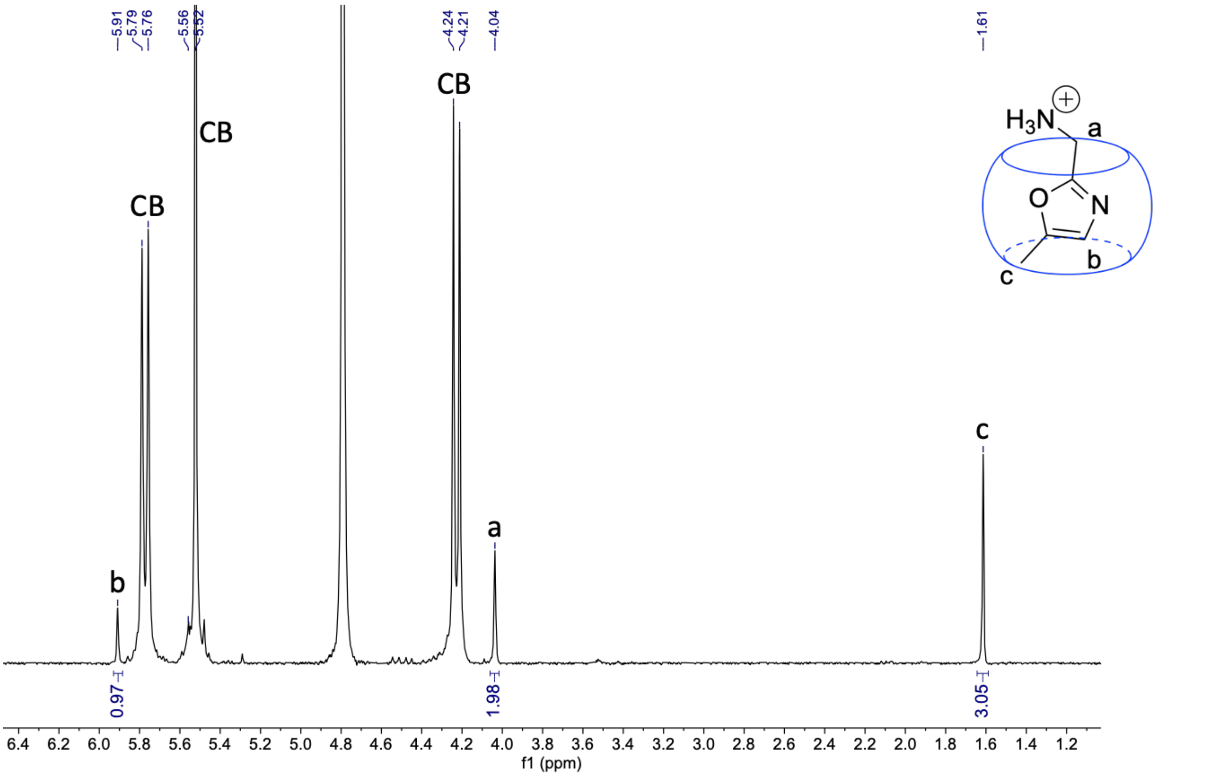

Figure 43. ^{1}H NMR spectrum of complex **22**·CB[7] in D_2O. ^{1}H NMR: δ 5.91 (s, 1H, H^b), 4.04 (s, 2H, H^a), 1.61 (s, 3H, H^c).

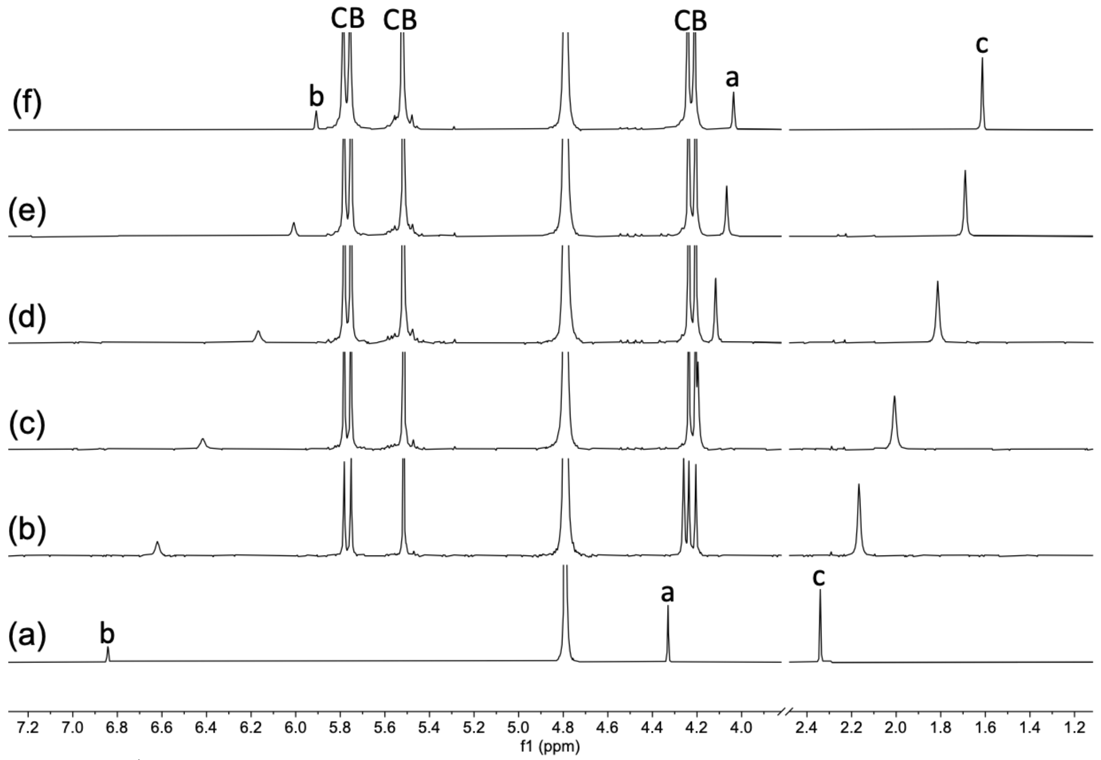

Figure 44. ^{1}H NMR spectra of guest **22** titrated with (a) 0.00, (b) 0.25, (c) 0.50, (d) 0.75, (e) 1.00, and (f) 1.50 equiv CB[7] in D_2O.

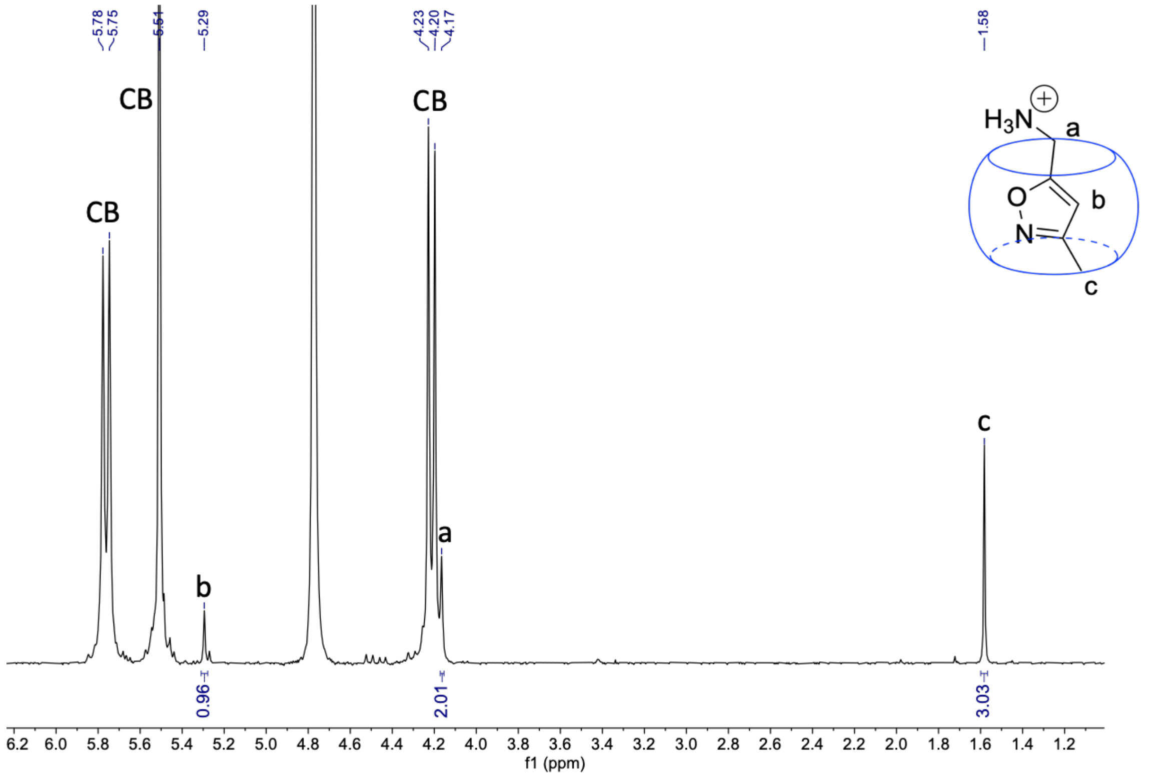

Figure 45. ^{1}H NMR spectrum of complex **23**·CB[7] in D_2O. ^{1}H NMR: δ 5.29 (s, 1H, H^b), 4.17 (s, 2H, H^a), 1.58 (s, 3H, H^c).

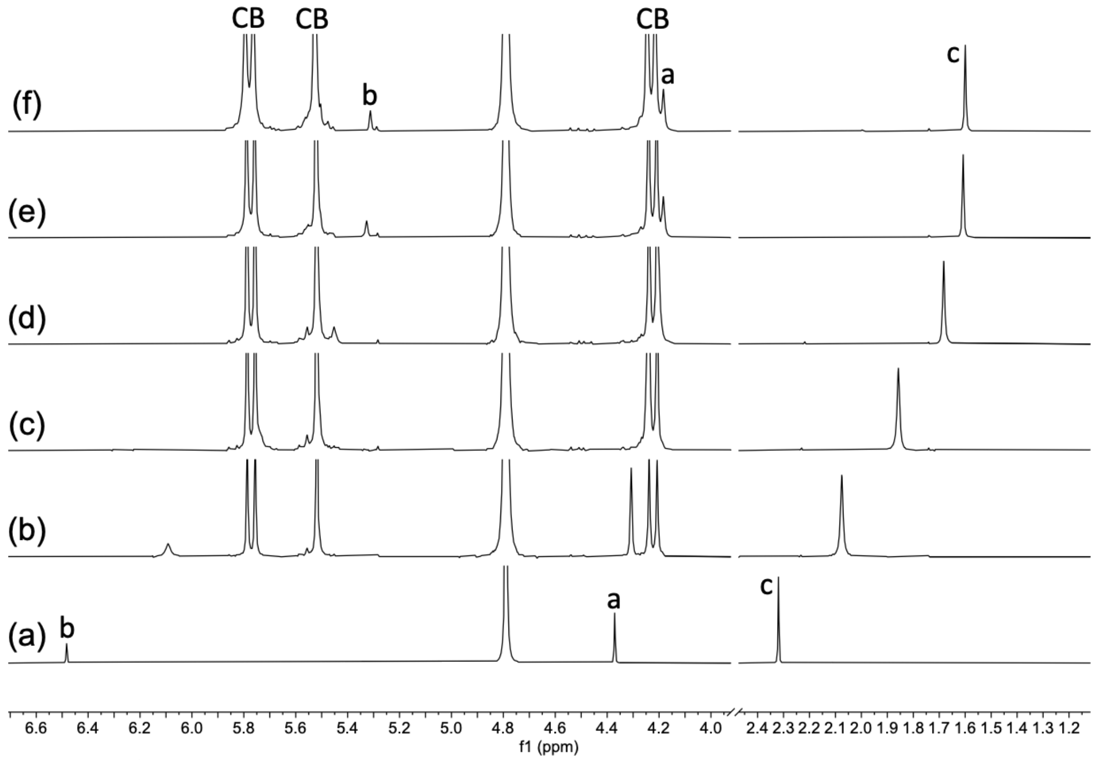

Figure 46. ^{1}H NMR spectra of guest **23** titrated with (a) 0.00, (b) 0.25, (c) 0.50, (d) 0.75, (e) 1.00, and (f) 1.50 equiv CB[7] in D_2O.

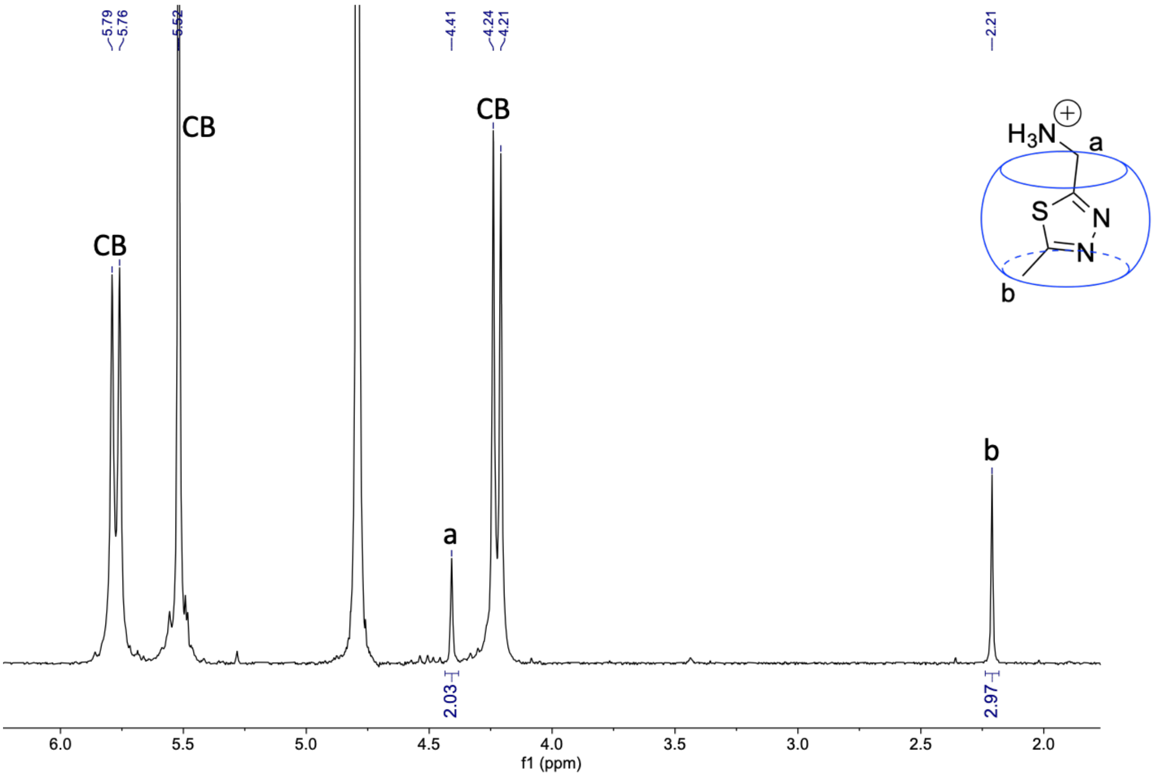

Figure 47. ^{1}H NMR spectrum of complex **24**·CB[7] in D_2O. ^{1}H NMR: δ 4.41 (s, 2H, H^a), 2.21 (s, 3H, H^b).

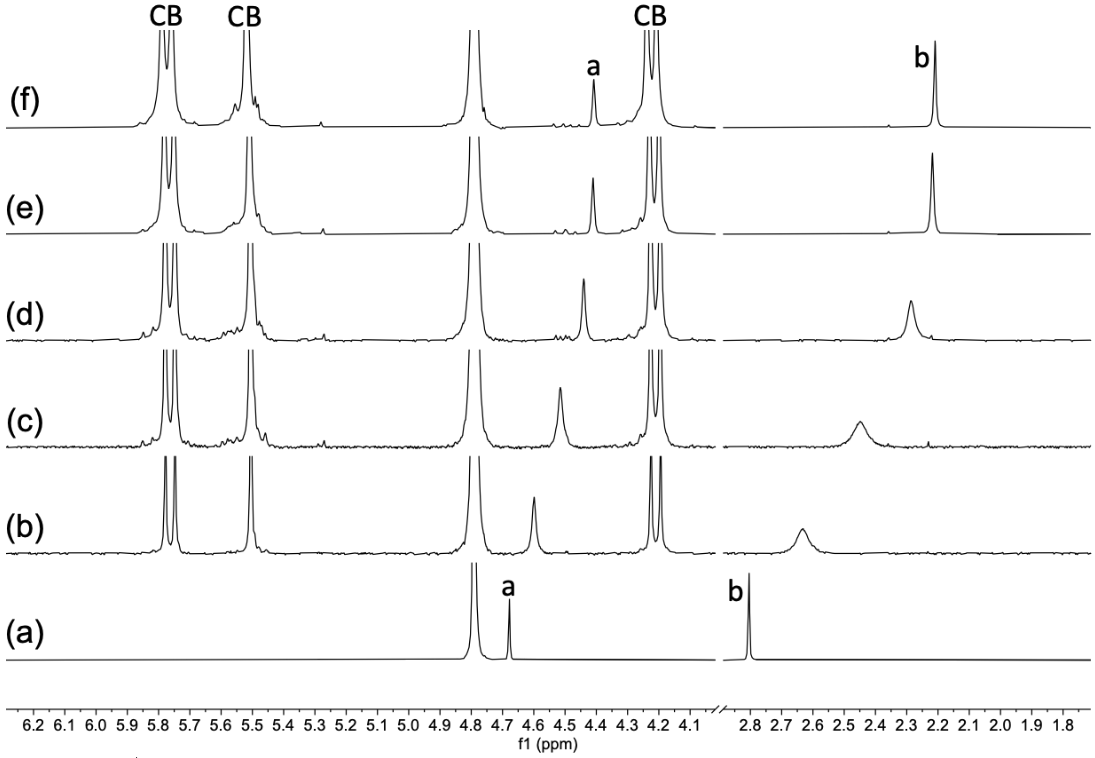

Figure 48. ^{1}H NMR spectra of guest **24** titrated with (a) 0.00, (b) 0.25, (c) 0.50, (d) 0.75, (e) 1.00, and (f) 1.50 equiv CB[7] in D_2O.

3. Isothermal Titration Calorimetry

Isothermal titration calorimetry (ITC) measurements were carried out in triplicate at 25 °C in Milli–Q water. CB[7] (0.1 – 0.4 mM) was placed in the sample cell and titrated with a 10–fold concentrated solution of the guest (1 – 4 mM). Each titration consisted of 30 injections spaced 150 s apart. Enthalpograms were corrected for baseline and dilution heats, then integrated and fitted using AFFINImeter[2] to obtain the binding constant $K_{aq \rightarrow CB}$, and the binding enthalpies and entropies (see Table 1, S35).

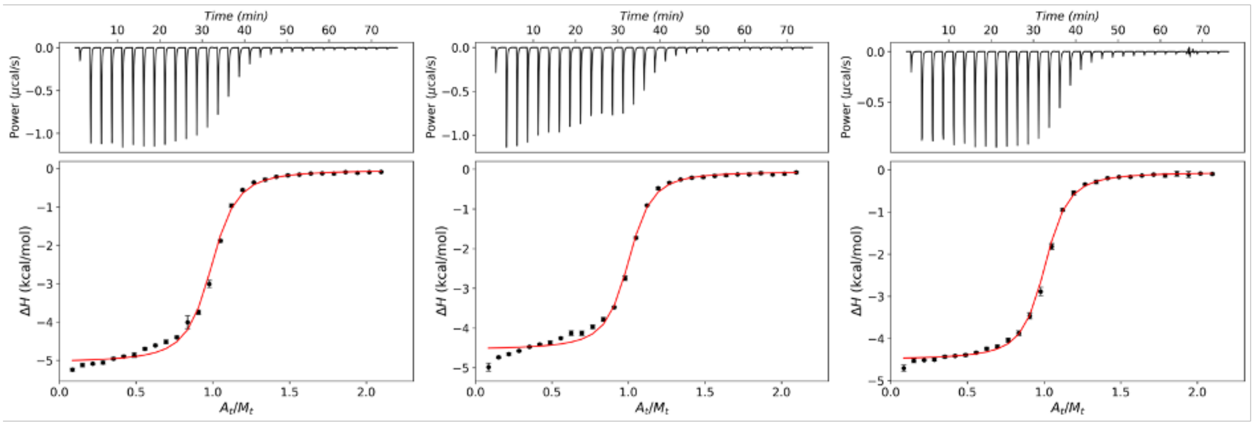

Figure 49. Enthalpograms (top) of ITC titrations of CB[7] (0.20 mM) with **1** (2.0 mM) in water at 25°C. Binding isotherms (bottom) were fit to a 1:1 binding model.

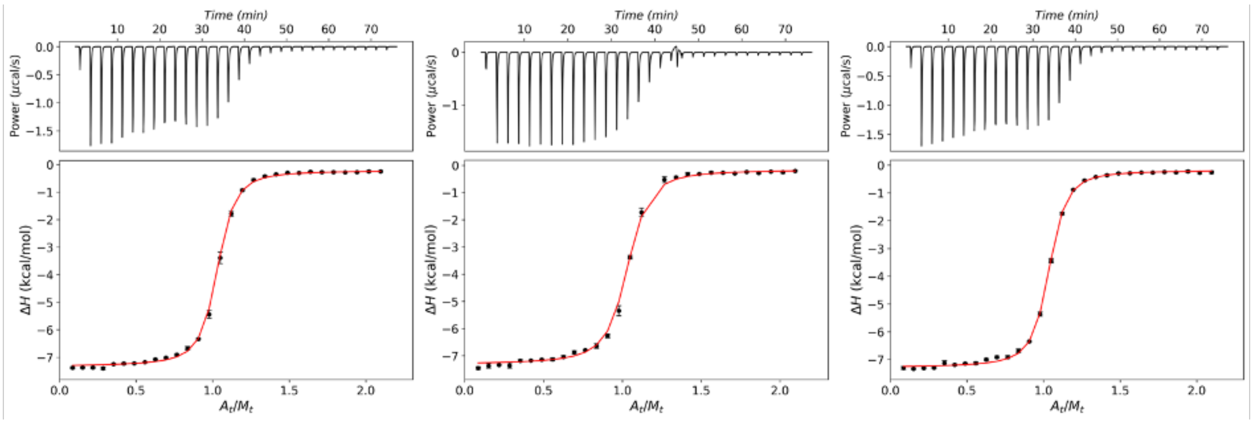

Figure 50. Enthalpograms (top) of ITC titrations of CB[7] (0.20 mM) with **2** (2.0 mM) in water at 25°C. Binding isotherms (bottom) were fit to a 1:1 binding model.

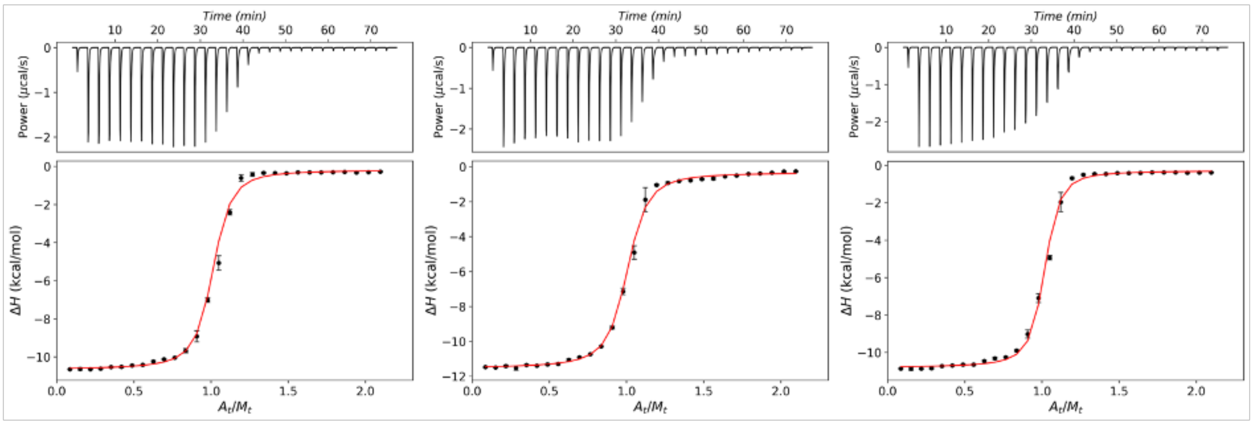

Figure 51. Enthalpograms (top) of ITC titrations of CB[7] (0.20 mM) with **3** (2.0 mM) in water at 25°C. Binding isotherms (bottom) were fit to a 1:1 binding model.

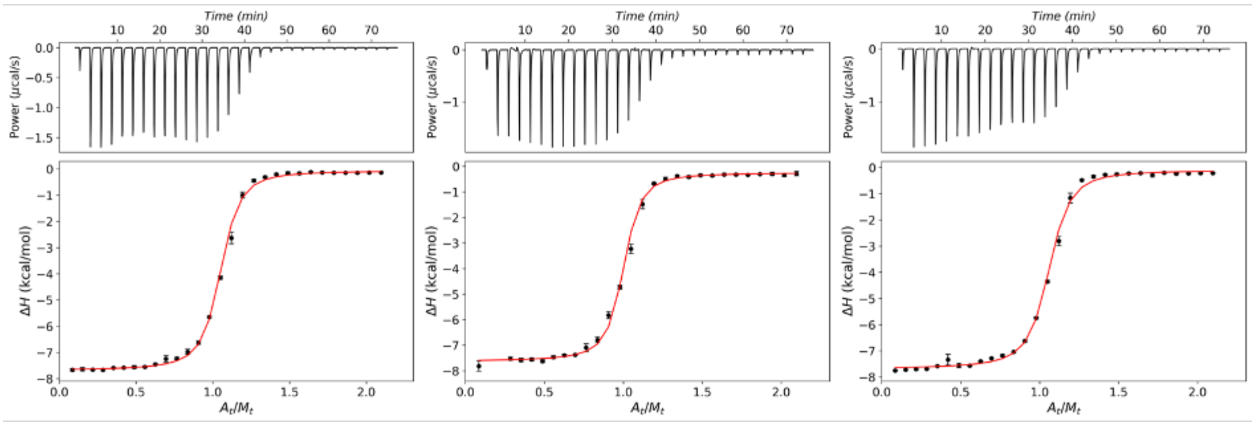

Figure 52. Enthalpograms (top) of ITC titrations of CB[7] (0.20 mM) with **4** (2.0 mM) in water at 25°C. Binding isotherms (bottom) were fit to a 1:1 binding model.

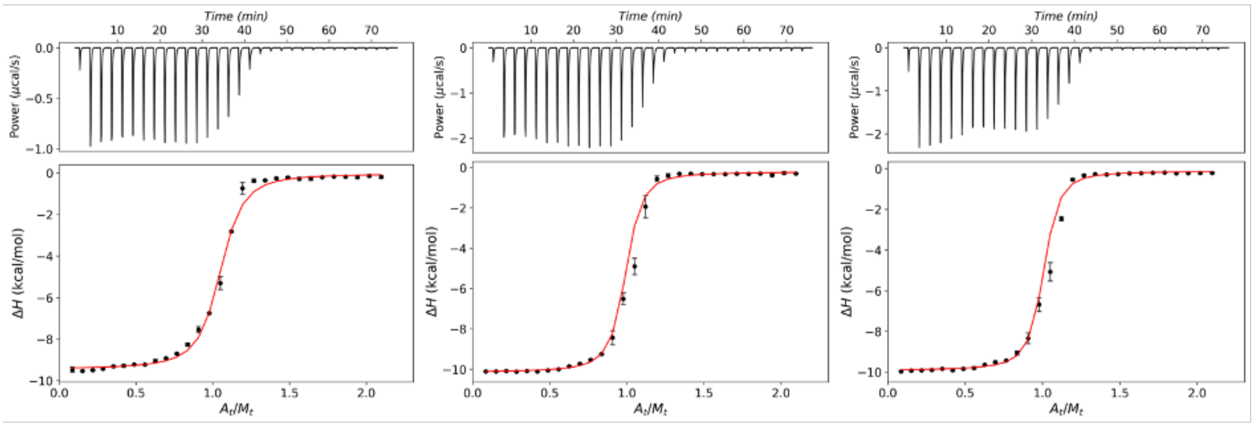

Figure 53. Enthalpograms (top) of ITC titrations of CB[7] (0.20 mM) with **5** (2.0 mM) in water at 25°C. Binding isotherms (bottom) were fit to a 1:1 binding model.

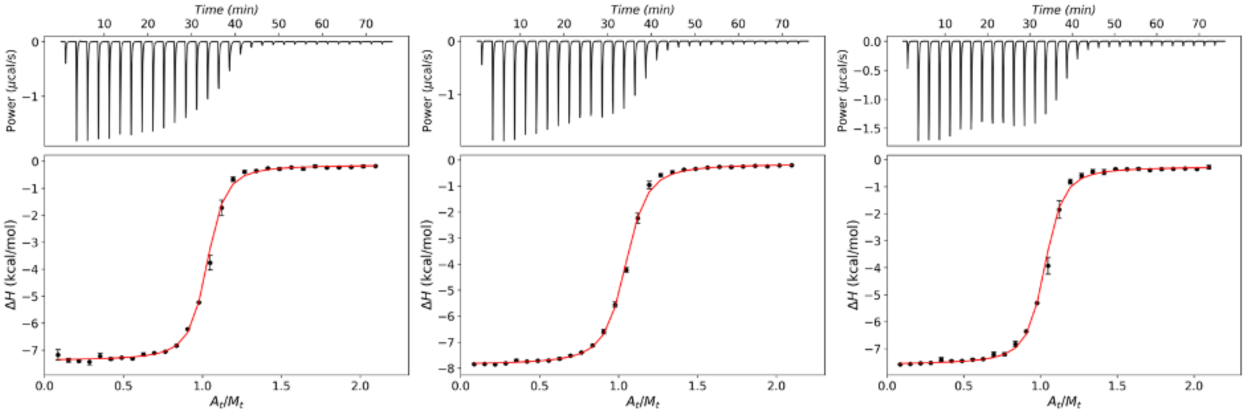

Figure 54. Enthalpograms (top) of ITC titrations of CB[7] (0.20 mM) with **6** (2.0 mM) in water at 25°C. Binding isotherms (bottom) were fit to a 1:1 binding model.

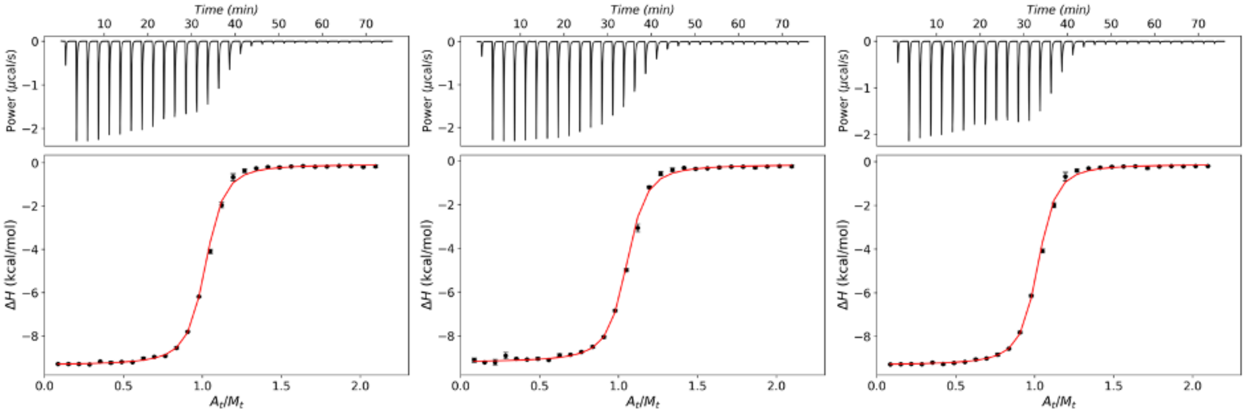

Figure 55. Enthalpograms (top) of ITC titrations of CB[7] (0.20 mM) with **7** (2.0 mM) in water at 25°C. Binding isotherms (bottom) were fit to a 1:1 binding model.

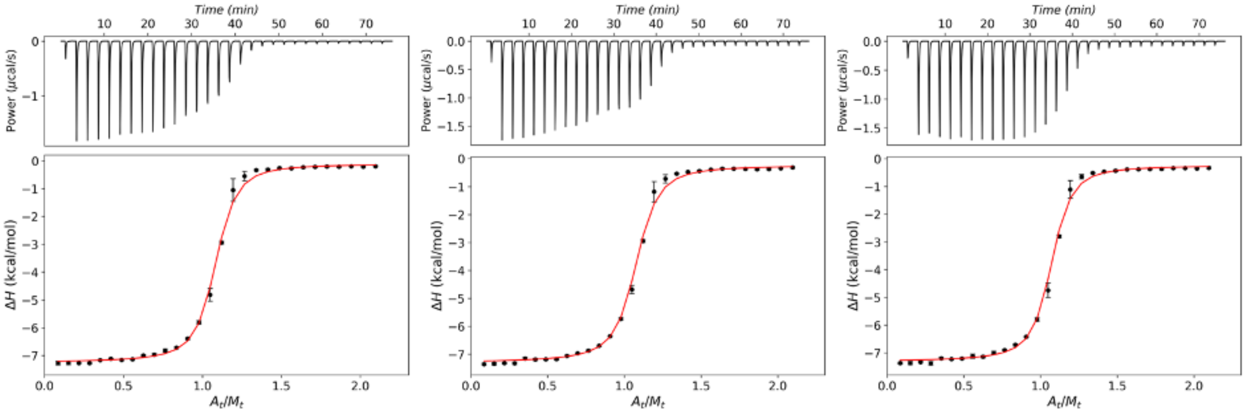

Figure 56. Enthalpograms (top) of ITC titrations of CB[7] (0.20 mM) with **8** (2.0 mM) in water at 25°C. Binding isotherms (bottom) were fit to a 1:1 binding model.

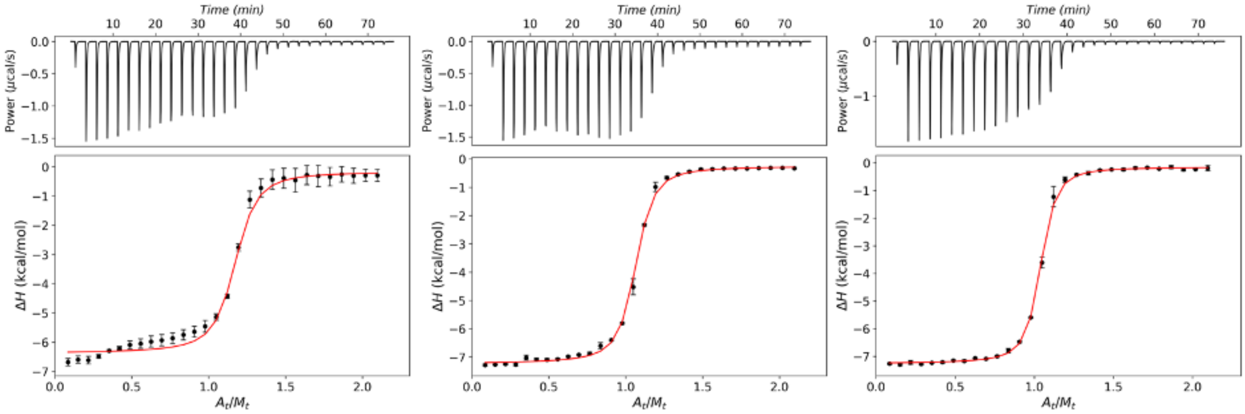

Figure 57. Enthalpograms (top) of ITC titrations of CB[7] (0.20 mM) with **9** (2.0 mM) in water at 25°C. Binding isotherms (bottom) were fit to a 1:1 binding model.

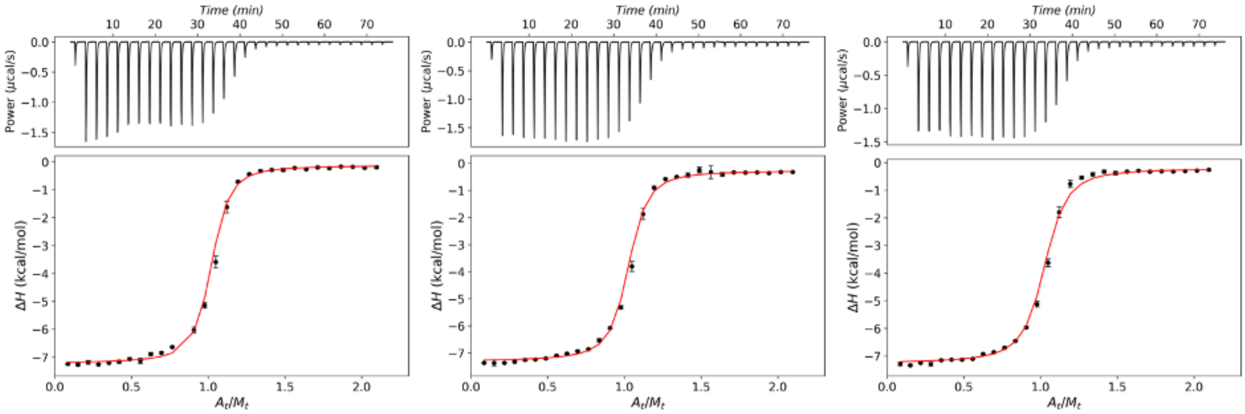

Figure 58. Enthalpograms (top) of ITC titrations of CB[7] (0.20 mM) with **10** (2.0 mM) in water at 25°C. Binding isotherms (bottom) were fit to a 1:1 binding model.

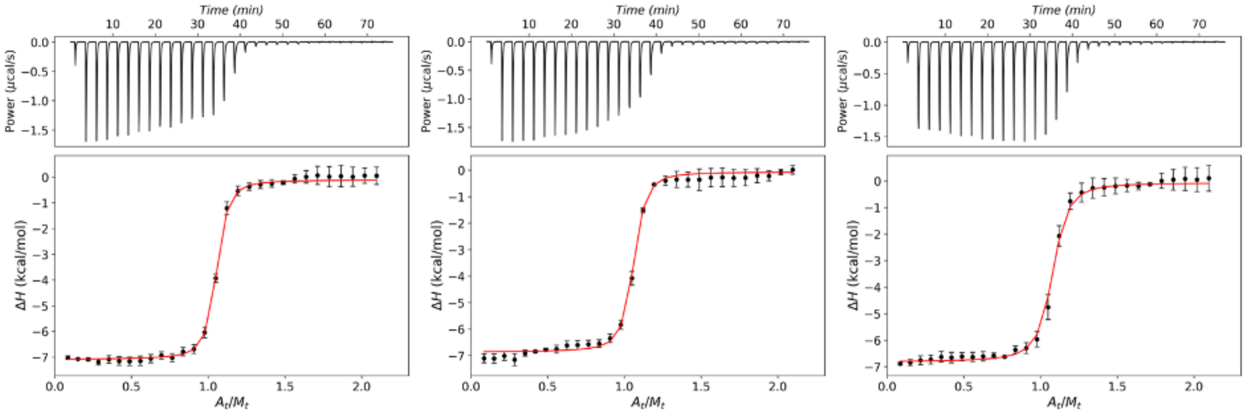

Figure 59. Enthalpograms (top) of ITC titrations of CB[7] (0.20 mM) with **11** (2.0 mM) in water at 25°C. Binding isotherms (bottom) were fit to a 1:1 binding model.

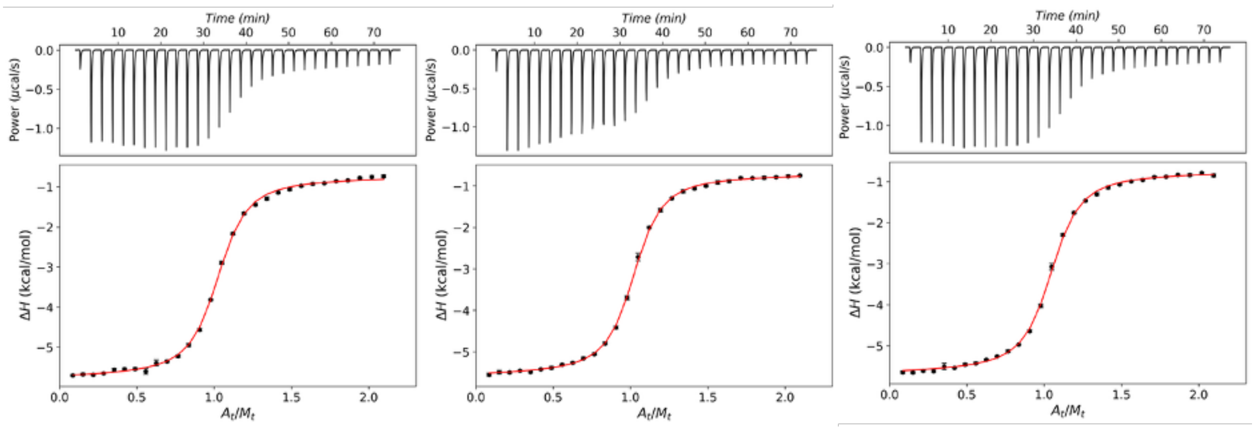

Figure 60. Enthalpograms (top) of ITC titrations of CB[7] (0.20 mM) with **12** (2.0 mM) in water at 25°C. Binding isotherms (bottom) were fit to a 1:1 binding model.

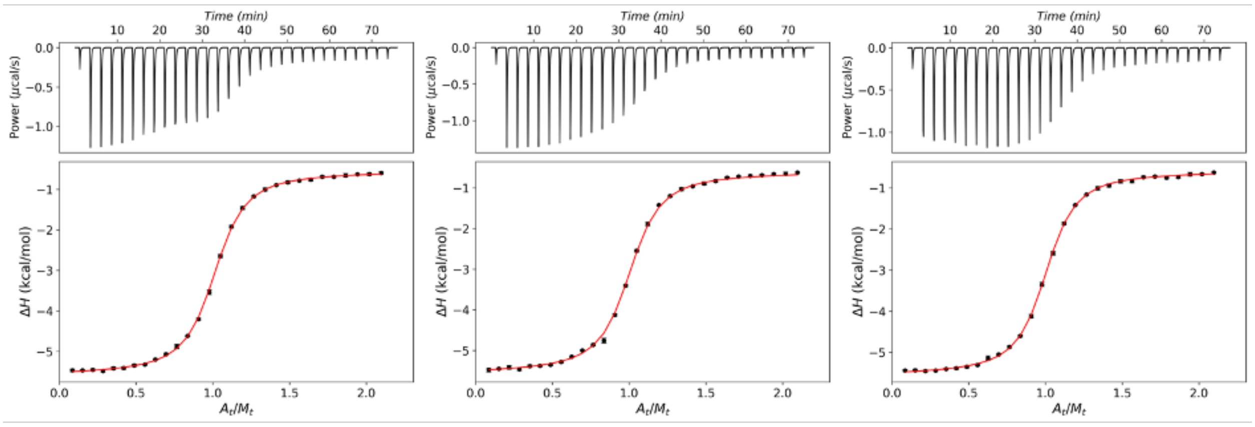

Figure 61. Enthalpograms (top) of ITC titrations of CB[7] (0.20 mM) with **13** (2.0 mM) in water at 25°C. Binding isotherms (bottom) were fit to a 1:1 binding model.

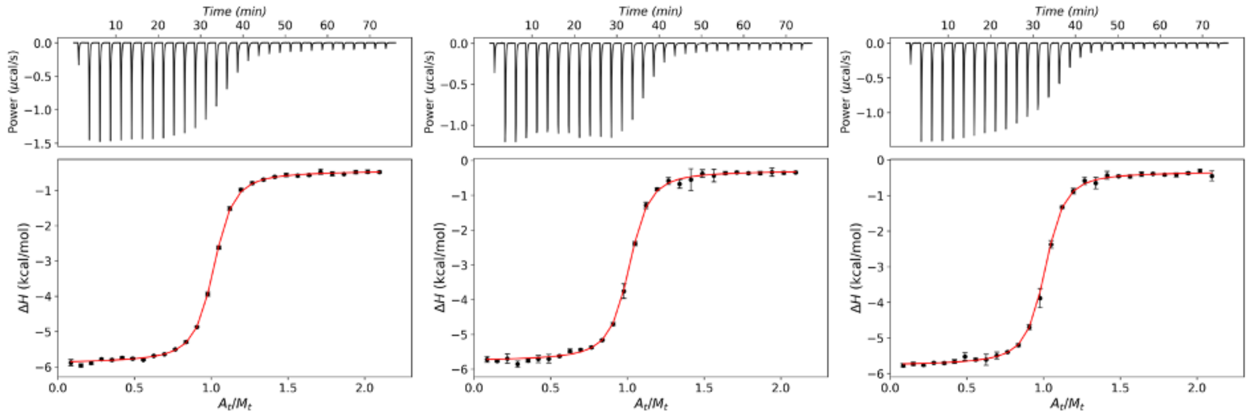

Figure 62. Enthalpograms (top) of ITC titrations of CB[7] (0.20 mM) with **14** (2.0 mM) in water at 25°C. Binding isotherms (bottom) were fit to a 1:1 binding model.

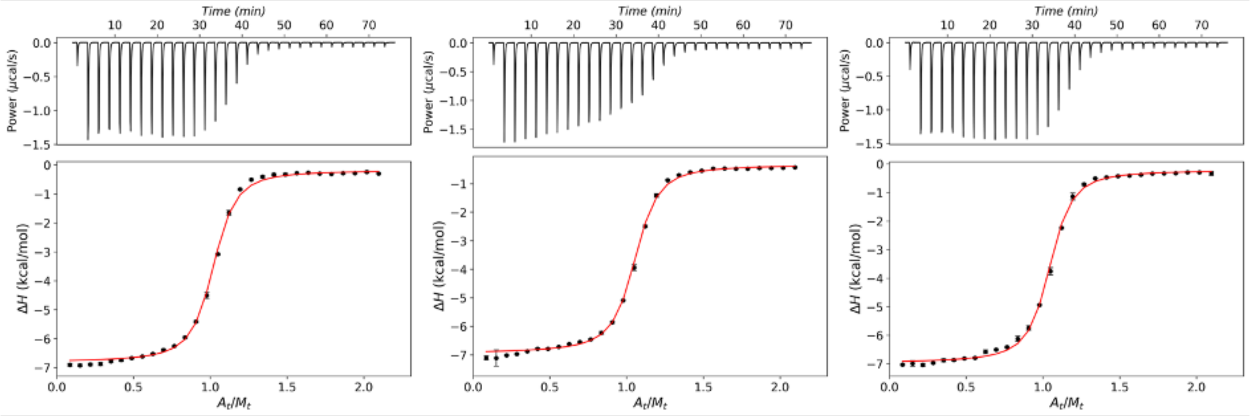

Figure 63. Enthalpograms (top) of ITC titrations of CB[7] (0.20 mM) with **15** (2.0 mM) in water at 25°C. Binding isotherms (bottom) were fit to a 1:1 binding model.

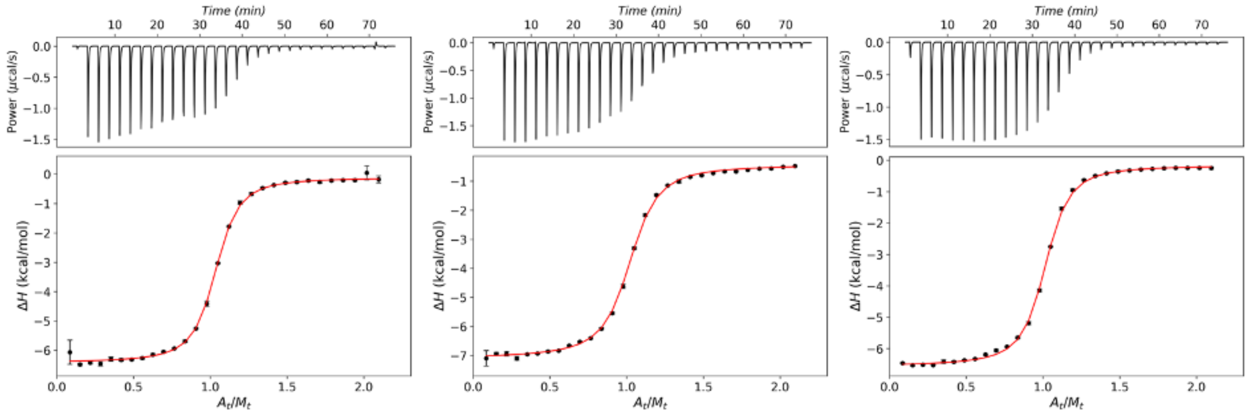

Figure 64. Enthalpograms (top) of ITC titrations of CB[7] (0.20 mM) with **16** (2.0 mM) in water at 25°C. Binding isotherms (bottom) were fit to a 1:1 binding model.

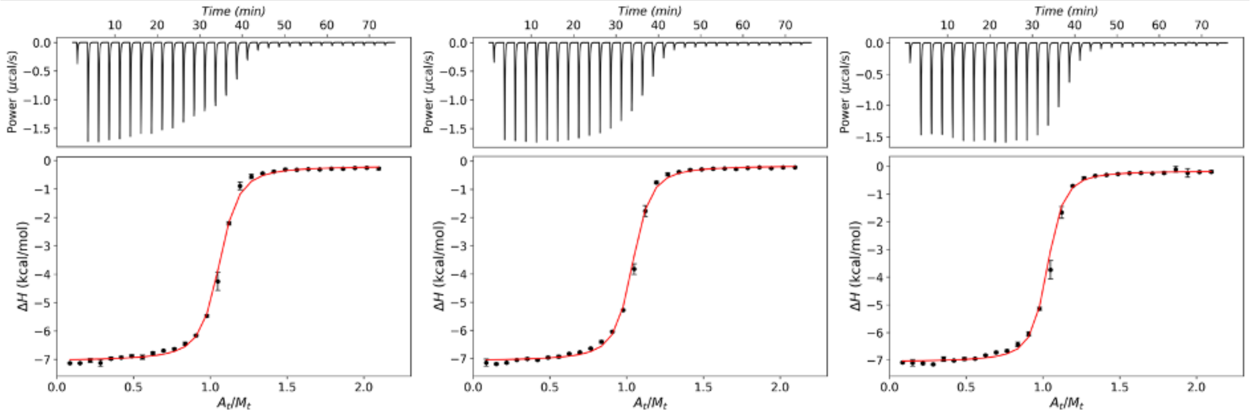

Figure 65. Enthalpograms (top) of ITC titrations of CB[7] (0.20 mM) with **17** (2.0 mM) in water at 25°C. Binding isotherms (bottom) were fit to a 1:1 binding model.

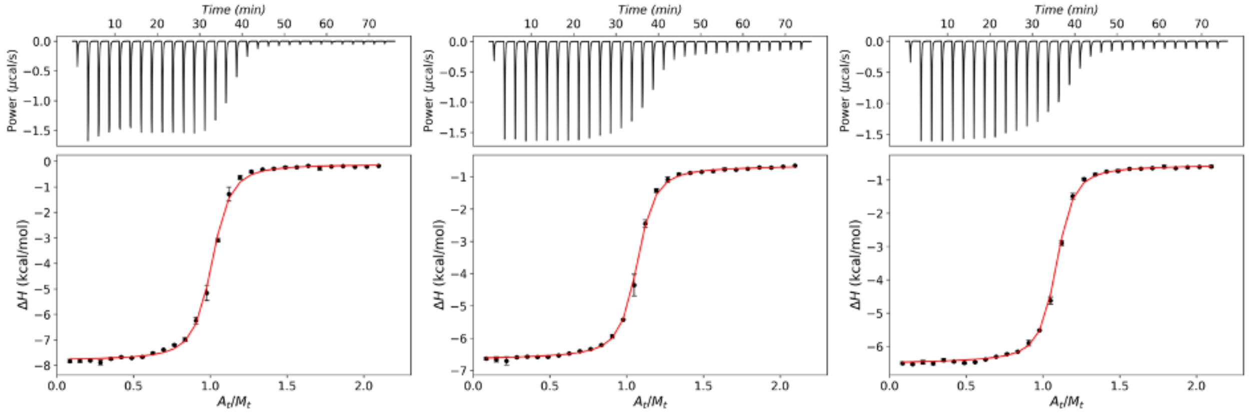

Figure 66. Enthalpograms (top) of ITC titrations of CB[7] (0.20 mM) with **18** (2.0 mM) in water at 25°C. Binding isotherms (bottom) were fit to a 1:1 binding model.

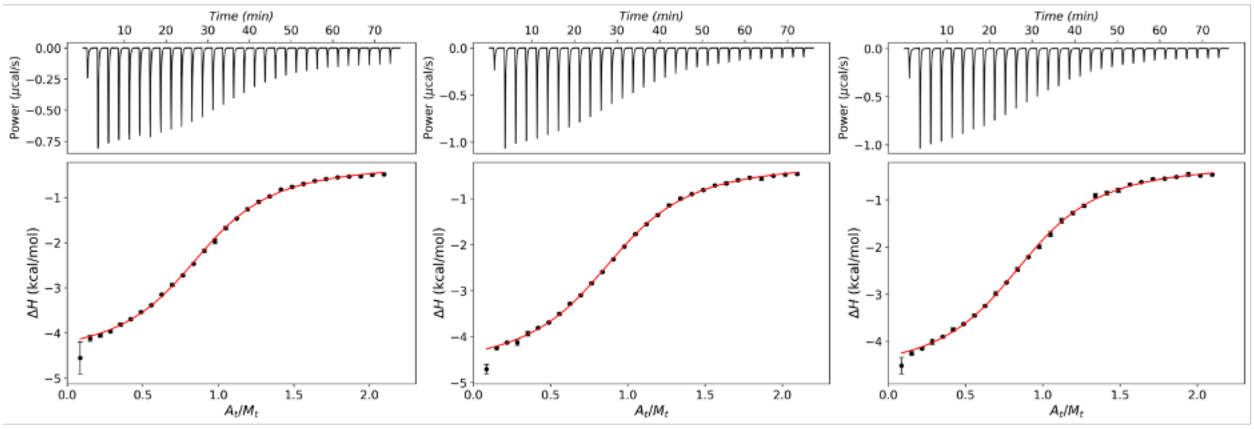

Figure 67. Enthalpograms (top) of ITC titrations of CB[7] (0.20 mM) with **19** (2.0 mM) in water at 25°C. Binding isotherms (bottom) were fit to a 1:1 binding model.

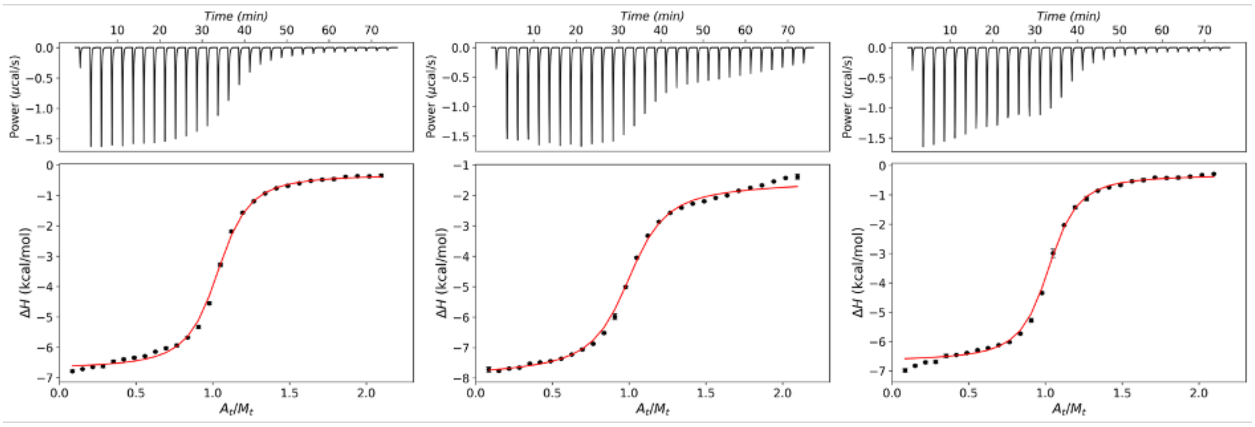

Figure 68. Enthalpograms (top) of ITC titrations of CB[7] (0.20 mM) with **20** (2.0 mM) in water at 25°C. Binding isotherms (bottom) were fit to a 1:1 binding model.

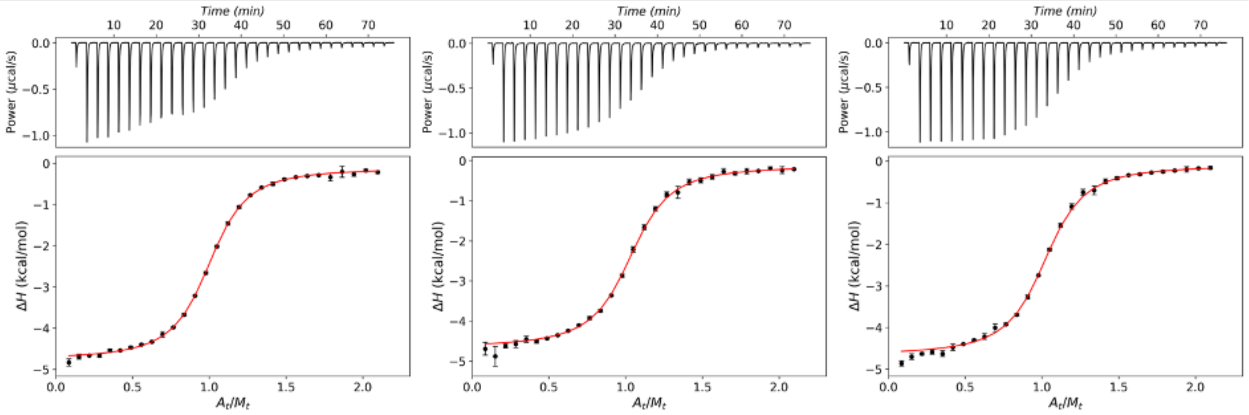

Figure 69. Enthalpograms (top) of ITC titrations of CB[7] (0.20 mM) with **21** (2.0 mM) in water at 25°C. Binding isotherms (bottom) were fit to a 1:1 binding model.

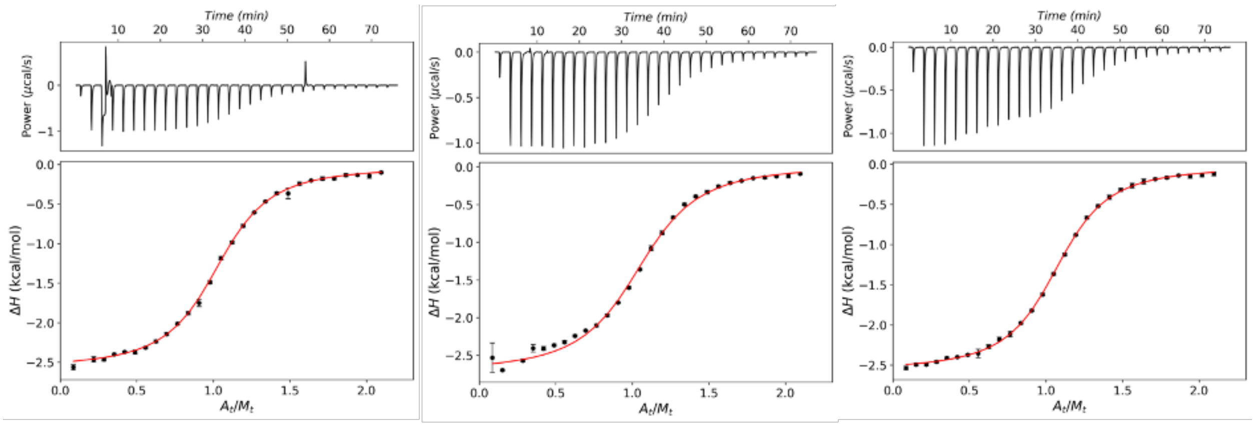

Figure 70. Enthalpograms (top) of ITC titrations of CB[7] (0.4 mM) with **22** (4 mM) in water at 25°C. Binding isotherms (bottom) were fit to a 1:1 binding model.

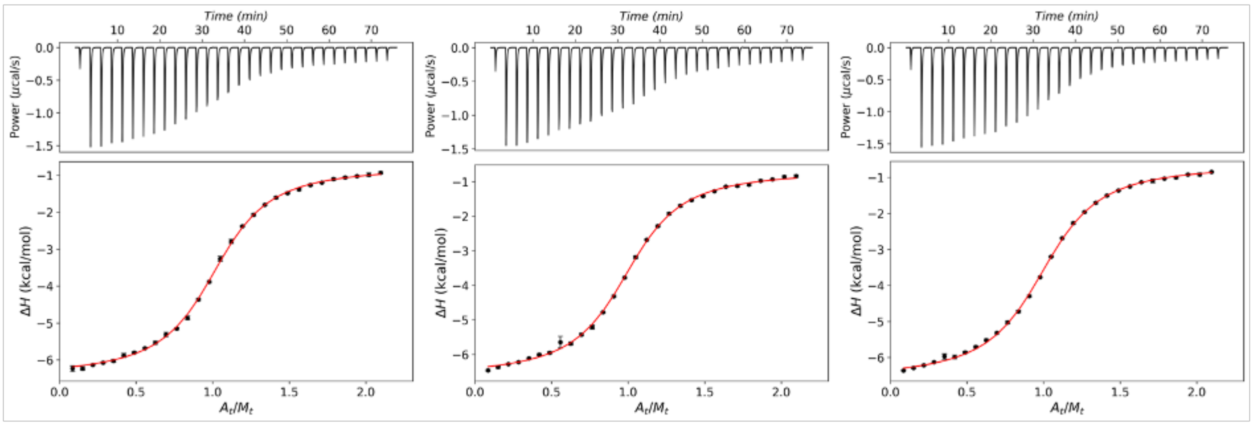

Figure 71. Enthalpograms (top) of ITC titrations of CB[7] (0.20 mM) with **23** (2.0 mM) in water at 25°C. Binding isotherms (bottom) were fit to a 1:1 binding model.

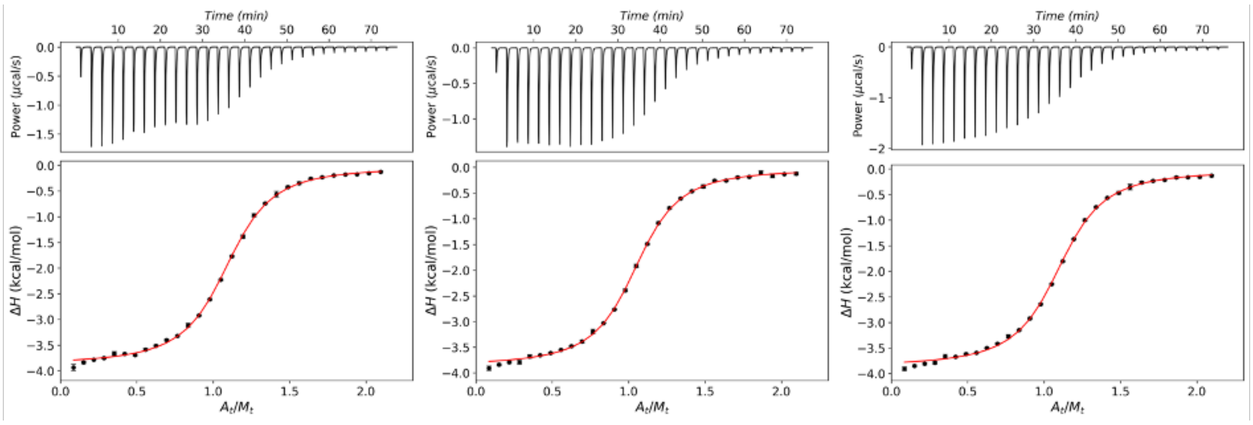

Figure 72. Enthalpograms (top) of ITC titrations of CB[7] (0.4 mM) with **24** (4 mM) in water at 25ºC. Binding isotherms (bottom) were fit to a 1:1 binding model.

Table 1. Thermodynamic parameters obtained from ITC titrations with a 1:1 binding model.[a]

	Run #1				Run #2				Run #3			
	$K_{aq\to CB}$	$\Delta G_{aq\to CB}$	$\Delta H_{aq\to CB}$	$T\Delta S_{aq\to CB}$	$K_{aq\to CB}$	$\Delta G_{aq\to CB}$	$\Delta H_{aq\to CB}$	$T\Delta S_{aq\to CB}$	$K_{aq\to CB}$	$\Delta G_{aq\to CB}$	$\Delta H_{aq\to CB}$	$T\Delta S_{aq\to CB}$
**1**	9.0×10^5 (± 0.2)	−8.12 (± 0.02)	−4.41 (± 0.01)	3.71 (± 0.01)	8.8×10^5 (± 0.3)	−8.11 (± 0.04)	−4.46 (± 0.01)	3.65 (± 0.02)	7.3×10^5 (± 0.3)	−8.00 (± 0.05)	−4.99 (± 0.02)	3.01 (± 0.03)
**2**	1.6×10^6 (± 0.1)	−8.47 (± 0.02)	−7.04 (± 0.01)	1.43 (± 0.01)	1.7×10^6 (± 0.1)	−8.50 (± 0.01)	−7.03 (± 0.01)	1.47 (± 0.01)	1.1×10^6 (± 0.1)	−8.26 (± 0.04)	−7.07 (± 0.01)	1.19 (± 0.03)
**3**	1.5×10^6 (± 0.1)	−8.43 (± 0.03)	−10.38 (± 0.01)	−1.94 (± 0.02)	2.3×10^6 (± 0.2)	−8.67 (± 0.06)	−10.42 (± 0.01)	−1.75 (± 0.04)	1.2×10^6 (± 0.1)	−8.30 (± 0.04)	−11.09 (± 0.02)	−2.79 (± 0.03)
**4**	1.4×10^6 (± 0.1)	−8.38 (± 0.03)	−7.57 (± 0.01)	0.81 (± 0.02)	1.2×10^6 (± 0.1)	−8.30 (± 0.04)	−7.53 (± 0.01)	0.77 (± 0.03)	1.8×10^6 (± 0.1)	−8.52 (± 0.04)	−7.30 (± 0.01)	1.22 (± 0.03)
**5**	2.1×10^6 (± 0.2)	−8.63 (± 0.06)	−9.75 (± 0.01)	−1.12 (± 0.05)	1.9×10^6 (± 0.1)	−8.58 (± 0.03)	−9.86 (± 0.01)	−1.28 (± 0.02)	2.0×10^6 (± 0.1)	−8.59 (± 0.05)	−9.37 (± 0.02)	−0.78 (± 0.03)
**6**	1.6×10^6 (± 0.1)	−8.48 (± 0.03)	−7.20 (± 0.01)	1.28 (± 0.02)	1.5×10^6 (± 0.1)	−8.43 (± 0.03)	−7.23 (± 0.01)	1.20 (± 0.02)	1.2×10^6 (± 0.1)	−8.29 (± 0.02)	−7.65 (± 0.01)	0.64 (± 0.01)
**7**	1.5×10^6 (± 0.1)	−8.43 (± 0.02)	−9.24 (± 0.01)	−0.81 (± 0.02)	1.6×10^6 (± 0.1)	−8.47 (± 0.02)	−9.14 (± 0.01)	−0.67 (± 0.02)	1.4×10^6 (± 0.1)	−8.40 (± 0.03)	−8.97 (± 0.01)	−0.57 (± 0.02)
**8**	1.3×10^6 (± 0.1)	−8.34 (± 0.05)	−7.08 (± 0.01)	1.26 (± 0.03)	1.3×10^6 (± 0.1)	−8.36 (± 0.04)	−6.99 (± 0.01)	1.37 (± 0.03)	1.1×10^6 (± 0.1)	−8.23 (± 0.03)	−6.97 (± 0.01)	1.26 (± 0.02)
**9**	1.2×10^6 (± 0.1)	−8.32 (± 0.06)	−6.14 (± 0.02)	2.18 (± 0.04)	2.4×10^6 (± 0.1)	−8.70 (± 0.02)	−7.06 (± 0.01)	1.64 (± 0.01)	1.6×10^6 (± 0.1)	−8.48 (± 0.03)	−6.89 (± 0.01)	1.59 (± 0.02)
**10**	1.6×10^6 (± 0.1)	−8.45 (± 0.05)	−7.04 (± 0.01)	1.41 (± 0.03)	1.0×10^6 (± 0.1)	−8.20 (± 0.02)	−6.97 (± 0.01)	1.22 (± 0.02)	1.4×10^6 (± 0.1)	−8.38 (± 0.03)	−6.95 (± 0.01)	1.43 (± 0.02)
**11**	4.3×10^6 (± 0.1)	−9.06 (± 0.03)	−6.95 (± 0.01)	2.11 (± 0.02)	2.3×10^6 (± 0.2)	−8.67 (± 0.07)	−6.71 (± 0.02)	1.96 (± 0.05)	3.8×10^6 (± 0.1)	−8.98 (± 0.03)	−6.77 (± 0.01)	2.21 (± 0.02)
**12**	6.3×10^5 (± 0.1)	−7.91 (± 0.01)	−4.66 (± 0.01)	3.25 (± 0.01)	6.0×10^5 (± 0.1)	−7.89 (± 0.02)	−4.70 (± 0.01)	3.19 (± 0.01)	5.9×10^5 (± 0.1)	−7.87 (± 0.01)	−4.80 (± 0.01)	3.07 (± 0.01)
**13**	5.0×10^5 (± 0.1)	−7.78 (± 0.01)	−4.85 (± 0.01)	2.93 (± 0.01)	5.3×10^5 (± 0.1)	−7.81 (± 0.01)	−4.78 (± 0.01)	3.03 (± 0.01)	4.9×10^5 (± 0.1)	−7.77 (± 0.03)	−4.76 (± 0.01)	3.00 (± 0.02)
**14**	1.3×10^6 (± 0.1)	−8.33 (± 0.01)	−5.32 (± 0.01)	3.01 (± 0.01)	1.3×10^6 (± 0.1)	−8.34 (± 0.01)	−5.33 (± 0.01)	3.01 (± 0.01)	1.2×10^6 (± 0.1)	−8.31 (± 0.02)	−5.37 (± 0.01)	2.94 (± 0.01)
**15**	9.7×10^5 (± 0.3)	−8.17 (± 0.03)	−6.55 (± 0.01)	1.62 (± 0.02)	1.0×10^6 (± 0.1)	−8.19 (± 0.04)	−6.67 (± 0.01)	1.53 (± 0.03)	9.5×10^5 (± 0.3)	−8.16 (± 0.04)	−6.48 (± 0.02)	1.68 (± 0.02)
**16**	9.2×10^5 (± 0.1)	−8.14 (± 0.01)	−6.25 (± 0.01)	1.89 (± 0.01)	8.4×10^5 (± 0.2)	−8.08 (± 0.03)	−6.33 (± 0.01)	1.76 (± 0.02)	6.2×10^5 (± 0.2)	−7.90 (± 0.04)	−6.52 (± 0.02)	1.38 (± 0.02)
**17**	1.4×10^6 (± 0.1)	−8.38 (± 0.04)	−6.78 (± 0.01)	1.60 (± 0.02)	1.9×10^6 (± 0.1)	−8.58 (± 0.04)	−6.84 (± 0.01)	1.74 (± 0.03)	1.6×10^6 (± 0.1)	−8.45 (± 0.03)	−6.86 (± 0.01)	1.59 (± 0.02)
**18**	1.4×10^6 (± 0.1)	−8.39 (± 0.03)	−7.63 (± 0.01)	0.76 (± 0.03)	1.8×10^6 (± 0.1)	−8.54 (± 0.02)	−5.80 (± 0.01)	2.74 (± 0.01)	1.7×10^6 (± 0.1)	−8.49 (± 0.02)	−5.81 (± 0.01)	2.68 (± 0.02)
**19**	6.6×10^4 (± 0.1)	−6.58 (± 0.02)	−4.15 (± 0.01)	2.42 (± 0.01)	6.3×10^4 (± 0.1)	−6.55 (± 0.02)	−4.36 (± 0.01)	2.18 (± 0.01)	6.3×10^4 (± 0.1)	−6.55 (± 0.04)	−4.33 (± 0.02)	2.22 (± 0.02)
**20**	3.6×10^5 (± 0.1)	−7.57 (± 0.06)	−5.84 (± 0.03)	1.73 (± 0.04)	5.0×10^5 (± 0.1)	−7.77 (± 0.04)	−6.29 (± 0.02)	1.49 (± 0.02)	5.7×10^5 (± 0.2)	−7.85 (± 0.06)	−6.24 (± 0.02)	1.61 (± 0.03)
**21**	3.3×10^5 (± 0.1)	−7.52 (± 0.01)	−4.59 (± 0.01)	2.93 (± 0.01)	3.1×10^5 (± 0.1)	−7.49 (± 0.03)	−4.52 (± 0.01)	2.98 (± 0.02)	3.1×10^5 (± 0.1)	−7.49 (± 0.02)	−4.48 (± 0.01)	3.01 (± 0.01)
**22**	8.5×10^4 (± 0.1)	−6.73 (± 0.01)	−2.53 (± 0.01)	4.20 (± 0.01)	9.4×10^4 (± 0.1)	−6.78 (± 0.01)	−2.52 (± 0.01)	4.26 (± 0.01)	6.7×10^4 (± 0.4)	−6.59 (± 0.09)	−2.74 (± 0.04)	3.85 (± 0.05)
**23**	1.6×10^5 (± 0.1)	−7.10 (± 0.01)	−5.37 (± 0.01)	1.74 (± 0.01)	1.5×10^5 (± 0.1)	−7.05 (± 0.03)	−5.65 (± 0.01)	1.40 (± 0.02)	1.6×10^5 (± 0.1)	−7.09 (± 0.02)	−5.65 (± 0.01)	1.44 (± 0.01)
**24**	3.4×10^5 (± 0.1)	−7.55 (± 0.05)	−5.87 (± 0.02)	1.68 (± 0.03)	1.2×10^5 (± 0.1)	−6.95 (± 0.01)	−3.82 (± 0.01)	3.12 (± 0.01)	1.3×10^5 (± 0.1)	−6.95 (± 0.02)	−3.81 (± 0.01)	3.14 (± 0.01)

[a] Binding affinities in M^{-1}; all energy terms in kcal/mol.

Error calculation

The standard error of the mean (SEM) on the free energies is obtained from equation 1 (*n* = 3);

$$\sigma_{\text{runs}} = \sqrt{\frac{1}{n(n-1)}\sum_{i=1}^{n}\left(\Delta G_i - \overline{\Delta G}\right)^2} \quad (1)$$

The contribution to SEM from fitting is obtained from equation 2 (*n* = 3);

$$\sigma_{\text{mean,fit}} = \sqrt{\frac{1}{n^2}\sum_{i=1}^{n}\sigma_{\text{i,fit}}^{\,2}} \quad (2)$$

The combined SEM is obtained from equation 3:

$$\sigma_{\Delta\text{G}} = \sqrt{\sigma_{\text{runs}}^{\,2} + \sigma_{\text{mean,fit}}^{\,2}} \quad (3)$$

Equation 4 propagates the error to binding affinity $K_{\text{aq}\to\text{CB}}$.

$$\sigma_K = \frac{K_{\text{aq}\to\text{CB}}}{RT}\sigma_{\Delta\text{G}} \quad (4)$$

4. Computational details

Calculations were carried out with the Turbomole suite of programs (TM; version 7.3),[3] and the GFN2–xTB software[4-7] on the OSC Pitzer Cluster of the Ohio Supercomputer Center in Columbus, OH (23,392–core Dell Intel Xeon E5–2680 v4 and 10,240–core Dell Intel Gold 6148 machines). Free energies of solvation were obtained with COSMO*therm* X23,[8] and energy decomposition analysis was carried out with the SCM AMS2025.104 software[9] on a MacBook Pro (OS 15.6.1; Apple M1 Max chip, 64 GB memory). Guest volumes, delimited by a 0.002 electron/Bohr^3 isodensity surface, were calculated at the semi–empirical PM6 level with Spartan 24.[10]

Free energies of solvation of truncated guests (ammonium salts **1** – **24** minus their $CH_2NH_3^+$ head groups) were calculated using the procedure described by Klamt and coworkers using TM. All guests were optimized (a) in the gas phase, at the BP86/def–TZVP level of theory,[11-13] and (b) in solution at the same level of theory using the COSMO solvation model.[14-16] Energies were then refined in single–point calculations at the BP86/def2–TZVPD level. The output files from these single–point calculations in the gas phase and in solution (.energy and .cosmo extensions, respectively) were then treated with COSMO*therm*X23[8] and the BP_TZVPD_FINE_23 parametrization model to extract free Gibbs energies of solvation.

Complexes **25a**·CB[7], **25b**·CB[7], **26a**·CB[7] and **26b**·CB[7] were optimized with GFN2–xTB in conjunction with the ALPB solvation model[17] for water. Energy decomposition analysis was then carried out with single-point calculations at the ZORA-PBE-D3(BJ)/TZ2P level.[18-20]

Multiple linear regression analysis was carried out with Excel (version 16.105.1 for Mac OS) and the LINEST function.

Table 2. Binding affinities of guests **1** – **24** to CB[7]; parameters and free energy terms associated with the recognition process and the empirical prediction models (complement to Table 1 in the manuscript).

	$K_{aq\rightarrow CB}$ [a]	$\Delta G_{aq\rightarrow CB}$ [b]	V [c]	ΔG_{solv}^{fluid} [d]	ΔG_{cav}^{fluid} [e]	ΔG_{disp}^{fluid} [f]	ΔG_{solv}^{TMG} [g]
**1**	8.3 (± 0.1) × 10^5	–8.08 (± 0.04)	73.28	–0.89	1.97	–2.86	–0.56
**2**	1.5 (± 0.1) × 10^6	–8.41 (± 0.08)	93.57	–1.38	2.26	–3.64	–1.00
**3**	1.6 (± 0.1) × 10^6	–8.47 (± 0.11)	112.88	–1.81	2.53	–4.34	–1.42
**4**	1.4 (± 0.1) × 10^6	–8.40 (± 0.07)	100.79	–2.31	2.36	–4.67	–1.98
**5**	2.0 (± 0.1) × 10^6	–8.60 (± 0.02)	119.55	–2.90	2.62	–5.51	–2.50
**6**	1.4 (± 0.1) × 10^6	–8.40 (± 0.06)	111.37	–2.97	2.51	–5.48	–3.07
**7**	1.5 (± 0.1) × 10^6	–8.44 (± 0.02)	111.34	–2.99	2.51	–5.49	–3.09
**8**	1.2 (± 0.1) × 10^6	–8.31 (± 0.04)	94.36	–3.10	2.27	–5.38	–4.07
**9**	1.7 (± 0.1) × 10^6	–8.50 (± 0.11)	114.06	–3.60	2.54	–6.14	–4.48
**10**	1.3 (± 0.1) × 10^6	–8.34 (± 0.08)	114.10	–3.59	2.54	–6.14	–4.48
**11**	3.4 (± 0.2) × 10^6	–8.90 (± 0.12)	133.43	–4.21	2.80	–7.00	–5.14
**12**	6.1 (± 0.1) × 10^5	–7.89 (± 0.01)	71.20	–2.08	1.94	–4.02	–3.37
**13**	5.1 (± 0.1) × 10^5	–7.79 (± 0.01)	71.14	–2.08	1.94	–4.02	–3.37
**14**	1.3 (± 0.1) × 10^6	–8.33 (± 0.01)	84.11	–3.15	2.13	–5.28	–4.54
**15**	9.8 (± 0.4) × 10^5	–8.17 (± 0.02)	84.15	–3.15	2.13	–5.28	–4.54
**16**	7.9 (± 0.3) × 10^5	–8.04 (± 0.07)	91.23	–2.59	2.23	–4.82	–3.73
**17**	1.6 (± 0.1) × 10^6	–8.47 (± 0.06)	104.13	–3.59	2.41	–6.00	–4.81
**18**	1.6 (± 0.1) × 10^6	–8.48 (± 0.05)	103.75	–3.60	2.40	–6.00	–4.88
**19**	6.4 (± 0.1) × 10^4	–6.56 (± 0.01)	64.21	–2.35	1.83	–4.18	–4.41
**20**	4.7 (± 0.2) × 10^5	–7.73 (± 0.08)	77.61	–3.45	2.03	–5.48	–5.58
**21**	3.2 (± 0.1) × 10^5	–7.50 (± 0.01)	77.66	–3.45	2.03	–5.48	–5.58
**22**	8.1 (± 0.1) × 10^4	–6.70 (± 0.06)	84.31	–2.98	2.13	–5.11	–4.98
**23**	1.5 (± 0.1) × 10^5	–7.08 (± 0.02)	84.46	–2.89	2.13	–5.02	–5.47
**24**	1.7 (± 0.1) × 10^5	–7.15 (± 0.20)	91.11	–4.29	2.23	–6.52	–7.70

[a] Binding affinity [M^{-1}] obtained by ITC. [b] Free energy of guest transfer from aqueous solution to CB[7]. [c] Free energy of solvation of truncated guests **1** – **24** in perfluorohexane. [c] Guest volume [$Å^3$] calculated from a structure delimited by a 0.002 electron/$Bohr^3$ isodensity surface after optimization with the PM6 semi-empirical model. [d] Cavitation free energy required to accommodate the truncated guests in perfluorohexane. [e] Dispersive component of the interaction between the truncated guests and perfluorohexane. [f] Free energy of solvation of truncated guests **1** – **24** in tetramethylglycoluril. All energy terms in kcal/mol.

5. References

[1] F. Diederich, P. J. Stang, R. R. Tykwinski and Editors, in Modern supramolecular chemistry; Strategies for macrocycle synthesis, Wiley-VCH Verlag GmbH & Co. KGaA, 2008.

[2] Software 4 Science Developments (S4SD) - AFFINImeter, Santiago de Compostela, Spain, https://www.affinimeter.com.

[3] A development of University of Karlsruhe and Forschungszentrum Karlsruhe GmbH, 1989-2007, TURBOMOLE GmbH, since 2007; available from http://www.turbomole.com.

[4] S. Ehlert, M. Stahn, S. Spicher and S. Grimme, *J. Chem. Theory Comput.*, 2021, **17**, 4250-4261.

[5] S. Grimme, *J. Chem. Theory Comput.*, 2019, **15**, 2847-2862.

[6] C. Bannwarth, S. Ehlert and S. Grimme, *J. Chem. Theory Comput.*, 2019, **15**, 1652-1671.

[7] S. Grimme, C. Bannwarth and P. Shushkov, *J. Chem. Theory Comput.*, 2017, **13**, 1989-2009.

[8] BIOVIA COSMOtherm, Dassault Systèmes, Paris, France, https://www.3ds.com/products/biovia/cosmo-rs/cosmotherm.

[9] SCM, Amsterdam, The Netherlands, https://www.scm.com.
[10] Wavefunction, Inc., Irvine, CA, USA, https://www.wavefun.com.
[11] F. Weigend, *Phys. Chem Chem. Phys.*, 2006, **8**, 1057-1065.
[12] F. Weigend and R. Ahlrichs, *Phys. Chem Chem. Phys.*, 2005, **7**, 3297-3305.
[13] A. D. Becke, *Phys. Rev. A*, 1988, **38**, 3098-3100.
[14] A. Klamt and G. Schüürmann, *J. Chem. Soc. Perkin Trans. 2*, 1993, 799-805.
[15] J. P. Perdew, *Phys. Rev. B*, 1986, **33**, 8822-8824.
[16] J. P. Perdew, *Phys. Rev. B*, 1986, **34**, 7406.
[17] S. Ehlert, M. Stahn, S. Spicher and S. Grimme, *J. Chem. Theory Comput.*, 2021, **17**, 4250-4261.
[18] S. Grimme, S. Ehrlich and L. Goerigk, *J. Comput. Chem.*, 2011, **32**, 1456-1465.
[19] J. P. Perdew, K. Burke and M. Ernzerhof, *Phys. Rev. Lett.*, 1996, **77**, 3865-3868.
[20] E. van Lenthe, E. J. Baerends and J. G. Snijders, *J. Chem. Phys.*, 1993, **99**, 4597-4610.
